# Randomised controlled trial of tailored support to increase physical activity and reduce smoking in smokers not immediately ready to quit: protocol for the Trial of physical Activity-assisted Reduction of Smoking (TARS) Study

**DOI:** 10.1136/bmjopen-2020-043331

**Published:** 2020-12-01

**Authors:** Adrian Taylor, Tom P Thompson, Michael Ussher, Paul Aveyard, Rachael L Murray, Tess Harris, Siobhan Creanor, Colin Green, Adam Justin Streeter, Jade Chynoweth, Wendy Ingram, Colin J Greaves, Helen Hancocks, Tristan Snowsill, Lynne Callaghan, Lisa Price, Jane Horrell, Jennie King, Alex Gude, Mary George, Charlotte Wahlich, Louisa Hamilton, Kelisha Cheema, Sarah Campbell, Dan Preece

**Affiliations:** 1 School of Medicine, Faculty of Health, University of Plymouth, Plymouth, UK; 2 Division of Population Health Sciences and Education, University of London, St George’s, London, UK; 3 Institute for Social Marketing, University of Stirling, Stirling, UK; 4 Nuffield Department of Primary Care Health Sciences, University of Oxford, Division of Public Health and Primary Health Care, Oxford, UK; 5 School of Medicine, University of Nottingham, Nottingham, UK; 6 College of Medicine and Health, University of Exeter, Exeter, UK; 7 School of Sport, Exercise and Rehabilitation Science, University of Birmingham, Birmingham, UK; 8 Sport and Health Sciences, University of Exeter, Exeter, UK; 9 Public Health, Plymouth City Council, Windsor House, Plymouth, Devon, UK

**Keywords:** primary care, public health, clinical trials

## Abstract

**Introduction:**

Smoking reduction can lead to increased success in quitting. This study aims to determine if a client-focused motivational support package for smoking reduction (and quitting) and increasing (or otherwise using) physical activity (PA) can help smokers who do not wish to quit immediately to reduce the amount they smoke, and ultimately quit. This paper reports the study design and methods.

**Methods and analysis:**

A pragmatic, multicentred, parallel, two group, randomised controlled superiority clinical trial, with embedded process evaluation and economics evaluation. Participants who wished to reduce smoking with no immediate plans to quit were randomised 1:1 to receive either (1) tailored individual health trainer face-to-face and/or telephone support to reduce smoking and increase PA as an aid to smoking reduction (intervention) or (2) brief written/electronic advice to reduce or quit smoking (control). Participants in both arms of the trial were also signposted to usual local support for smoking reduction and quitting. The primary outcome measure is 6-month carbon monoxide-confirmed floating prolonged abstinence following participant self-reported quitting on a mailed questionnaire at 3 and 9 months post-baseline. Participants confirmed as abstinent at 9 months will be followed up at 15 months.

**Ethics and dissemination:**

Approved by SW Bristol National Health Service Research Committee (17/SW/0223). Dissemination will include publication of findings for the stated outcomes, parallel process evaluation and economic evaluation in peer-reviewed journals. Results will be disseminated to trial participants and healthcare providers.

**Trial registration number:**

ISRCTN47776579; Pre-results.

**Strengths and limitations of this study**

- This is the first study to determine whether offering support to increase physical activity alongside smoking reduction is effective and cost-effective in increasing smoking abstinence among smokers not immediately ready to quit but who wish to reduce. The study involves over 900 participants recruited across four sites.
- The study’s primary outcome is biochemically verified 6-month prolonged floating abstinence between 3 and 9 months post-baseline, with a secondary endpoint to consider differences at 15 months.
- The intervention involved considerable public involvement in both the pilot and current trial and was person-centred, theory based, manualised and delivered by eight health trainers who were trained to deliver behavioural support, and subsequently remotely supervised by phone or Skype.
- A mixed-methods process evaluation will explore the fidelity of intervention design, delivery, receipt and enactment, and explore if and how key components in our logic model operated.
- As an effectiveness trial, the intervention does not involve a supervised exercise programme and some participants entering the study may have been more interested in smoking reduction rather than increasing physical activity. This may limit the study’s contribution to the exercise and smoking cessation evidence.

## Introduction

Smoking remains the main cause of preventable morbidity and premature death in high-income nations.^1^ The annual cost of smoking in England is estimated to be £11 billion to society, of which £2.5 billion is to the National Health Service (NHS) in England.^2^ Tobacco control policies and individually targeted interventions have helped to reduce the UK population smoking prevalence rate to 14.7%,^3^ but prevalence varies considerably by socioeconomic and mental health status, contributing to growing health inequalities.

The UK’s National Institute for Health and Care Excellence Public Health No. 10 guidelines^4^ for smoking cessation focus on identifying a quit date and abrupt cessation, with a recognition of the importance of motivational support to supplement pharmacological support. For those not intending to quit immediately, smoking reduction may lead to more quit attempts and subsequent successful abstinence, though there are limitations in the evidence^5–8^ with a wide range of approaches to reduction (eg, pharmacological, behavioural support and self-initiated approaches). Motivational support appears to have the potential to support reduction in smoking, and the greater the reduction the greater the likelihood of successful quitting.^9^

The English Smoking Toolkit Study (2011–2014) suggests there is interest in smoking reduction, rather than immediately quitting, among 50% of smokers, and approximately 30% of UK smokers report using e-cigarettes to do so.^10^ Other evidence-based approaches to self-regulate smoking are needed as there remains uncertainty over the safety of e-cigarettes.^11^ There is considerable interest in encouraging smokers to acutely and chronically use physical activity (PA) to manage smoking^12 13^ though a recent systematic review revealed only 1 of 24 randomised controlled trials provided evidence that an exercise programme can aid abstinence for at least 6 months^14^ among those attempting to quit. That said, most studies were of low quality and focused on proof of concept, rather than being pragmatic and offering an acceptable and feasible intervention for smokers more generally.

A unique randomised pilot study provided promising support for short-term effects of a behavioural support intervention offered by a health trainer for increasing PA and smoking reduction on cigarettes smoked and abstinence.^15 16^ Previous studies^17^ have reported that a self-determination-based intervention to support autonomy and perceived competence for smokers can facilitate long-term smoking abstinence, and this approach was embedded in the pilot intervention. Intervention participants had an average 4.2 sessions by phone or face-to-face with the health trainer, with a range of 0–8 sessions. Compared with the control group, they were significantly more likely to achieve at least a 50% reduction in number of cigarettes smoked (39% vs 20%), to attempt to quit (22% vs 6%), and be abstinent up to 8 weeks after quit day (14% vs 4%) and at 16 weeks (10% vs 4%). A higher proportion of the intervention group also reported using PA for controlling smoking: 55% vs 22% and 37% vs 16%, at 8 and 16 weeks, respectively.^15^ The intervention costs were approximately £192 per participant and exploratory cost-effectiveness modelling indicates that the intervention may be cost-effective.^15^

PA is likely to influence smoking behaviour through both implicit and explicit processes.^12^ A smoker could focus on becoming physically active which in turn has emotional benefits which may implicitly reduce cognitive and emotional triggers for smoking. Exercise may also be explicitly used to acutely manage cigarette cravings and withdrawal symptoms,^18 19^ and chronically manage weight gain associated with smoking reduction or help in a shift towards a healthier identity.

In prospective population surveys and trials, weight gain and fear of weight gain is associated with reluctance to quit smoking and remain abstinent, especially among women and initially heavier smokers,^20–22^ with a meta-analysis study reporting an average of 4.67 kg (95% CI: 3.96 to 5.38) gained after 12 months of abstinence.^23^ There is evidence that PA is effective for preventing long-term weight gain after smoking cessation,^24^ not only by increased energy expenditure and metabolic rate, but also through self-regulation of energy intake, particularly emotional snacking.^25^ Finally, as a result of increasing PA, a smoker may begin to establish a different identity (eg, investing in personal fitness and generally becoming a ‘healthy person’), which in turn may trigger a desire to reduce harm from smoking through reduction and ultimately quitting.^15^

Following a successful pilot study^15^ there is a need to establish the effectiveness and cost-effectiveness of behavioural support for increasing PA and reducing smoking on longer term abstinence among smokers not immediately ready to quit.

## Aims & objectives

The overarching aim of the trial is to determine if an individually tailored behavioural intervention for smokers wishing to reduce but not immediately quit provides an effective and cost-effective approach to supporting increases in PA and smoking reduction, resulting in increased rates of quit attempts and subsequent 6-month prolonged smoking abstinence.

The specific aims of the trial are to determine whether the intervention, compared with support as usual (SAU), will:

- Increase the proportion of participants who achieve 6-month prolonged biochemically verified floating abstinence at 9 months post-baseline.
- Increase the proportion of participants who self-report a reduction in number of cigarettes smoked (between baseline and both 3 months and 9 months) of at least 50%.
- Increase the proportion of participants who achieve biochemically verified prolonged abstinence at 15 months post-baseline (ie, 12 months post-intervention).
- Increase self-reported PA at 3 and 9 months post-baseline, and accelerometer assessed PA at 3 months post-baseline.
- Improve quality of life, self-reported weight and cigarette cravings at 3 and 9 months post-baseline.

Further aims are:

- To conduct a health economic evaluation to estimate the costs of delivering the intervention and differences in health and social care costs between intervention and SAU at 9 months post-baseline. This will also estimate the cost-effectiveness of the intervention compared with SAU at (1) 9 months and (2) over a longer term/lifetime horizon.
- To conduct an embedded mixed-methods process evaluation to explore the mechanisms of action of the intervention and acceptability of study processes.

The current paper outlines the protocol for the Trial of physical Activity-assisted Reduction of Smoking (TARS) trial.

## Methods & analysis

This protocol is reported in accordance with the Standard Protocol Items: Recommendations for Interventional Trials guidance^26^ for protocols of clinical trials and the Template for Intervention Description and Replication guidelines^27^ for intervention description.

### Study design and setting

The TARS trial is a pragmatic, multicentred, parallel, two group, randomised controlled superiority clinical trial to compare (1) tailored support to reduce smoking and increase PA as an aid to smoking reduction (intervention) with (2) brief advice to reduce or quit smoking (control). The study includes a mixed-methods embedded process evaluation and health economics evaluation. Recruitment to the trial was over 16 months (January 2018–May 2019) in the geographical areas in and around four cities in the UK: Plymouth, Nottingham, London and Oxford.

### Study population

Potential participants were approached from primary and secondary care establishments and also from the community. Participants were adult smokers (≥18 years) who smoked ≥10 cigarettes per day (for at least 1 year), wished to reduce smoking but not quit immediately. We accepted smokers who were also using other nicotine-containing or other cigarette management products. Smokers were ineligible if they were unable to engage in at least 15 min of moderate intensity PA, had any illness or injury that might be exacerbated by exercise, were unable to engage in the study and/or the intervention due to language or for other reasons (eg, provide an unacceptable level of risk to the health trainer or researcher). All ineligible smokers were referred for smoking cessation advice in line with local usual practice.

### Study procedures

#### Participant identification, approach and consent

A broad range of participant identification and approach methods were employed in an attempt to reach many different demographic groups and make the study as inclusive as possible. The most common approach method was through a search of a primary care practice patient database, with invitations sent to patients who were listed as smokers offering them the chance to get in touch with the trial team. In the early phase of recruitment, a study within a trial was conducted at general practitioner (GP) practices at one research site, to compare the efficiency and cost-effectiveness of three different invitation methods (full information pack, single-page invite, text message).^28^ Other approaches were via secondary healthcare and stop-smoking services (for those who had failed to quit), and by social media, pharmacies, dental practices, community organisations and local businesses using posters and leaflets.

**Figure 1:**
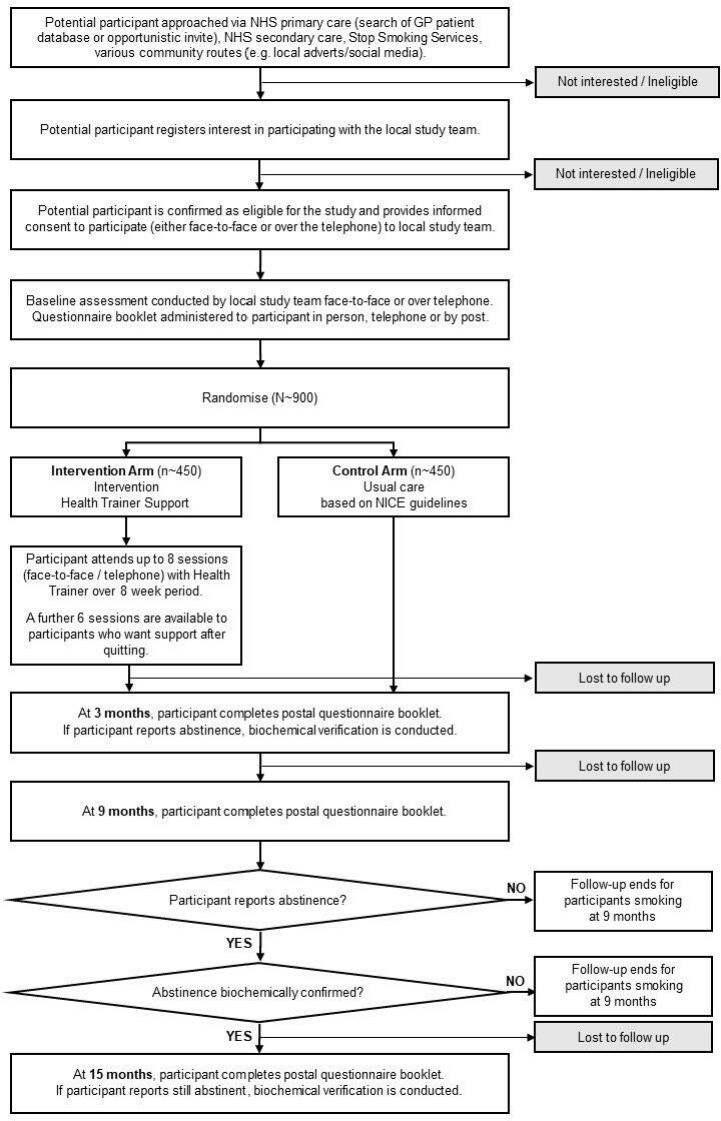
Participant flow chart. GP, general practitioner; NHS, National Health Service; NICE, National Institute for Health and Care Excellence.

As shown in figure 1 (participant flow chart), smokers expressing interest in the trial were screened for eligibility by researchers at each site. If suitable and still interested in joining the study, participants provided evidence of consent either at a face-to-face meeting or via telephone.

#### Baseline assessment

Participants then completed a baseline questionnaire with a member of the local study team either face-to-face, by mail or over the telephone. The schedule of measures and data collection at baseline and follow-up are shown in table 1.

**Table 1:** Schedule of baseline and follow-up measures

	Screening and baseline	Month 3	Month 9	Month 15*
Demographics (eg, age, gender, education attained, employment status)	**X**			
Self-reported cigarettes per day (or equivalent)	**X**	**X**	**X**	**X**
Reduction of ≥50% in number of cigarettes smoked since baseline†		**X**	**X**	
Biochemically confirmed abstinence (self-reported quitters only)		**X**	**X**	**X**
Self-reported floating prolonged abstinence (since quitting smoking, with quit date, if relevant) over at least 6 months†			**X**	**X**
Accelerometer assessed minutes of moderate and vigorous physical activity in a subsample		**X**		
Self-reported 7-day recall of physical activity	**X**	**X**	**X**	
Heaviness of Smoking Index	**X**			
Use of smoking management products	**X**	**X**	**X**	**X**
Urge and strength of urge to smoke	**X**	**X**		
Engagement with the health trainer intervention (8 weeks, plus optional 6 weeks additional support if a quit attempt is made)		**X**		
Health and social care utilisation	**X**	**X**	**X**	
Health-related quality of life (EQ-5D-5L and SF-12)	**X**	**X**	**X**	
Self-reported weight and height (to calculate BMI)	**X**	**X**	**X**	
Self-reported process measures	**X**	**X**		
Importance and confidence in smoking reduction and cessation				
Importance and confidence in being physically active				
Action planning to change smoking				
Action planning to change physical activity				
Self-monitoring of smoking				
Self-monitoring of physical activity				
Availability of support to reduce smoking				
Availability of support to increase physical activity				
Use of physical activity for smoking regulation				
Serious adverse events (self-reported)		**X**	**X**	
Qualitative process evaluation (in parallel throughout) (sample)	**X**	**X**	**X**	**X**

*Only participants with biochemically verified abstinence at 9 months are followed-up at 15 months post-baseline.

†Derived measure.

BMI, body mass index; EQ-5D-5L, EuroQol-5 dimension-5 level; SF-12, 12-Item Short Form Health Survey.

#### Randomisation

Participants were individually randomised to either the intervention or control group (1:1 ratio) following completion of baseline assessments, to ensure concealment was preserved. Randomisation was achieved by means of a 24-hour web-based system created by the UKCRC-registered Peninsula Clinical Trials Unit (CTU) in conjunction with a statistician independent from the trial team, and used random permuted blocks, with stratification for recruitment site and a dichotomised low/high score derived from the Heaviness of Smoking Index (HSI).^29^ The HSI score is calculated from summing responses to two questions:

1. When the first cigarette is smoked after waking, scored as >60 min (score 0); 31–60 min (1); 6–30 min (2) and within 5 min (3).
2. How many cigarettes are smoked in a typical day, scored as ≤10 cigarettes (0); 11–20 cigarettes (1); 21–30 cigarettes (2); >30 cigarettes (3).

Total HSI scores of 0–4 were categorised as low, and 5–6 were considered high for the purposes of stratification.

Following randomisation, all participants were sent a letter from the coordinating CTU confirming which trial arm they had been assigned to, and a guidance sheet on usual support locally for smoking reduction and cessation. The participant’s GP was also sent a letter notifying them that one of their patients is participating in the study.

#### Blinding

It was not possible to blind participants to their allocated group. Every effort was made to ensure that the trial team remained blind to the allocation of each participant when collecting follow-up data (including researchers collecting carbon monoxide (CO) measurements), but this was not always possible. Health trainers delivering the intervention were obviously aware of the participant’s allocation to trial arm, but were discouraged from communicating with site researchers about this. Questionnaire booklets and accelerometers were mailed out from and returned to the CTU without knowledge of trial arm allocation.

#### Follow-up

At 3 and 9 months post-baseline all participants are posted a questionnaire booklet and a freepost envelope to return the completed booklet to the CTU. A £20 shopping voucher is mailed to participants on CTU receipt of the completed booklets at both 3 and 9 months. To increase response rates, motivational postcards are mailed to participants before the follow-up questionnaires are sent out. Up to two reminder letters are issued (and a further three telephone calls as required) to remind participants to return the questionnaire booklets, and the option of the participant telephoning a member of the research team to aid completion of the booklets is offered. Participants are given the option to just complete the key questions about smoking behaviour if the questionnaire booklet is not returned to the CTU within 2 weeks and to submit these responses by email, phone or text if preferred to maximise follow-up data on key outcomes. If participants do not complete the key questions (regardless of method) within a 4-week window, they are categorised as not completing follow-up at that time point.

All participants who report having made a quit attempt in the questionnaire booklet and not smoked since that date at 3 and 9 months are contacted for biochemical verification of abstinence.

At 3 months post-baseline, approximately 20% of participants were sent wrist-worn waterproof accelerometer (GeneActive Original accelerometer, Activinsights, Kimbolton, UK, http://www.geneactiv.org/) with instructions to wear constantly for 1 whole week (day and night), and a freepost return envelope to be sent to the CTU. To maximise data completeness, participants scheduled to receive an accelerometer were sent a standardised letter from the CTU, 2 weeks before receiving the accelerometer, advising them that they would shortly be receiving the device, and asking them to inform the CTU if they were unable to wear it. A letter was sent to participants who did not object to wearing the device, 3 days into the 10-day recording period prompting participants to start wearing the device if they had not already done so. Up to two reminder letters and a follow-up phone call were made to participants if they did not return the accelerometer.

### Trial allocation groups

#### Intervention

The TARS intervention was based on the intervention developed for the EARS pilot trial, with further refinement based on feedback from the EARS process evaluation.^15^ Throughout the development of the intervention for the EARS and TARS studies, we engaged with smokers from a wide range of socioeconomic backgrounds to ensure an acceptable person-centred approach was embedded.

Throughout the pilot trial and before commencing the definitive trial, we conducted individual and focus group discussions with smoking-cessation practitioners, researchers, public health consultants, community workers (including volunteers) and people who currently or previously smoked. We reviewed literature on using exercise as an aid to quitting, and consulted with academic experts on behaviour change for physical activity, smoking reduction and smoking cessation. These activities informed the intervention principles and theoretical basis, structure and delivery.

The intervention aimed to be empowering and put the client at the centre of the decision-making process. All aspects were designed to promote self-determined behaviour, focused on elements of self-determination theory which emphasises people’s sense of autonomy, competence and relatedness.^30–32^ This was in part achieved by adopting motivational interviewing principles^33^ as the guiding delivery style for the practitioners which have been proposed to enhance and promote self-determined behaviour.^34^

**Figure 2:**
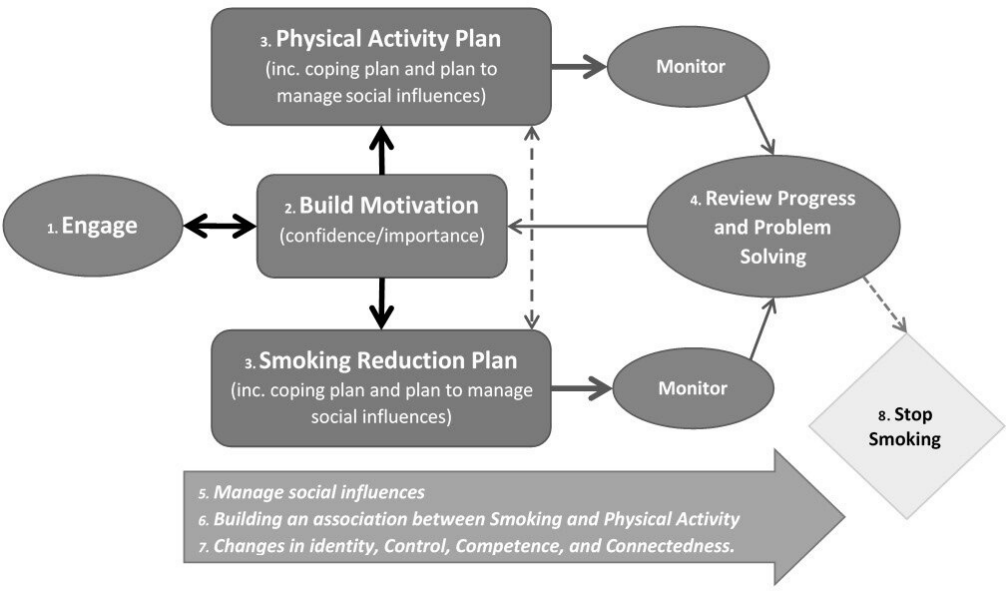
Indicative map of the TARS intervention components. TARS, Trial of physical Activity-assisted Reduction of Smoking.

An intervention delivery model, or ‘roadmap’, was developed between the pilot and definitive trial (figure 2) to aid in the conceptualising of the intervention process and support the manualisation of the intervention and training of the practitioners. This was supported by the development of a set of ‘core competencies’ (table 2) developed from the pilot trial which outlined key processes, components and behaviour change techniques that the health trainers were expected to deliver and form the basis of fidelity assessment.^35^ A comprehensive training manual was developed, outlining all the skills, behaviour change techniques and strategies to support behaviour change intended for use in the TARS Study. This was used as the basis for a 3-day training course, which was delivered by TPT, AT, CG and LC. Health trainers then engaged with a wide range of ‘practice participants’ to complete their training. The health trainers attended regular (bi-weekly for the first 3 months and monthly thereafter) 2-hour formative feedback supervision teleconferences throughout the study period to help to embed skills and to benefit from each other’s shared experiences. Individual supervision for the health trainers was available as needed, provided by the intervention lead (TPT).

**Table 2:** Intervention components, aims, content and indicative change in processes

Intervention components	Aim	Content	Indicative change in processes
Active participant involvement (1)	Develop rapport, build trust and shared respect.	Effective communication skills. Build autonomous support.	Participant feedback on health trainer-led support.
Build motivation to reduce smoking (2) and increase physical activity (3)	Identify ambivalence towards reduction and quitting. Build self-awareness and confidence to cut down and increase physical activity.	Help smoker to identify importance and challenges of reduction and cessation, and implicit and explicit roles of physical activity (motivational interviewing techniques).	Smoker has desire and confidence to cut down and perhaps quit over the early sessions, and increase physical activity. Smoker engages in more self-monitor of smoking and physical activity behaviour.
Self-monitor smoking and physical activity and set goals to reduce smoking (4) and increase physical activity (5)	Develop strategies to reduce smoking and increase physical activity.	Set SMART goals to reduce smoking and increase physical activity. Signpost to physical activity opportunities and remove barriers to do physical activity.	Goals identified and action plans developed. Smoker engages in more goal setting to reduce smoking and increase physical activity behaviour.
Review/problem solving for smoking (6) and physical activity (7)	Build confidence, perceptions of control and self-regulation skills.	Smoker reflects on smoking reduction and physical activity, identifies barriers and possible solutions, increases and sets new targets; perhaps to quit.	Goals revised to reflect confidence to increase physical activity, reduce smoking and possibly quit.
Integrating idea of changing smoking and physical activity (8)	To help smoker to identify any links between smoking and physical activity	Explore with smoker how physical activity may influence smoking (and vice versa) (person centred exchange of information (Ask–Tell–Discuss)).	Smoker increases use of physical activity as an aid to smoking reduction.
Reinforce health identity shift (9)	To help identify shift from smoker to healthier identity.	Smoker reflects on label as heavy–moderate–light or non-smoker status, and more active person.	Decrease in importance of smoking and increase in importance of doing physical activity identified.
Manage social influences on smoking (10) and physical activity (11)	To involve others in process of reducing smoking and increasing physical activity. Manage negative or undermining social influences.	Smoker identifies key others who can support reduced smoking (or cessation) and increasing physical activity, and engages with them in preferred ways. Uses negotiation and discussion to manage negative social influences.	Support from others identified as important and used for smoking reduction or cessation, and increasing physical activity.

Participants allocated to the intervention arm were offered individually tailored behavioural support from a health trainer. The health trainer delivered the processes outlined in figure 2 and table 2, with the option of up to eight weekly sessions, and a further six sessions if the participant wanted support after quitting, and aimed to empower participants to decide what support was offered, and where, when and for how long. Signposting to local smoking cessation support services was also offered to those wishing to quit. If a smoker wished to reduce smoking using e-cigarettes or licensed nicotine-containing products (LNCP), they were also offered any local available support for this.

Health trainers were appointed on the basis of having good communication skills, including empathy, and at least some training and experience in supporting health behaviour change. All health trainers had at least a first degree in a related field although this was not a prerequisite for the role. The health trainer was trained to support change in both smoking and PA and help individuals to make the connections between the two. As described in table 2, the core intervention processes that the health trainer was trained to deliver were: (1) building rapport and supporting autonomous behaviour change; (2) building motivation; (3) supporting self-monitoring and goal setting; (4) problem solving; (5) integrating smoking and PA behaviour; (6) supporting a health identity shift; (7) supporting the management of social influence on behaviours.

The support offered was broadly structured (based on delivering the processes outlined in table 2 and figure 2) but health trainers were trained to be flexible in their approach, to tailor it for individual needs and preferences. They were trained to do this using a person-centred approach and principles from motivational interviewing such as showing empathy and reflective listening.^36^ They were also trained to assess (and be aware of) participants’ needs in relation to the core psychological needs posited by Self-Determination Theory,^37^ which we referred to in the training as ‘the three Cs’: Control (having choice/autonomy in decision-making around behaviour change); Competence (developing self-efficacy/building confidence in the ability to change) and Connectedness (social acceptance of the new behaviour/support from important others for making changes).

A fuller intervention description, including the trainer manual and intervention materials, will be published as supplemental materials linked to the final National Institute for Health Research project report.

#### Support as usual

Participants allocated to both arms of the trial received written guidance for smoking reduction and cessation, including web links to what is offered at local level, or paper versions of this information. Typically, there are no formal programmes for use of medication to support reduction (rather than abrupt stopping) and people usually buy their own replacement therapy or e-cigarette product.

### Determination of sample size

Since the planned primary analysis is a comparison of the proportions of the binary primary outcome, the sample size calculation was based on a two-sided Fisher exact test. An abstinence rate of 5% for the control group and detectable effect of 6% (ie, an increase from 5% to 11% due to the intervention) are conservative estimates consistent with those from the EARS pilot study^15^ and those reported from a systematic review of pharmacological interventions.^38^ The corresponding OR for this effect size is 2.35. Participants with missing outcome data will be assumed to be still smoking following the Russell Standard,^39^ and the numbers of participants in each allocated group are assumed to be in the ratio of 1:1. Under these conditions, according to Stata V.14.2, the minimum number of participants required to detect an abstinence rate of 11% compared with that of 5% in the control group, with a significance level of no more than 5% and power of at least 90%, is 900, above which a power in excess of 90% is maintained.

### Outcome measures

Table 1 lists the outcome measures and when they are being assessed.

#### Primary outcome measure

Biochemically verified 6-month floating prolonged abstinence between 3 and 9 months.^40^ Abstinence will be confirmed by expired CO <10 ppm measured with a CareFusion MicroCO metre (Williams Medical Supplies, Rhymney, UK, www.carefusion.co.uk) at a face-to-face visit.

Participants who self-reported abstinence at 3 months and who were confirmed as abstinent through biochemical verification via expired CO at a face-to-face assessment and then self-report abstinence (and not having smoked even a puff since the 3-month assessment) at 9 months, again confirmed by expired CO, will be defined as having prolonged abstinence over at least 6 months.

#### Other smoking-related measures

Only participants who have biochemically verified abstinence at 9 months are being contacted at 15 months post-baseline to assess floating prolonged abstinence over a period of 12 months (3–15 months). Participants who were not abstinent at 3 months but have biochemically confirmed abstinence at 9 months will be contacted at 15 months to confirm floating prolonged abstinence of 6 months between 9 and 15 months. As a contingency measure for verification of abstinence during the COVID-19 outbreak, abstinence will be confirmed by saliva cotinine level <12 ng/mL^41^ using a mailed self-collection kit and assay provided by ABS Laboratories (York, UK, www.acmgloballab.com). This contingency measure will apply to follow-up for secondary outcomes for a minority of participants. While the intervention is expected to primarily influence quitting in the first 3 months of the study, it is possible that a sustained quit attempt occurs after the 3-month assessment as a result of the health trainer building behaviour change skills which are used subsequently to reduce and then quit smoking.

Participants are asked to self-report the number of cigarettes smoked and type of nicotine product (ie, pipes, cigars and roll your own). We estimated 0.45 g of tobacco was the equivalent of one cigarette based on a previous rigorous study^42^ and reported in the EARS pilot study.^15^ From this we will estimate the number of cigarettes smoked at follow-up, and also calculate if participants reduce smoking by at least 50% between (1) baseline and 3 months, and (2) baseline and 9 months.

#### PA measures

The 7-day recall measure of PA^43^ is used to assess self-reported weekly minutes of moderate and vigorous PA. An objective measure of total weekly minutes of moderate and vigorous PA (MVPA) was collected with a wrist-worn waterproof GENEActiv accelerometer^44^ around the 3-month follow-up time point. Participants were asked to wear the accelerometer on the wrist of the non-dominant hand constantly for 1 week and then return to the CTU. This accelerometer shows the wearer no information about their PA levels and does not have obvious motivational value.

#### Physical measures

Participants are asked to self-report their height and weight from which body mass index (BMI) will be calculated.

#### Health-related quality of life measures

The EuroQol-5 dimension-5 level (EQ-5D-5L) (EuroQol Group, 1990) comprises the following five dimensions: mobility, self-care, usual activities, pain/discomfort and anxiety/depression. Each dimension has five levels: no problems, slight problems, moderate problems, severe problems and extreme problems.^45^ The 12-Item Short Form Health Survey is a 12-item, patient-reported survey of patient health, consisting of 12 questions.^46^

#### Resource use/healthcare service use

Use of primary and community-based health and social services, and hospital-based inpatient and outpatient services are captured using a resource use questionnaire developed in two pilot trials involving health trainer-led interventions.^16 47^ It sought to capture the number of contacts that occurred (if any) with a range of health and social care professionals and where those contacts took place since completing the previous survey, using both fixed and open format responses. Reasons for hospital admissions were also requested.

#### Self-reported process measures

Single-survey items, using a 5-point Likert scale (strongly disagree to strongly agree) assessed the psychological and behavioural processes that the intervention was designed to influence as shown in table 2. These were used in other trials.^15 48 49^ A single-survey item, using a 6-point scale (not at all to all the time), was used to assess frequency of urge to smoke in the past week.^50^ A single survey item (6-point Likert scale, no urges to extremely strong) assessed strength of urges to smoking in the past week^50^ as used previously.^15^

### Economic evaluation

The economic evaluation will (1) estimate the long-term cost-effectiveness of the TARS intervention plus SAU versus SAU alone, over a lifetime horizon using a model-based economic evaluation, and (2) estimate the cost-effectiveness of the TARS intervention plus SAU versus SAU alone over the primary 9-month trial follow-up, in an economic evaluation conducted alongside the trial. The longer term model-based economic analysis is considered the primary economic analysis, consistent with the approach commonly applied in the context of cost-effectiveness analysis in smoking cessation settings.

The primary perspective of the economic analyses will be that of the NHS and Personal Social Services (ie, third party payer), with a broader perspective explored in sensitivity analyses. The primary economic endpoint will be the quality-adjusted life-year (QALY, using EQ-5D data), with results presented as incremental cost-effectiveness ratios representing estimated costs per QALY gained.

The economic analysis will be undertaken against a predefined health economics analysis plan, which is available on request. In summary, the trial-based cost-effectiveness analysis will use participant-level data collected within-trial to estimate (1) the resource use and costs associated with the delivery of the TARS intervention, (2) broader resource use and costs associated with health and care service use by group, (3) QALYs by group, and (4) the incremental cost per unit of outcome (eg, cost per incremental QALY, cost per quitter) over the 9-month follow-up. In this analysis, EQ-5D-5L data will be used to estimate QALYS, deriving health state values at each time point, using the published tariff values for England (presently recommendations are for values to be derived using methods reported by van Hout *et al)*,^51^ and using the area under the curve approach.^52^ Analyses will be based on an intention-to-treat (ITT) principle, using a complete case analysis, and will assess uncertainty and include detailed sensitivity analyses.

The model-based economic evaluation will adopt a longer term perspective (lifetime) beyond the trial follow-up, to present a policy relevant cost-effectiveness analyses, that predicts future costs and QALYs after the trial endpoint based on the reported effectiveness of the TARS intervention. A decision analytic model will be used, with the model based on the model developed and described in the prior pilot study (EARS),^15^ which we will update and adapt using a review of the recent literature on modelling in this area and based on input from a stakeholder group. The model-based evaluation will be based on good practice guidelines for decision analytic modelling in the health technology assessment context.^53–55^

### Embedded mixed-methods process evaluation

A mixed-methods process evaluation will focus on trial processes and methods, and will attempt to understand the effective components and processes of the intervention. During the internal pilot phase, the evaluation will focus on barriers and facilitators for recruitment methods, initial intervention engagement and early intervention implementation. For the subsequent main trial, the evaluation will focus on acceptability of study processes (via a qualitative substudy), intervention engagement levels, predictors of intervention engagement, intervention delivery fidelity and evaluating the implementation of the intervention process model (participant understanding of the intervention model (receipt fidelity), mediating effects of process measures on PA and smoking outcomes (enactment fidelity), mediating effects of PA on smoking outcomes, approaches and acceptability of smoking reduction, multiple behaviour change, progression to cessation and other perceived effectual intervention components).

Data for the process evaluation will be collected via: the trial database, audio recordings of intervention sessions, and audio recorded and transcribed interviews with trial participants, research assistants, health trainers and GPs/practice managers.

### Trial data handling

Data are collected and maintained in accordance with the current legal and regulatory requirements. A data management protocol has been produced by the CTU to ensure secure data collection and storage in accordance with the Data Protection Act 1998, and later conforming to the General Data Protection Regulation 2016 and Data Protection Act 2018.

Electronic study records will be held over the lifetime of the study in secure storage solutions aligned with the host institution’s information security classification policy. At the time of writing, electronic study data are stored in an SQL server database on a restricted access, secure server maintained by the University of Plymouth. Data are entered into the database via a bespoke web-based data entry system encrypted using SSL V.3 (QuoVadis Global, http://www.quovadisglobal.com). Access to identifiable information is restricted and permission-based.

A parallel, linked, bespoke data system has been used to manage intervention engagement post-randomisation. The system captures all health trainer attempted and actual contact with intervention participants in real time to produce summary data (eg, number of sessions, duration and mode of sessions, notes on session content), and aid supervision sessions and intervention management (eg, should a health trainer be unavailable).

Identifiable information will be omitted from the transcriptions of the process evaluation interviews.

### Statistical analysis plan

A detailed statistical analysis plan was drafted during the trial delivery phase and will be approved by an independent statistician and wider Trial Steering Committee (TSC), prior to database lock. The analyses will be reported in full and in accordance with the Consolidated Standards of Reporting Trials (CONSORT) guidelines.^56^ The main planned analyses are summarised below.

### Baseline characteristics and summary statistics

Descriptive statistics by allocated group will be presented for the baseline, and primary and secondary outcomes, which includes the smoking outcomes and questionnaire data as well as the smoking and physical mediators, with the exception of the primary outcome and secondary abstinence measures (assessed at 3, 9 and 15 months only) and accelerometer outcomes (assessed only at 3 months).

For continuous outcomes, summary information will be presented in the form of means alongside SDs. Count and skewed continuous data will be presented in terms of median and IQR. For categorical outcomes, summary information will be presented in the form of frequencies and percentages.

Inferential statistical comparison at baseline of randomised groups is not good practice^57^ and it is expected that participants in both groups will, on average, be similar. Following initial primary analysis, if substantial imbalance at baseline is identified in any key variables, such as gender and age, the importance of any imbalance will be noted and additional adjusted analyses may be performed.

### Primary analysis

The null hypothesis is that there is no difference in CO verified 6-month prolonged floating abstinence rates between the intervention and control groups at 9 months post-baseline. In line with the Russell Standard schedule,^39^ the primary comparative analysis will be conducted on an ITT basis, in which participants with missing responses will be considered to still be smokers. Interpretation of the primary effectiveness analysis will be based on the OR from the logistic regression model adjusted for (fixed effect) stratification variables: site as a factor and HSI as an ordinal covariate. Both the adjusted (primary analysis) and unadjusted ORs and corresponding 95% CIs will be presented. Primary effectiveness shall also be presented as a relative risk along with the absolute between-group differences in abstinence rates, as recommended in the CONSORT guidelines for parallel group randomised trials.^58^

Planned sensitivity analysis of the primary outcome:

- Rather than assuming participants with missing responses at 3 or 9 months were still smoking, the primary outcome will be imputed under a number of varying assumptions and the primary analysis re-run for each of the scenarios.
- A complier average causal effect analysis will be undertaken, if greater than 20% of participants allocated to the intervention group are categorised as not having completed at least two intervention sessions with a health trainer, with individual participants in the intervention group categorised as compliers if they completed at least two intervention sessions. Participants in the control group and non-compliers in the intervention group will be compared with compliers in the intervention group.

### Secondary analyses

To explore whether the primary outcome was influenced by the intervention dose actually received (ie, number of health trainer sessions attended), the primary outcome shall be modelled on the number of health trainer sessions attended in the intervention group only, adjusting for the stratification variables. Although the trial is not powered to detect the influence of moderating factors on the primary outcome, secondary analyses will be undertaken to explore whether the intervention effect is modified by key demographic and/or behavioural factors at baseline. These are prespecified as the postcode-based Index of Multiple Deprivation (IMD); the factor indicating smoking cessation medication or a vaping product at baseline; MVPA level, confidence to quit; and the stratification variable, HSI. The multivariable logistic regression model outlined above will be extended to include the interaction term of allocated group and each of the listed potential modifying variables. Evidence of an interaction shall be interpreted through the 95% CIs of the coefficient for the interaction term.

During the development of this study, the potential health trainer effect was considered at length. Given the lack of evidence on individual health trainer effects, the study design and sample size calculations do not allow for such partial clustering within health trainers (within recruitment sites). However, an exploratory analysis of the intervention effect will be undertaken using a multilevel, mixed-modelling approach, to allow for the partially nested data: participants allocated to the intervention group will be partially clustered within the health trainer, in turn nested within sites.

#### Analysis of secondary outcomes

Between-group comparisons will be undertaken, including for the following key secondary outcomes:

- Biochemically verified point prevalence abstinence at 3, 9 and 15* months post-baseline.
- Self-reported point prevalence abstinence at 3, 9 and 15* months post-baseline.
- Prolonged biochemically verified abstinence over 6 months between 9 and 15 months post-baseline.
- Prolonged biochemically verified abstinence for at least 12 months between 3 and 15 months post-baseline (derived from biochemically confirmed abstinence at all three follow-up time points).
- At least a 50% reduction in reported smoking levels between (1) baseline and 3 months, and (2) baseline and 9 months.
- Number of cigarettes used on an average day over the past week (including equivalent cigars, tobacco) at 3, 9 and 15* months post-baseline.
- Total number of LNCP used on an average day over the past week at 3, 9 and 15* months post-baseline.
- Total number of self-reported minutes engaged in MVPA over the past week at 3 and 9 months post-baseline.
- BMI at 3 and 9 months post-baseline.

NB: * only for those with biochemically confirmed abstinence at 9 months.

The following data are derived from accelerometers mailed to a subgroup of participants in both arms of the trial along with the 3-month questionnaire, which are returned after the accelerometers have been worn for 7 days:

- The average time spent in moderate to vigorous activity over the past week.
- The average daily time spent sleeping over the past week.

No adjustment for multiple analyses will be made; such adjustment methods are too conservative when outcomes are positively correlated, as they would be in this trial. Analyses will use multivariable linear regression (continuous outcomes) or logistic regression (binary outcomes) to compare each of these secondary outcomes between allocated groups, with adjustment for site as a factor, and HSI, as an ordinal variable, as well as baseline values of the outcome as appropriate. As accelerometer data were only available at 3 months, only summary statistics for weekly minutes of MVPA shall be presented by allocated group without adjustment for baseline variables.

The between-group comparisons of continuous outcomes will be reported as mean differences together with 95% CIs, unless the outcomes are substantially skewed. Both adjusted and unadjusted differences will be presented. The between-group comparisons of binary outcomes will be reported as the adjusted and unadjusted ORs with conversion to relative risks and corresponding CIs, along with the absolute between-group differences in abstinence rates.

Analyses will be undertaken to investigate whether any effect of the intervention in terms of reduction in smoking at 3 months and 9 months is modified by key sociodemographic and/or behavioural factors at baseline. These prespecified factors at baseline are using smoking management medication or vaping, IMD, the stratification variables, MVPA level and confidence to quit. The multivariable models will be extended to include the interaction term of allocated group and each of the potential modifying variables.

#### Model checking

The logistic regression model for the primary analysis is prespecified. However, observations identified as potential outliers through their influence on the model, may be excluded as part of a sensitivity analysis. The distributional assumptions of multivariable linear regression models will be visually assessed through plots of residuals. If there are concerns about distributional assumptions being met, bootstrapped CIs for the adjusted between-group differences will be produced.

### Mediational analysis

If there is evidence that the intervention is effective, an exploratory mediational analysis will be undertaken to determine if any effect of allocated group on the primary outcome was mediated by changes in smoking and/or physical activity between baseline and 3-month follow-up, adjusted for the stratification variables (site and HSI). Further analyses will explore if changes in smoking and physical activity are mediated by changes in outcomes from the questionnaire on attitudes to smoking between baseline and 3 months (importance and confidence to reduce smoking/increase physical activity; self-monitoring and goal setting; urges to smoke).

## Discussion

The manuscript describes the methods involved in the trial design and intervention delivered.

### Patient and public involvement

The TARS research team has worked with people who smoke, not as research participants, individually and in groups from across all communities, to guide research questions and study design, and conduct intervention development and dissemination over the past 15 years. As an example of their input, they had varying views on the merits of vaping to reduce smoking and how various forms of PA may help. A university employee and non-employee patient and public involvement (PPI) group (of people who currently or have previously smoked) regularly met to input into intervention and trial methods development. There was further PPI input into Project Management Group (PMG) meetings and TSC meetings throughout the trial. The study team has also engaged with key stakeholders involved in commissioning and delivering community interventions to assess where the proposed intervention would best fit and the study team will continue to do this prior to and during intervention development.

### Trial monitoring and oversight

The trial PMG includes a multidisciplinary team of clinicians and researchers with expertise in all aspects of trial design, intervention development and delivery, conduct, analysis and quality assurance. The TSC involves independent expertise to help guide the trial on behalf of the funders and provide oversee trial progress. The TSC will also sign off the Statistical and Health Economic Analyses Plans. The Data Monitoring Committee (DMC) provides independent expertise and support particularly regarding evidence or reason why the trial should be amended or terminated based on recruitment rates, compliance, safety or efficacy.

### Ethics and dissemination

The study has been approved by the South West–Central Bristol Research Ethics Committee (REC reference: 17/SW/0223) and the Health Research Authority. A number of approvals have been granted for minor and substantial amendments; the amendment history and full details of the amendments are available on request.

The research team will work with stakeholders and PPI representatives at each site, and nationally, to help to interpret the results and the implications for policy and practice. The PMG will establish a publication plan and authorship rules. Reporting will follow current CONSORT guidelines for randomised trials. The study results will be submitted for publication in relevant international, high impact, peer-reviewed journals. Names of key collaborators and groups who have contributed to the trial will be clearly stated in all publications. The study findings will be presented at regional, national and international meetings as appropriate.

### Safety considerations

The recording and reporting of non-serious adverse events (AEs) in this low-risk study is not required. Serious AEs (SAEs) will be documented from the time of participant consent until a maximum of 8 weeks after the follow-up assessment at 9 months. A protocol for identifying, reporting and managing SAEs has been established by the CTU, in conjunction with the PMG, DMC and TSC, and conforms to the requirements of the trial sponsor and NHS REC.

### Access to data

The CTU Data Manager is the custodian of the final trial data set, with the exception of the audio files and transcripts arising from qualitative interviews with participants which are held by the process evaluation team.

Access will be granted to the sponsor and host institution for the purposes of study-related monitoring, audits and inspections.

Members of the study team will have restricted access to the anonymised dataset for the purposes of conducting the trial, and to undertake the statistical and economic data analysis and the process evaluation.

Data requests should be submitted to the corresponding author for consideration. Following publication of the primary results of the trial, access to available anonymised data may be granted depending on review of the data request and appropriate agreements being in place.

### Current study status

The TARS Study completed participant recruitment in May 2019. Data collection for the 9-month and 15-month follow-up assessments are expected to be completed in April and October 2020, respectively, and results are expected to be published in mid-2021.

## Supplementary Material

Reviewer comments

Author's manuscript
