# Supplementary material for: Randomised controlled trial of tailored support to increase physical activity and reduce smoking in smokers not immediately ready to quit: protocol for the Trial of physical Activity-assisted Reduction of Smoking (TARS) Study
Source: BMJ Open. 2020 Dec 1;10(12):e043331. doi: 10.1136/bmjopen-2020-043331 (PMC7709511; doi:10.1136/bmjopen-2020-043331)
Supplement: Author's manuscript [file bmjopen-2020-043331.draft_revisions.pdf]

BMJ Open

BMJ Open is committed to open peer review. As part of this commitment we make the peer review history of every article we publish publicly available.

When an article is published we post the peer reviewers' comments and the authors' responses online. We also post the versions of the paper that were used during peer review. These are the versions that the peer review comments apply to.

The versions of the paper that follow are the versions that were submitted during the peer review process. They are not the versions of record or the final published versions. They should not be cited or distributed as the published version of this manuscript.

BMJ Open is an open access journal and the full, final, typeset and author-corrected version of record of the manuscript is available on our site with no access controls, subscription charges or pay-per-view fees (http://bmjopen.bmj.com).

If you have any questions on BMJ Open's open peer review process please email info.bmjopen@bmj.com

BMJ Open

A randomised controlled trial of tailored support to increase physical activity and reduce smoking in smokers not immediately ready to quit: Protocol and baseline participant characteristics for the Trial of physical Activity assisted Reduction of Smoking (TARS) study

Journal:	*BMJ Open*
Manuscript ID	bmjopen-2020-043331
Article Type:	Protocol
Date Submitted by the Author:	04-Aug-2020
Complete List of Authors:	Taylor, Adrian; University of Plymouth, Faculty of Health: Medicine, Dentistry & Human Sciences<br>Thompson, T; University of Plymouth, Faculty of Health: Medicine, Dentistry & Human Sciences<br>Ussher, Michael; University of London St George's, Division of Population Health Sciences and Education; University of Stirling, Institute for Social Marketing<br>Aveyard, Paul; University of Oxford Division of Public Health and Primary Health Care, Nuffield Department of Primary Care Health Sciences<br>Murray, Rachael; University of Nottingham Faculty of Medicine and Health Sciences, School of Medicine<br>Harris, Tess; University of London St George's, Division of Population Health Sciences and Education<br>Creanor, Siobhan; University of Plymouth, Faculty of Health: Medicine, Dentistry & Human Sciences<br>Green, Colin; University of Exeter, College of Medicine and Health<br>Streeter, Adam; University of Plymouth, Faculty of Health: Medicine, Dentistry & Human Sciences<br>Chynoweth, Jade; University of Plymouth, Faculty of Health: Medicine, Dentistry & Human Sciences<br>Ingram, Wendy; University of Plymouth, Faculty of Health: Medicine, Dentistry & Human Sciences<br>Greaves, Colin; University of Birmingham School of Sport Exercise and Rehabilitation Sciences, School of Sport, Exercise and Rehabilitation Science<br>Hancocks, Helen; University of Plymouth, Faculty of Health: Medicine, Dentistry & Human Sciences<br>Snowsill , Tristan ; University of Exeter, College of Medicine and Health<br>Callaghan, Lynne; University of Plymouth, Faculty of Health: Medicine, Dentistry & Human Sciences<br>Price, Lisa; University of Exeter, Sport & Health Sciences<br>Horrell, Jane; University of Plymouth, Faculty of Health: Medicine, Dentistry & Human Sciences<br>King, Jennie; University of Plymouth, Faculty of Health: Medicine, Dentistry & Human Sciences<br>Gude, Alex; University of Plymouth, Faculty of Health: Medicine, Dentistry & Human Sciences

George, Mary; University of London St George's, Division of Population Health Sciences and Education
Wahlich, Charlotte; University of London St George's, Division of Population Health Sciences and Education
Hamilton, Louisa; University of Oxford Division of Public Health and Primary Health Care, Nuffield Department of Primary Care Health Sciences
Cheema, Kelisha; University of Nottingham, School of Medicine
Campbell, Sarah; University of Plymouth, Faculty of Health: Medicine, Dentistry & Human Sciences
Preece, Dan; Plymouth City Council, Public Dispensary

Keywords: PRIMARY CARE, PUBLIC HEALTH, Clinical trials < THERAPEUTICS

SCHOLARONE™
Manuscripts

I, the Submitting Author has the right to grant and does grant on behalf of all authors of the Work (as defined in the below author licence), an exclusive licence and/or a non-exclusive licence for contributions from authors who are: i) UK Crown employees; ii) where BMJ has agreed a CC-BY licence shall apply, and/or iii) in accordance with the terms applicable for US Federal Government officers or employees acting as part of their official duties; on a worldwide, perpetual, irrevocable, royalty-free basis to BMJ Publishing Group Ltd ("BMJ") its licensees and where the relevant Journal is co-owned by BMJ to the co-owners of the Journal, to publish the Work in this journal and any other BMJ products and to exploit all rights, as set out in our licence.

The Submitting Author accepts and understands that any supply made under these terms is made by BMJ to the Submitting Author unless you are acting as an employee on behalf of your employer or a postgraduate student of an affiliated institution which is paying any applicable article publishing charge ("APC") for Open Access articles. Where the Submitting Author wishes to make the Work available on an Open Access basis (and intends to pay the relevant APC), the terms of reuse of such Open Access shall be governed by a Creative Commons licence – details of these licences and which Creative Commons licence will apply to this Work are set out in our licence referred to above.

Other than as permitted in any relevant BMJ Author's Self Archiving Policies, I confirm this Work has not been accepted for publication elsewhere, is not being considered for publication elsewhere and does not duplicate material already published. I confirm all authors consent to publication of this Work and authorise the granting of this licence.

For peer review only

A randomised controlled trial of tailored support to increase physical activity and reduce smoking in smokers not immediately ready to quit: Protocol and baseline participant characteristics for the Trial of physical Activity assisted Reduction of Smoking (TARS) study.

Taylor, A.H.[1], Thompson, T. P.[1], Ussher, M.[2,3], Aveyard, P.[4], Murray, R. L.[5], Harris, T.[2], Creanor, S.[1,6], Green, C.[6], Streeter, A. [1], Chynoweth, J.[1], Ingram, W. [1], Greaves, C. J.[7], Hancocks, H. [1] (deceased), Snowsill, T.[6] , Callaghan, L.[1], Price, L. [8], Horrell, J.[1], King, J.[1], Gude, A.[1], George, M.[2], Wahlich, C. [2], Hamilton, L.[4], Cheema, K.[5], Campbell, S.[1], Preece, D.[9]

[1] Faculty of Health, University of Plymouth, Plymouth Science Park, Plymouth, PL6 8BX, UK
adrian.taylor@plymouth.ac.uk
tom.thompson@plymouth.ac.uk
siobhan.creanor@plymouth.ac.uk
adam.streeter@plymouth.ac.uk
jade.chynoweth@plymouth.ac.uk
wendy.ingram@plymouth.ac.uk
lynne.callaghan@plymouth.ac.uk
jane.horrell@plymouth.ac.uk
jennie.king@plymouth.ac.uk
alex.gude@plymouth.ac.uk
sarah.campbell@plymouth.ac.uk

[2] Population Health Research Institute, St. George's University of London, London, SW17 0RE, UK
mussher@sgul.ac.uk
tharris@sgul.ac.uk
mageorge@sgul.ac.uk
cwahlich@sgul.ac.uk

[3] Institute for Social Marketing and Health, University of Stirling, Stirling, FK9 4LA, UK

[4] Nuffield Department of Primary Care Health Sciences, University of Oxford, Oxford, OX2 6GG, UK
paul.aveyard@phc.ox.ac.uk
Louisa.Hamilton@warwick.ac.uk

[5] School of Medicine, University of Nottingham, Nottingham, NG7 2UH, UK
Rachael.Murray@nottingham.ac.uk
kelisha.cheema@hotmail.com

[6] University of Exeter Medical School, University of Exeter, Exeter, EX1 2LU, UK
C.Green@exeter.ac.uk
T.M.Snowsill@exeter.ac.uk

[7] School of Sport, Exercise and Rehabilitation Sciences, University of Birmingham, Birmingham, B15 2TT UK
C.J.Greaves@bham.ac.uk

[8] Sport & Health Sciences, University of Exeter, Exeter, EX1 2LU, UK
L.R.S.Price@exeter.ac.uk

[9] Plymouth City Council, Public Dispensary, Catherine Street, Plymouth, PL1 2AA
dan.preece@plymouth.gov.uk

Corresponding Author:
Professor Adrian Taylor, Faculty of Health, University of Plymouth, Plymouth Science Park, Plymouth, Devon, PL6 8BX.
Email: adrian.taylor@plymouth.ac.uk Tel: +44 7952400835

For peer review only - http://bmjopen.bmj.com/site/about/guidelines.xhtml

ABSTRACT

Introduction: Smoking reduction can lead to increased success in quitting. This study aims to determine if a client-focused motivational support package for smoking reduction (and quitting) and increasing (or otherwise using) physical activity (PA) can help smokers who do not wish to quit immediately to reduce the amount they smoke, and ultimately quit. This paper reports the study design, methods, and baseline sample characteristics.

Methods & analysis: A pragmatic, multi-centred, parallel, two group, randomised controlled superiority clinical trial, with embedded process evaluation and economics evaluation. Participants who wished to reduce smoking with no immediate plans to quit were randomised 1:1 to receive either (i) tailored individual health trainer face-to-face and/or telephone support to reduce smoking and increase PA as an aid to smoking reduction (intervention) or (ii) brief written/electronic advice to reduce or quit smoking (control). Participants in both arms of the trial were also signposted to usual local support for smoking reduction and quitting. The primary outcome measure is 6 month carbon monoxide confirmed floating prolonged abstinence following participant self-reported quitting on a mailed questionnaire at 3 and 9 month post-baseline. Participants confirmed as abstinent at 9 months will be followed up at 15 months.

Results: Of 915 randomised participants, 55.4% were female, 84.9% white ethnicity, with a mean (SD) age 49.8 (13.9) years. At baseline participants smoked a mean (SD) 18.0 (13.4) cigarettes per day and 5.1% and 9.8% reported using smoking cessation medication or vaping products, respectively. The participants self-reported a mean (SD) of 548.7 (653.5) minutes of weekly moderate and vigorous PA.

Ethics and dissemination: Approved by SW Bristol NHS Research Committee (17/SW/0223). Dissemination will include publication of findings for the stated outcomes, parallel process evaluation and economic evaluation in peer reviewed journals. Results will be disseminated to trial participants and health care providers.

Registration: ISRCTN47776579

For peer review only - http://bmjopen.bmj.com/site/about/guidelines.xhtml

Strengths and limitations of this study

- This is the first study to determine whether offering support to increase physical activity alongside smoking reduction is effective and cost-effective in increasing smoking abstinence among smokers not immediately ready to quit but who wish to reduce. The study involves over 900 participants recruited across four sites.
- The study's primary outcome is biochemically verified 6-month prolonged floating abstinence between 3 and 9 months post-baseline, with a secondary endpoint to consider differences at 15 months.
- The intervention involved considerable public involvement in both the pilot and current trial and was person-centred, theory based, manualised and delivered by eight health trainers who were trained to deliver behavioural support, and subsequently remotely supervised by phone or Skype.
- A mixed methods process evaluation will explore the fidelity of intervention design, delivery, receipt and enactment and explore if and how key components in our logic model operated.
- As an effectiveness trial, the intervention does not involve a supervised exercise programme and some participants entering the study may have been more interested in smoking reduction rather than increasing physical activity. This may limit the study's contribution to the exercise and smoking cessation evidence.

For peer review only

For peer review only - http://bmjopen.bmj.com/site/about/guidelines.xhtml

INTRODUCTION

Smoking remains the main cause of preventable morbidity and premature death in high-income nations. [1] The annual cost of smoking in England is estimated to be £11 billion to society, of which £2.5 billion is to the National Health Service (NHS) in England. [2] Tobacco control policies and individually targeted interventions have helped to reduce the UK population smoking prevalence rate to 14.7%, [3] but prevalence varies considerably by socio-economic and mental health status, contributing to growing health inequalities.

The UK's National Institute for Health and Care Excellence (NICE) Public Health No. 10 guidelines [4] for smoking cessation focus on identifying a quit date and abrupt cessation, with a recognition of the importance of motivational support to supplement pharmacological support. For those not intending to quit immediately, smoking reduction may lead to more quit attempts and subsequent successful abstinence, though there are limitations in the evidence [5-8] with a wide range of approaches to reduction (e.g. pharmacological, behavioural support, and self-initiated approaches). Motivational support appears to have the potential to support reduction in smoking, and the greater the reduction the greater the likelihood of successful quitting. [9]

The English Smoking Toolkit Study (2011 to 2014) suggests there is interest in smoking reduction, rather than immediately quitting, among 50% of smokers, and approximately 30% of UK smokers report using e-cigarettes to do so. [10] Other evidence based approaches to self-regulate smoking are needed as there remains uncertainty over the safety of e-cigarettes. [11] There is considerable interest in encouraging smokers to acutely and chronically use physical activity to manage smoking [12 13] though a recent systematic review revealed only 1 of 24 randomised controlled trials provided evidence that an exercise programme can aid abstinence for at least 6 months [14] among those attempting to quit. That said, most studies were of low quality and focused on proof of concept, rather than being pragmatic and offering an acceptable and feasible intervention for smokers more generally.

A unique randomised pilot study provided promising support for short-term effects of a behavioural support intervention offered by a health trainer for increasing physical activity and smoking reduction on cigarettes smoked and abstinence. [15 16] Previous studies [17] have reported that a self-determination based intervention to support autonomy and perceived competence for smokers can facilitate long-term smoking abstinence, and this approach was embedded in the pilot intervention. Intervention participants had an average 4.2 sessions by phone or face to face with the health trainer, with a range of 0-8 sessions. Compared with the control group, they were significantly more likely to achieve at least a 50% reduction in number of cigarettes smoked (39% vs 20%), to attempt to quit (22% vs 6%), and be abstinent up to 8 weeks after quit day (14% vs 4%), and at 16 weeks (10% vs 4%). A higher proportion of the intervention group also reported using physical activity for controlling smoking: 55% vs 22% and 37% vs 16%, at 8 and 16 weeks, respectively. [15] The intervention costs were approximately £192 per participant and exploratory cost-effectiveness modelling indicates that the intervention may be cost-effective. [15]

Physical activity is likely to influence smoking behaviour through both implicit and explicit processes. [12] A smoker could focus on becoming physically active which in turn has emotional benefits which may implicitly reduce cognitive and emotional triggers for smoking. Exercise may also be explicitly used to acutely manage cigarette cravings and withdrawal symptoms, [18 19] and chronically manage weight gain associated with smoking reduction or help in a shift towards a healthier identity.

In prospective population surveys and trials, weight gain and fear of weight gain is associated with reluctance to quit smoking and remain abstinent, especially among women and initially heavier

For peer review only - http://bmjopen.bmj.com/site/about/guidelines.xhtml

smokers,[20-22] with a meta-analysis study reporting an average of 4.67kg (95% confidence interval: 3.96 to 5.38) gained after 12 months of abstinence.[23] There is evidence that physical activity is effective for preventing long-term weight gain after smoking cessation,[24] not only by increased energy expenditure and metabolic rate, but also through self-regulation of energy intake, particularly emotional snacking.[25] Finally, as a result of increasing physical activity, a smoker may begin to establish a different identity (e.g. investing in personal fitness and generally becoming a "healthy person"), which in turn may trigger a desire to reduce harm from smoking through reduction and ultimately quitting.[15]

Following a successful pilot study[15] there is a need to establish the effectiveness and cost-effectiveness of behavioural support for increasing physical activity and reducing smoking on longer term abstinence among smokers not immediately ready to quit.

AIMS & OBJECTIVES

The overarching aim of the trial is to determine if an individually-tailored behavioural intervention for smokers wishing to reduce but not immediately quit provides an effective and cost-effective approach to supporting increases in physical activity and smoking reduction, resulting in increased rates of quit attempts and subsequent 6-month prolonged smoking abstinence.

The specific aims of the trial are to determine whether the intervention, compared with support as usual (SAU), will:

- Increase the proportion of participants who achieve 6 month prolonged biochemically-verified floating abstinence at 9 months post-baseline.
- Increase the proportion of participants who self-report a reduction in number of cigarettes smoked (between baseline and both 3 months and 9 months) of at least 50%.
- Increase the proportion of participants who achieve biochemically-verified prolonged abstinence at 15 months post-baseline (i.e., 12 months post-intervention).
- Increase self-reported physical activity at 3 and 9 months post-baseline, and accelerometer assessed physical activity at 3 months post-baseline.
- Improve quality of life, self-reported weight and cigarette cravings at 3 and 9 months post-baseline.

Further aims are:

- To conduct a health economic evaluation to estimate the costs of delivering the intervention and differences in health and social care costs between intervention and SAU at 9 months post-baseline. This will also estimate the cost-effectiveness of the intervention compared with SAU at (i) 9 months and (ii) over a longer term / lifetime horizon.
- To conduct an embedded mixed methods process evaluation to explore the mechanisms of action of the intervention and acceptability of study processes.

The current paper outlines the protocol for the TARS trial and presents the baseline data for the trial participants.

For peer review only - http://bmjopen.bmj.com/site/about/guidelines.xhtml

For peer review only

METHODS & ANALYSIS

This protocol is reported in accordance with the SPIRIT guidance [26] for protocols of clinical trials and the TIDieR guidelines [27] for intervention description.

Study design & setting

The TARS trial is a pragmatic, multi-centred, parallel, two group, randomised controlled superiority clinical trial to compare (i) tailored support to reduce smoking and increase physical activity (PA) as an aid to smoking reduction (intervention) with (ii) brief advice to reduce or quit smoking (control). The study includes a mixed methods embedded process evaluation and health economics evaluation. Recruitment to the trial was over 16 months (January 2018 – May 2019) in the geographical areas in and around four cities in the UK: Plymouth, Nottingham, London, and Oxford.

Study population

Potential participants were approached from primary and secondary care establishments and also from the community. Participants were adult smokers (≥ 18 years) who smoked ≥ 10 cigarettes per day (for at least 1 year), wished to reduce smoking but not quit immediately. We accepted smokers who were also using other nicotine containing or other cigarette management products. Smokers were ineligible if they were unable to engage in at least 15 minutes of moderate intensity PA, had any illness or injury that might be exacerbated by exercise, were unable to engage in the study and/or the intervention due to language or for other reasons (e.g. provide an unacceptable level of risk to the health trainer or researcher). All ineligible smokers were referred for smoking cessation advice in line with local usual practice.

Study procedures

Participant identification, approach and consent: A broad range of participant identification and approach methods were employed in an attempt to reach many different demographic groups and make the study as inclusive as possible. The most common approach method was through a search of a primary care practice patient database, with invitations sent to patients who were listed as smokers offering them the chance to get in touch with the trial team. In the early phase of recruitment, a study within a trial (SWAT) was conducted at GP practices at one research site, to compare the efficiency and cost-effectiveness of three different invitation methods (full information pack, single page invite, text message). [28] Other approaches were via secondary health care and stop smoking services (for those who had failed to quit), and by social media, pharmacies, dental practices, community organisations and local businesses using posters and leaflets.

As shown in Figure 1 (participant flow chart), smokers expressing interest in the trial were screened for eligibility by researchers at each site. If suitable and still interested in joining the study, participants provided evidence of consent either at a face-to-face meeting or via telephone.

Baseline assessment: Participants then completed a baseline questionnaire with a member of the local study team either face-to-face, by mail, or over the telephone. The schedule of measures and data collection at baseline and follow-up are shown in Table 1.

For peer review only - http://bmjopen.bmj.com/site/about/guidelines.xhtml

Table 1. Schedule of baseline and follow-up measures

	Screening & Baseline	Month 3	Month 9	Month 15 #
Demographics (e.g., age, gender, education attained, employment status)	X			
Self-reported cigarettes per day (or equivalent)	X	X	X	X
Reduction of ≥ 50 % in number of cigarettes smoked since baseline *		X	X	
Biochemically confirmed abstinence (self-reported quitters only)		X	X	X
Self-reported floating prolonged abstinence (since quitting smoking, with quit date, if relevant) over at least 6 months *			X	X
Accelerometer assessed minutes of moderate & vigorous physical activity in a sub-sample		X		
Self-reported 7-day recall of physical activity	X	X	X	
Heaviness of Smoking Index	X			
Use of smoking management products	X	X	X	X
Urge & strength of urge to smoke	X	X		
Engagement with the health trainer intervention (8 weeks, plus optional 6 weeks additional support if a quit attempt is made)		X		
Health & social care utilisation	X	X	X	
Health related quality of life (EQ-5D-5L & SF12)	X	X	X	
Self-reported weight & height (to calculate BMI)	X	X	X	
Self-reported process measures:<br>Importance and confidence in smoking reduction and cessation<br>Importance and confidence in being physically active<br>Action planning to change smoking<br>Action planning to change physical activity<br>Self-monitoring of smoking<br>Self-monitoring of physical activity<br>Availability of support to reduce smoking<br>Availability of support to increase physical activity<br>Use of physical activity for smoking regulation	X	X		
Serious adverse events (self-reported)		X	X	
Qualitative process evaluation (in parallel throughout) (sample)	X	X	X	X

Only participants with biochemically verified abstinence at 9 months are followed-up at 15 months post-baseline

* Derived measure

For peer review only - http://bmjopen.bmj.com/site/about/guidelines.xhtml

Randomisation: Participants were individually randomised to either the intervention or control group (1:1 ratio) following completion of baseline assessments, to ensure concealment was preserved. Randomisation was achieved by means of a 24-hour web-based system created by the UKCRC-registered Peninsula Clinical Trials Unit (CTU) in conjunction with a statistician independent from the trial team, and used random permuted blocks, with stratification for recruitment site and a dichotomised low/high score derived from the heaviness of smoking index (HSI). [29] The HSI score is calculated from summing responses to two questions:

i. when the first cigarette is smoked after waking, scored as >60 minutes (score 0); 31-60 minutes (1); 6-30 minutes (2); and within 5 minutes (3)
ii. how many cigarettes are smoked in a typical day, scored as ≤10 cigarettes (0); 11-20 cigarettes (1); 21-30 cigarettes (2); > 30 cigarettes (3)

Total HSI scores of 0-4 were categorised as low, and 5-6 were considered high for the purposes of stratification.

Following randomisation, all participants were sent a letter from the co-ordinating CTU confirming which trial arm they had been assigned to, and a guidance sheet on usual support locally for smoking reduction and cessation. The participant's GP was also sent a letter notifying them that one of their patients is participating in the study.

Blinding: It was not possible to blind participants to their allocated group. Every effort was made to ensure that the trial team remained blind to the allocation of each participant when collecting follow-up data (including researchers collecting carbon monoxide (CO) measurements), but this was not always possible. Health trainers delivering the intervention were obviously aware of the participant's allocation to trial arm, but were discouraged from communicating with site researchers about this. Questionnaire booklets and accelerometers were mailed out from and returned to the CTU without knowledge of trial arm allocation.

Follow-up: At 3 and 9 months post-baseline all participants are posted a questionnaire booklet and a freepost envelope to return the completed booklet to the CTU. A £20 shopping voucher is mailed to participants upon CTU receipt of the completed booklets at both 3 and 9 months. To increase response rates, motivational postcards are mailed to participants before the follow-up questionnaires are sent out. Up to two reminder letters are issued (and a further three telephone calls as required) to remind participants to return the questionnaire booklets, and the option of the participant telephoning a member of the research team to aid completion of the booklets is offered. Participants are given the option to just complete the key questions about smoking behaviour if the questionnaire booklet is not returned to the CTU within 2 weeks and to submit these responses by e-mail, phone or text if preferred to maximise follow-up data on key outcomes. If participants do not complete the key questions (regardless of method) within a 4 week window they are categorised as not completing follow-up at that time point.

All participants who report having made a quit attempt in the questionnaire booklet and not smoked since that date at 3 and 9 months are contacted for biochemical verification of abstinence.

At 3 months post-baseline, approximately 20% of participants were sent wrist-worn waterproof accelerometer (GeneActive Original accelerometer®, Activinsights, Kimbolton, UK, http://www.geneactiv.org/) with instructions to wear constantly for one whole week (day and night), and a freepost return envelope to be sent to the CTU. To maximise data completeness, participants scheduled to receive an accelerometer were sent a standardised letter from the CTU, two weeks before

For peer review only - http://bmjopen.bmj.com/site/about/guidelines.xhtml

receiving the accelerometer, advising them that they would shortly be receiving the device, and asking them to inform the CTU if they were unable to wear it. A letter was sent to participants who did not object to wearing the device, 3 days into the 10 day recording period prompting participants to start wearing the device if they had not already done so. Up to two reminder letters and a follow-up phone call were made to participants if they did not return the accelerometer.

Trial allocation groups

Intervention: The TARS intervention was based on the intervention developed for the EARS pilot trial, with further refinement based on feedback from the EARS process evaluation. [15] Throughout the development of the intervention for the EARS and TARS studies, we engaged with smokers from a wide range of socio-economic backgrounds to ensure an acceptable person-centred approach was embedded.

Throughout the pilot trial and before commencing the definitive trial, we conducted individual and focus group discussions with smoking-cessation practitioners, researchers, public health consultants, community workers (including volunteers) and people who currently or previously smoked. We reviewed literature on using exercise as an aid to quitting, and consulted with academic experts on behaviour change for physical activity, smoking reduction and smoking cessation. These activities informed the intervention principles and theoretical basis, structure and delivery.

The intervention aimed to be empowering and put the client at the centre of the decision making process. All aspects were designed to promote self-determined behaviour, focussed on elements of self-determination theory which emphasises people's sense of autonomy, competence, and relatedness. [30-32] This was in part achieved by adopting motivational interviewing principles [33] as the guiding delivery style for the practitioners which have been proposed to enhance and promote self-determined behaviour. [34]

An intervention delivery model, or "roadmap", was developed between the pilot and definitive trial (Figure 2) to aid in the conceptualising of the intervention process and support the manualisation of the intervention and training of the practitioners. This was supported by the development of a set of "core competencies" (Table 2) developed from the pilot trial which outlined key processes, components, and behaviour change techniques that the health trainers were expected to deliver and form the basis of fidelity assessment. [35] A comprehensive training manual was developed, outlining all the skills, behaviour change techniques and strategies to support behaviour change intended for use in the TARS study. This was used as the basis for a 3 day training course, which was delivered by TT, AT, CG, and LC. Health trainers then engaged with a wide range of "practice participants" to complete their training. The health trainers attended regular (bi-weekly for the first 3 months and monthly thereafter) two-hour formative feedback supervision teleconferences throughout the study period to help to embed skills and to benefit from each other's shared experiences. Individual supervision for the health trainers was available as needed, provided by the intervention lead (TT).

For peer review only - http://bmjopen.bmj.com/site/about/guidelines.xhtml

Table 2 Intervention components, aims, content and indicative change in processes

Intervention components	Aim	Content	Indicative change in processes
Active participant involvement (1)	Develop rapport, build trust, and shared respect.	Effective communication skills. Build autonomous support.	Participant feedback on health trainer-led support.
Build motivation to reduce smoking (2) and increase physical activity (3)	Identify ambivalence towards reduction & quitting. Build self-awareness & confidence to cut down and increase physical activity.	Help smoker to identify importance & challenges of reduction & cessation, and implicit & explicit roles of physical activity (motivational interviewing techniques).	Smoker has desire and confidence to cut down and perhaps quit over the early sessions, and increase physical activity. Smoker engages in more self-monitor of smoking and physical activity behaviour.
Self-monitor smoking and physical activity and set goals to reduce smoking (4) and increase physical activity (5)	Develop strategies to reduce smoking and increase physical activity.	Set SMART goals to reduce smoking and increase physical activity. Signpost to physical activity opportunities & remove barriers to do physical activity.	Goals identified and action plans developed. Smoker engages in more goal setting to reduce smoking and increase physical activity behaviour.
Review/problem solving for smoking (6) & physical activity (7)	Build confidence, perceptions of control, & self-regulation skills.	Smoker reflects on smoking reduction and physical activity, identifies barriers and possible solutions, increases and sets new targets; perhaps to quit.	Goals revised to reflect confidence to increase physical activity, reduce smoking, and possibly quit.
Integrating idea of changing smoking and physical activity (8)	To help smoker to identify any links between smoking and physical activity	Explore with smoker how physical activity may influence smoking (and vice versa) (person centred exchange of information (Ask-Tell-Discuss)).	Smoker increases use of physical activity as an aid to smoking reduction.
Reinforce health identity shift (9)	To help identify shift from smoker to healthier identity.	Smoker reflects on label as heavy – moderate – light or non-smoker status, and more active person.	Decrease in importance of smoking and increase in importance of doing physical activity identified.
Manage social influences on smoking (10) and physical activity (11)	To involve others in process of reducing smoking and increasing physical activity. Manage negative or undermining social influences.	Smoker identifies key others who can support reduced smoking (or cessation) and increasing physical activity, and engages with them in preferred ways. Uses negotiation and discussion to manage negative social influences.	Support from others identified as important and used for smoking reduction or cessation, and increasing physical activity.

For peer review only - http://bmjopen.bmj.com/site/about/guidelines.xhtml

Participants allocated to the intervention arm were offered individually tailored behavioural support from a health trainer. The health trainer delivered the processes outlined in Figure 2 and Table 2, with the option of up to 8 weekly sessions, and a further 6 sessions if the participant wanted support after quitting, and aimed to empower participants to decide what support was offered, and where, when and for how long. Signposting to local smoking cessation support services was also offered to those wishing to quit. If a smoker wished to reduce smoking using e-cigarettes or licenced nicotine containing products (LNCP) they were also offered any local available support for this.

Health trainers were appointed on the basis of having good communication skills, including empathy, and at least some training and experience in supporting health behaviour change. All health trainers had at least a first degree in a related field although this was not a pre-requisite for the role. The health trainer was trained to support change in both smoking and physical activity and help individuals to make the connections between the two. As described in Table 2, the core intervention processes that the health trainer was trained to deliver were: (1) building rapport and supporting autonomous behaviour change; (2) building motivation; (3) supporting self-monitoring and goal setting; (4) problem solving; (5) integrating smoking and PA behaviour; (6) supporting a health identity shift; (7) supporting the management of social influence on behaviours.

The support offered was broadly structured (based on delivering the processes outlined in Table 2 and Figure 2) but health trainers were trained to be flexible in their approach, to tailor it for individual needs and preferences. They were trained to do this using a person-centred approach and principles from motivational interviewing such as showing empathy and reflective listening. [36] They were also trained to assess (and be aware of) participants' needs in relation to the core psychological needs posited by Self-Determination Theory, [37] which we referred to in the training as "the three Cs": Control (having choice /autonomy in decision-making around behaviour change); Competence (developing self-efficacy /building confidence in the ability to change) and Connectedness (social acceptance of the new behaviour /support from important others for making changes).

A fuller intervention description, including the trainer manual and intervention materials will be published as supplementary materials linked to the final NIHR project report.

Support as usual (SAU): Participants allocated to both arms of the trial received written guidance for smoking reduction and cessation, including web links to what is offered at local level, or paper versions of this information. Typically, there are no formal programmes for use of medication to support reduction (rather than abrupt stopping) and people usually buy their own replacement therapy or e-cigarette product.

Determination of sample size

Since the planned primary analysis is a comparison of the proportions of the binary primary outcome, the sample size calculation was based on a two-sided Fisher exact test. An abstinence rate of 5% for the control group and detectable effect of 6% (i.e. an increase from 5% to 11% due to the intervention) are conservative estimates consistent with those from the EARS pilot study [15] and those reported from a systematic review of pharmacological interventions. [38] The corresponding odds ratio for this effect size is 2.35. Participants with missing outcome data will be assumed to be still smoking following the Russell Standard, [39] and the numbers of participants in each allocated group are assumed to be in the ratio of 1:1. Under these conditions, according to Stata v14.2, the minimum number of participants required to detect an abstinence rate of 11% compared with that of 5% in the control group, with a significance level of no more than 5% and power of at least 90%, is 900, above which a power in excess of 90% is maintained.

For peer review only - http://bmjopen.bmj.com/site/about/guidelines.xhtml

Outcome measures

Table 1 lists the outcomes measures and when they are being assessed.

Primary outcome measure: Biochemically verified 6 month floating prolonged abstinence between 3 and 9 months. [40] Abstinence will be confirmed by expired CO <10ppm measured with a CareFusion MicroCO meter (Williams Medical Supplies, Rhymney, UK, www.carefusion.co.uk) at a face-to-face visit.

Participants who self-reported abstinence at 3 months and who were confirmed as abstinent through biochemical verification via expired CO at a face-to-face assessment and then self-report abstinence (and not having smoked even a puff since the 3 month assessment) at 9 months, again confirmed by expired CO, will be defined as having prolonged abstinence over at least 6 months.

Other smoking-related measures: Only participants who have biochemically verified abstinence at 9 months are being contacted at 15 months post-baseline to assess floating prolonged abstinence over a period of 12 months (3 to 15 months). Participants who were not abstinent at 3 months but have biochemically confirmed abstinence at 9 months will be contacted at 15 months to confirm floating prolonged abstinence of 6 months between 9 and 15 months. As a contingency measure for verification of abstinence during the coronavirus (COVID-19) outbreak, abstinence will be confirmed by saliva cotinine level <12ng/mL[41] using a mailed self-collection kit and assay provided by ABS Laboratories (York, UK, www.acmgloballab.com). This contingency measure will apply to follow-up for secondary outcomes for a minority of participants. While the intervention is expected to primarily influence quitting in the first 3 months of the study, it is possible that a sustained quit attempt occurs after the 3 month assessment as a result of the health trainer building behaviour change skills which are used subsequently to reduce and then quit smoking.

Participants are asked to self-report the number of cigarettes smoked and type of nicotine product (i.e. pipes, cigars and roll your own). We estimated 0.45 grams of tobacco was the equivalent of one cigarette based on a previous rigorous study [42] and reported in the EARS pilot study. [15] From this we will estimate the number of cigarettes smoked at follow up, and also calculate if participants reduce smoking by at least 50% between (i) baseline and 3 months and (ii) baseline and 9 months.

PA measures: The 7-day recall measure of PA [43] is used to assess self-reported weekly minutes of moderate and vigorous PA. An objective measure of total weekly minutes of moderate and vigorous physical activity (MVPA) was collected with a wrist-worn waterproof GENEActiv accelerometer [44] around the 3 month follow-up time-point. Participants were asked to wear the accelerometer on the wrist of the non-dominant hand constantly for one week and then return to the CTU. This accelerometer shows the wearer no information about their PA levels and does not have obvious motivational value.

Physical measures: Participants are asked to self-report their height and weight from which body mass index will be calculated.

Health related quality of life measures: The EQ-5D-5L (EuroQol Group, 1990) comprises the following five dimensions: mobility, self-care, usual activities, pain/discomfort and anxiety/depression. Each dimension has five levels: no problems, slight problems, moderate problems, severe problems, and extreme problems. [45] The SF-12 is a 12-item, patient-reported survey of patient health, consisting of twelve questions. [46]

Resource use / health care service use: Use of primary and community-based health and social services, and hospital-based in-patient and out-patient services are captured using a resource use questionnaire developed in two pilot trials involving health trainer led interventions. [16,47] It sought to capture the number of contacts that occurred (if any) with a range of health and social care professionals and where

For peer review only - http://bmjopen.bmj.com/site/about/guidelines.xhtml

those contacts took place since completing the previous survey, using both fixed and open format responses. Reasons for hospital admissions were also requested.

Self-reported process measures: Single survey items, using a 5-point Likert scale (Strongly disagree to Strongly agree) assessed the psychological and behavioural processes that the intervention was designed to influence as shown in Table 2. These were used in other trials. [15 48 49] A single survey item, using a 6-point scale (Not at all to All the time), was used to assess frequency of urge to smoke in the past week. [50] A single survey item (6-point Likert scale, no urges to extremely strong) assessed strength of urges to smoking in the past week [50] as used previously. [15]

Economic evaluation

The economic evaluation will (i) estimate the long-term cost-effectiveness of the TARS intervention plus SAU versus SAU alone, over a life time horizon using a model-based economic evaluation, and (ii) estimate the cost-effectiveness of the TARS intervention plus SAU versus SAU alone over the primary 9-month trial follow up, in an economic evaluation conducted alongside the trial. The longer-term model-based economic analysis is considered the primary economic analysis, consistent with the approach commonly applied in the context of cost-effectiveness analysis in smoking cessation settings.

The primary perspective of the economic analyses will be that of the NHS and Personal Social Services (i.e. Third Party Payer), with a broader perspective explored in sensitivity analyses. The primary economic endpoint will be the quality-adjusted life-year (QALY, using EQ-5D data), with results presented as incremental cost effectiveness ratios (ICERs) representing estimated costs per QALY gained.

The economic analysis will be undertaken against a pre-defined health economics analysis plan (HEAP), which is available on request. In summary, the trial-based cost-effectiveness analysis will use participant level data collected within-trial to estimate (i) the resource use and costs associated with the delivery of the TARS intervention, (ii) broader resource use and costs associated with health and care service use by group, (iii) QALYs by group, and (iv) the incremental cost per unit of outcome (e.g. cost per incremental QALY, cost per quitter) over the 9-month follow-up. In this analysis, EQ-5D-5L data will be used to estimate QALYS, deriving health state values at each time point, using the published tariff values for England (presently recommendations are for values to be derived using methods reported by Van Hout et al [51]), and using the area under the curve approach.[52] Analyses will be based on an ITT principle, using a complete case analysis, and will assess uncertainty, and include detailed sensitivity analyses.

The model-based economic evaluation will adopt a longer term perspective (lifetime) beyond the trial follow-up, to present a policy relevant cost effectiveness analyses, that predicts future costs and QALYs after the trial endpoint based on the reported effectiveness of the TARS intervention. A decision analytic model will be used, with the model based on the model developed and described in the prior pilot study (EARS), [15] which we will update and adapt using a review of the recent literature on modelling in this area and based on input from a Stakeholder Group. The model-based evaluation will be based on good practice guidelines for decision analytic modelling in the HTA context. [53-55]

Embedded mixed methods process evaluation

A mixed methods process evaluation will focus on trial processes and methods and will attempt to understand the effective components and processes of the intervention. During the internal pilot phase, the evaluation will focus on barriers and facilitators for recruitment methods, initial intervention engagement, and early intervention implementation. For the subsequent main trial, the evaluation will

For peer review only - http://bmjopen.bmj.com/site/about/guidelines.xhtml

focus on acceptability of study processes (via a qualitative sub study), intervention engagement levels, predictors of intervention engagement, intervention delivery fidelity, and evaluating the implementation of the intervention process model (participant understanding of the intervention model (receipt fidelity), mediating effects of process measures on PA and smoking outcomes (enactment fidelity), mediating effects of PA on smoking outcomes, approaches and acceptability of smoking reduction, multiple behaviour change, progression to cessation, and other perceived effectual intervention components).

Data for the process evaluation will be collected via: the trial database, audio recordings of intervention sessions, and audio recorded and transcribed interviews with trial participants, research assistants, health trainers, and GPs/practice managers.

Trial data handling

Data are collected and maintained in accordance with the current legal and regulatory requirements. A data management protocol has been produced by the CTU to ensure secure data collection and storage in accordance with the Data Protection Act 1998, and later conforming to the General Data Protection Regulation 2016 and Data Protection Act 2018.

Electronic study records will be held over the lifetime of the study in secure storage solutions aligned with the host institution's information security classification policy. At the time of writing, electronic study data are stored in a SQL server database on a restricted access, secure server maintained by the University of Plymouth. Data are entered into the database via a bespoke web-based data entry system encrypted using SSL version 3 (QuoVadis Global, http://www.quovadisglobal.com). Access to identifiable information is restricted and permission-based.

A parallel, linked, bespoke data system has been used to manage intervention engagement post-randomisation. The system captures all health trainer attempted and actual contact with intervention participants in real time to produce summary data (e.g., number of sessions, duration and mode of sessions, notes on session content) and aid supervision sessions, and intervention management (e.g., should a health trainer be unavailable).

Identifiable information will be omitted from the transcriptions of the process evaluation interviews.

Statistical analysis plan

A detailed statistical analysis plan was drafted during the trial delivery phase and will be approved by an independent statistician and wider Trial Steering Committee, prior to database lock. The analyses will be reported in full and in accordance with the Consolidated Standards of Reporting Trials (CONSORT) guidelines. [56] The main planned analyses are summarised below.

Baseline characteristics and summary statistics

Descriptive statistics by allocated group will be presented for the baseline, and primary and secondary outcomes, which includes the smoking outcomes and questionnaire data as well as the smoking and

For peer review only - http://bmjopen.bmj.com/site/about/guidelines.xhtml

For peer review only

physical mediators, with the exception of the primary outcome and secondary abstinence measures (assessed at 3, 9 and 15 months only) and accelerometer outcomes (assessed only at 3 months).

For continuous outcomes, summary information will be presented in the form of means alongside standard deviations (SD). Count and skewed continuous data will be presented in terms of median, and inter-quartile range. For categorical outcomes, summary information will be presented in the form of frequencies and percentages.

Inferential statistical comparison at baseline of randomised groups is not good practice [57] and it is expected that participants in both groups will, on average, be similar. Following initial primary analysis, if substantial imbalance at baseline is identified in any key variables, such as gender and age, the importance of any imbalance will be noted and additional adjusted analyses may be performed.

Primary analysis

The null hypothesis is that there is no difference in CO verified 6-month prolonged floating-abstinence rates between the intervention and control groups at 9 months post-baseline. In line with the Russell Standard schedule, [39] the primary comparative analysis will be conducted on an intention-to-treat basis, in which participants with missing responses will be considered to still be smokers. Interpretation of the primary effectiveness analysis will be based on the odds ratio from the logistic regression model adjusted for (fixed effect) stratification variables: site as a factor and HSI as an ordinal covariate. Both the adjusted (primary analysis) and unadjusted odds ratios and corresponding 95% confidence intervals will be presented. Primary effectiveness shall also be presented as a relative risk along with the absolute between-group differences in abstinence rates, as recommended in the CONSORT guidelines for parallel group randomised trials. [58]

Planned sensitivity analysis of the primary outcome:

(a) Rather than assuming participants with missing responses at 3 or 9 months were still smoking, the primary outcome will be imputed under a number of varying assumptions and the primary analysis re-run for each of the scenarios;
(b) A complier average causal effect (CACE) analysis will be undertaken, if greater than 20% of participants allocated to the intervention group are categorised as not having completed at least two interventions sessions with a health trainer, with individual participants in the intervention group categorised as compliers if they completed at least two intervention sessions. Participants in the control group and non-compliers in the intervention group will be compared to compliers in the intervention group.

Secondary analyses

To explore whether the primary outcome was influenced by the intervention dose actually received (i.e. number of health trainer sessions attended) the primary outcome shall be modelled on the number of health trainer sessions attended in the intervention group only, adjusting for the stratification variables. Although the trial is not powered to detect the influence of moderating factors on the primary outcome, secondary analyses will be undertaken to explore whether the intervention effect is modified by key demographic and/or behavioural factors at baseline. These are pre-specified as the postcode-based index of multiple deprivation (IMD); the factor indicating smoking cessation medication or a vaping product at baseline; MVPA level, confidence to quit; and the stratification variable, HSI. The multi-variable logistic regression model outlined above will be extended to include the interaction term of

For peer review only - http://bmjopen.bmj.com/site/about/guidelines.xhtml

allocated group and each of the listed potential modifying variables. Evidence of an interaction shall be interpreted through the 95% confidence intervals of the coefficient for the interaction term.

During the development of this study, the potential health trainer effect was considered at length. Given the lack of evidence on individual health trainer effects, the study design and sample size calculations do not allow for such partial clustering within health trainers (within recruitment sites). However, an exploratory analysis of the intervention effect will be undertaken using a multi-level, mixed-modelling approach, to allow for the partially nested data: participants allocated to the intervention group will be partially clustered within the health trainer, in turn nested within sites.

Analysis of secondary outcomes**:** Between-group comparisons will be undertaken, including for the following key secondary outcomes:

- Biochemically verified point prevalence abstinence at 3, 9 and 15* months post-baseline
- Self-reported point prevalence abstinence at 3, 9 and 15* months post-baseline
- Prolonged biochemically verified abstinence over 6 months between 9 and 15 months post-baseline
- Prolonged biochemically verified abstinence for at least 12 months between 3 and 15 months post-baseline (derived from biochemically confirmed abstinence at all three follow-up time points)
- At least a 50% reduction in reported smoking levels between i) baseline and 3 months and ii) baseline and 9 months
- Number of cigarettes used on an average day over the past week (incl. equivalent cigars, tobacco) at 3, 9 and 15* months post-baseline
- Total number of LNCP used on an average day over the past week at 3, 9 and 15* months post-baseline
- Total number of self-reported minutes engaged in MVPA over the past week at 3 and 9 months post-baseline
- BMI at 3 and 9 months post-baseline

NB: * only for those with biochemically confirmed abstinence at 9 months.

The following data is derived from accelerometers mailed to a sub-group of participants in both arms of the trial along with the 3 month questionnaire, which are returned after the accelerometers have been worn for seven days:

- The average time spent in moderate to vigorous activity over the past week
- The average daily time spent sleeping over the past week

No adjustment for multiple analyses will be made; such adjustment methods are too conservative when outcomes are positively correlated, as they would be in this trial. Analyses will use multi-variable linear regression (continuous outcomes) or logistic regression (binary outcomes) to compare each of these secondary outcomes between allocated groups, with adjustment for site as a factor, and HSI, as an ordinal variable, as well as baseline values of the outcome as appropriate. As accelerometer data were only available at 3 months, only summary statistics for weekly minutes of MVPA, shall be presented by allocated group without adjustment for baseline variables.

The between-group comparisons of continuous outcomes will be reported as mean differences together with 95% confidence intervals, unless the outcomes are substantially skewed. Both adjusted and unadjusted differences will be presented. The between-group comparisons of binary outcomes will be

For peer review only - http://bmjopen.bmj.com/site/about/guidelines.xhtml

reported as the adjusted and unadjusted odds ratios with conversion to relative risks and corresponding confidence intervals, along with the absolute between-group differences in abstinence rates.

Analyses will be undertaken to investigate whether any effect of the intervention in terms of reduction in smoking at 3 months and 9 months is modified by key sociodemographic and/or behavioural factors at baseline. These pre-specified factors at baseline are using smoking management medication or vaping, IMD, the stratification variables, MVPA level, and confidence to quit. The multi-variable models will be extended to include the interaction term of allocated group and each of the potential modifying variables.

Model checking: The logistic regression model for the primary analysis is pre-specified. However, observations identified as potential outliers through their influence on the model, may be excluded as part of a sensitivity analysis. The distributional assumptions of multi-variable linear regression models will be visually assessed through plots of residuals. If there are concerns about distributional assumptions being met, bootstrapped confidence intervals for the adjusted between-group differences will be produced.

Mediational analysis

If there is evidence that the intervention is effective, an exploratory mediational analysis will be undertaken to determine if any effect of allocated group on the primary outcome was mediated by changes in smoking and/or physical activity between baseline and 3 month follow-up, adjusted for the stratification variables (site and HSI). Further analyses will explore if changes in smoking and physical activity are mediated by changes in outcomes from the questionnaire on attitudes to smoking between baseline and 3 months (importance and confidence to reduce smoking/increase physical activity; self-monitoring and goal setting; urges to smoke).

Baseline Descriptive data

The baseline descriptive data (pooled across allocated groups to maintain blinding of the trial team) are shown in Table 3. Of 915 randomised participants, 55.4% were female, 84.9% were of white ethnicity, and they had a mean (SD) age 49.8 (13.9) years. 72% were recruited through primary care either by invitation letter or opportunistically.

At baseline participants smoked a mean (SD) 18.0 (13.4) cigarettes per day, and 5.1% and 9.8% reported using smoking cessation medication and used vaping devices, respectively. At baseline, 18.5% of participants were defined as heavy smokers (i.e. an HSI score of 5-6). The participants self-reported a mean (SD) of 548.7 (653.5) weekly minutes of MVPA.

For peer review only

For peer review only - http://bmjopen.bmj.com/site/about/guidelines.xhtml

Table 3 Baseline characteristics for the whole sample

		Both groups
N		915
Gender - N female (%)		507 (55.4)
Age – N, mean (SD)		915, 49.8 (13.9)
BMI – N, mean (SD)		892, 26.4 (5.9)
Ethnicity – N (%)	White	777 (84.9)
	South Asian	17 (1.0)
	Black Caribbean, African or other	67 (7.3)
	Other	53 (5.8)
Relationship status - N (%)	Single (never married or civil partnership)	390 (42.6)
	Widowed, divorced, or dissolved or surviving civil partnership	141 (14.5)
	Married, or living together as if married, or civil partnership	391 (42.8)
Index of multiple deprivation rank (derived from postcode) – N, mean (SD)		913, 14430.4 (8734.8)
Education level attained – N (%)	No qualifications	197 (21.5)
	GCSEs	515 (56.3)
	A-level	241 (26.3)
	First degree or above	262 (28.6)
Employment status – N (%)	Full or part-time paid	418 (45.7)
	Work as volunteer	26 (2.8)
	Full-time education	35 (3.8)
	Looking after the home	45 (4.9)
	Retired	146 (16.0)
	Unemployed	156 (17.0)
	Other	89 (9.7)
Site - N (%)	Plymouth	247 (27)
	London	285 (31)
	Nottingham	166 (18)

For peer review only - http://bmjopen.bmj.com/site/about/guidelines.xhtml

		Both groups
	Oxford	217 (24)
Recruitment pathway – N (%)	GP invitation	568 (62.1)
	Primary care opportunistic invitation	91 (9.9)
	Community advert, media, stop smoking services or word of mouth	229 (25.0)
	Other	27 (3.0)
First cigarette smoked after waking – N (%)	> 60 minutes	91 (9.9)
	31 - 60 minutes	114 (12.5)
	6 - 30 minutes	412 (45.0)
	Within 5 minutes	298 (32.6)
Cigarettes smoked a day – N (%)	10 or less	120 (13.1)
	11-20	543 (59.3)
	21-30	189 (20.7)
	> 30	63 (6.9)
Number of cigarettes per day smoked – mean (SD)		18.0 (13.4)
Heaviness of Smoking Index – mean (SD)		3.2 (1.4)
LNCP use over the last week – N (%)	Smoking cessation medication	47 (5.1)
	Vaping	90 (9.8)
Partner smokes – N (%)		549 (66.5)
Weekly self-reported MVPA minutes – N, mean (SD)		915, 548.7 (653.5)

For peer review only

Discussion

The manuscript describes the methods involved in the trial design and intervention delivered, and the pooled descriptive baseline data for the final sample recruited. The sample overall were moderately heavy smokers, who were generally physically active (based on self-reported data), with few participants using smoking cessation medication or vaping devices. Further information on the effectiveness of recruitment methods, and lessons learnt from different approaches will be presented in a NIHR Final Report and other publications, along with the trial findings.

Patient & public involvement

The TARS research team has worked with people who smoke, not as research participants, individually and in groups from across all communities, to guide research questions, study design and conduct, intervention development and dissemination over the past 15 years. As an example of their input, they had varying views on the merits of vaping to reduce smoking and how various forms of PA may help. A university employee and non-employee PPI group (of people who currently or have previously smoked) regularly met to input into intervention and trial methods development. There was further PPI input into project management group meetings and trial steering committee meetings throughout the trial. The study team has also engaged with key stakeholders involved in commissioning and delivering community interventions to assess where the proposed intervention would best fit and the study team will continue to do this prior to and during intervention development.

Trial monitoring & oversight

The trial Project Management Group includes a multidisciplinary team of clinicians and researchers with expertise in all aspects of trial design, intervention development and delivery, conduct, analysis and quality assurance. The Trial Steering Committee (TSC) involves independent expertise to help guide the trial on behalf of the funders and provide oversee trial progress. The TSC will also sign-off the Statistical and Health Economic Analyses Plans. The Data Monitoring Committee (DMC) provides independent expertise and support particularly regarding evidence or reason why the trial should be amended or terminated based on recruitment rates, compliance, safety or efficacy.

Ethics & dissemination

The study has been approved by the South West – Central Bristol Research Ethics Committee (REC reference: 17/SW/0223) and the Health Research Authority. A number of approvals have been granted for minor and substantial amendments; the amendment history and full details of the amendments are available upon request.

The research team will work with stakeholders and PPI representatives at each site, and nationally, to help to interpret the results and the implications for policy and practice. The PMG will establish a publication plan and authorship rules. Reporting will follow current CONSORT guidelines for randomised trials. The study results will be submitted for publication in relevant international, high impact, peer reviewed journals. Names of key collaborators and groups who have contributed to the trial will be clearly stated in all publications. The study findings will be presented at regional, national and international meetings as appropriate.

Safety considerations

The recording and reporting of non-serious adverse events (AEs) in this low-risk study is not required. Serious Adverse Events (SAEs) will be documented from the time of participant consent until a maximum of 8 weeks after the follow-up assessment at 9 months. A protocol for identifying, reporting

For peer review only - http://bmjopen.bmj.com/site/about/guidelines.xhtml

and managing SAEs has been established by the CTU, in conjunction with the Project Management Group, DMC and TSC, and conforms to the requirements of the trial Sponsor and NHS REC.

Access to data

The CTU Data Manager is the custodian of the final trial data set, with the exception of the audio files and transcripts arising from qualitative interviews with participants which are held by the process evaluation team.

Access will be granted to the Sponsor and host institution for the purposes of study-related monitoring, audits and inspections.

Members of the study team will have restricted access to the anonymised dataset for the purposes of conducting the trial, and to undertake the statistical and economic data analysis and the process evaluation.

Data requests should be submitted to the corresponding author for consideration. Following publication of the primary results of the trial, access to available anonymised data may be granted depending on review of the data request and appropriate agreements being in place.

Current study status

The TARS study completed participant recruitment in May 2019. Data collection for the 9 and 15 month follow-up assessments are expected to be completed in April and October 2020, respectively, and results are expected to be published in mid 2021.

List of abbreviations

BMI	body mass index
CACE	complier average causal effect
CO	carbon monoxide
CTU	Peninsula Clinical Trials Unit
DMC	Data Monitoring Committee
EARS	A pilot randomised trial to assess the methods and procedures for evaluating the clinical effectiveness and cost-effectiveness of Exercise Assisted Reduction then Stop (EARS) among disadvantaged smokers
EQ-5D-5L	EuroQo -5 dimension – 5 level
HSI	Heaviness of Smoking Index
ICER	incremental cost effectiveness ratio
IMD	Index of Multiple Deprivation
LNCP	licenced nicotine containing products
MVPA	moderate and vigorous physical activity
NICE	National Institute for Health and Care Excellence
PPI	Patient and Public Involvement
QALY	Quality Adjusted Life Years
SAE	Serious Adverse Event
SAU	support as usual
SF12	12-Item Short Form Health Survey version 2
SQL	Structured Query Language
SSL	Secure Sockets Layer

For peer review only - http://bmjopen.bmj.com/site/about/guidelines.xhtml

TARS Trial of physical Activity assisted Reduction of Smoking
TSC Trial Steering Committee
UKCRC UK Clinical Research Collaboration

Contributors

AHT conceived the idea for the study with TT, MU, PA, CG, S.Cr and CJG.
AHT, TT, MU, PA, CG, SCr, CJG and HH contributed to the final study design and development of the protocol.
AHT, TT and CJG developed the intervention, with LC who supported PPI input.
DP provided a public health perspective on usual community Stop Smoking Service support.
AHT, PA, MU, TH and RLM were the Principal Investigators at participating sites.
JK, AG, MG, CW, LH and KC (Research Assistants) led recruitment, baseline data collection, and face to face carbon monoxide verified abstinence assessments at participating sites.
LP was consulted on accelerometer data capture and processed the accelerometer data.
SCr, AS and JC provided the statistical analysis plan.
AS and JC performed the descriptive analysis of baseline demographic data.
CG developed the health economics analysis plan with TS.
TT developed the process evaluation plan with CJG, LC and JH.
HH, WI and SCa were the PenCTU trial managers.

Disclosure of potential competing interests

AG, AS, AHT, CJG, CG, CW, DP, HH, JC, JK, LC, LH, MG, MU, PA, RLM, SCr, SCa, TH, TS, TT and WI report a grant from NIHR (NIHR HTA award ref 15/111/01) during the conduct of the study.

PA is an NIHR Senior Investigator and is part funded by NIHR Oxford Biomedical Research Centre and Applied Research Centre.

SCr reports grants from NIHR HTA during the conduct of the study, and various other grants from NIHR and UK charities outside the submitted work. She is also Interim Co-Director (and previously Director) of the UKCRC-Registered Peninsula Clinical Trials Unit, which is in receipt of NIHR Clinical Trials Unit Support Funding (current award ends 31 August 2021).

LP reports consultancy fees from NIHR during the conduct of the study; grants from Living Streets Charity, personal fees from NIHR, personal fees from NIHR PHR, personal fees from NIHR PHR rapid response, grants from Wellcome Trust seed corn (internal funding) outside the submitted work; and discloses that the physical activity group in Sport and Health Sciences at the University of Exeter has a collaboration with Activinsights (the manufacturer of the physical activity monitor), to provide study design advice and data analysis, but that analysis of the physical activity data in the present study was not undertaken as part of that service.

JH has nothing to disclose.

All authors critically revised successive drafts of the manuscript and approved the final version.

For peer review only - http://bmjopen.bmj.com/site/about/guidelines.xhtml

Neither the study sponsor nor funder has a direct role in study design; collection, management, analysis, and interpretation of data; writing of the report; manuscript writing or dissemination of results. The funder will be informed of impending research output for this study.

Acknowledgements

This research has been conducted independently by researchers at the Universities of Plymouth, Oxford, Nottingham, and St George's London, with the support of Clinical Research Networks at each site and PenARC.

We appreciate the support of the study Sponsor, University Hospitals Plymouth NHS Trust.

The study was led by researchers and managed by the Peninsula Clinical Trials Unit (PenCTU) at the University of Plymouth. We are indebted to Dr Helen Hancocks for her contribution to the TARS study as PenCTU Clinical Trial Manager; sadly Helen passed away suddenly on 24th January 2019.

We thank Doug Webb (PenCTU Assistant Trial Manager) for helping to develop the protocol, and acknowledge Jonny Wilks (PenCTU Data Manager) for TARS data management.

Our thanks go to all organisations involved in recruitment, and especially those working in primary and secondary care and in local Stop Smoking Services. We thank James Davies and Will Moyle for preparing the search code for identifying potential participants within primary care.

We acknowledge the contribution of the health trainers in delivering the intervention: Alix Covenant, Stephanie Erivo, Gemma Fox, Alex Gude, Sarah Kennedy, Amanda Perry, Lucy Porter and Charlotte Wahlich.

We gratefully acknowledge the members of our Trial Steering Committee (TSC) and Data Monitoring Committee (DMC) for their valuable support and guidance. TSC members: Prof. Marcus Munafo (Chair), Dr Stephanie MacNeill, Prof. Amanda Amos and Dr Michael Callaghan (PPI representative). Sadly, Michael died having made a valuable contribution to the study. DMC members: Dr Rebecca Playle (Chair), Dr Charlie Foster and Dr Jamie Brown.

Our sincerest thanks go to all the participants involved in the trial.

Funding

This study is funded by the National Institute for Health Research (NIHR) HTA programme (project reference 15/111/01). The views expressed are those of the authors and not necessarily those of the NIHR or the Department of Health and Social Care.

Provenance and peer review

The NIHR (HTA no 15/111) identified the research question 'What is the clinical and cost-effectiveness of additional physical activity in tapered smoking reduction programmes?' as a priority in 2015. The current study was favourably reviewed by independent experts as an outline bid to the NIHR (HTA) in 2015, and as a full bid in 2016.

Data sharing statement This is an Open Access article distributed in accordance with the Creative Commons Attribution Non Commercial (CC BY-NC 3.0) licence, which permits others to distribute, remix, adapt, build on this work non-commercially, and licence their derivative works on different terms, provided the original work is properly cited and the use is non-commercial. http://creativecommons.org/licenses/by-nc/3.0/.

For peer review only - http://bmjopen.bmj.com/site/about/guidelines.xhtml

REFERENCES

1. GBD 2015 Risk Factors Collaborators. Global, regional, and national comparative risk assessment of 79 behavioural, environmental and occupational, and metabolic risks or clusters of risks, 1990-2015: a systematic analysis for the Global Burden of Disease Study 2015. *Lancet* 2016;388:1659-724.
2. Department of Health. Towards a Smokefree Generation: A Tobacco Control Plan for England London: Department of Health; 2017 [updated 13 January 2020. Available from: https://www.gov.uk/government/publications/towards-a-smoke-free-generation-tobacco-control-plan-for-england2020.
3. Office for National Statistics. Adult smoking habits in the UK 2018 [Available from: https://www.ons.gov.uk/peoplepopulationandcommunity/ healthandsocialcare/healthandlifeexpectancies/bulletins/ adultsmokinghabitsingreatbritain/2018.
4. NICE. NICE Public Health Guidance 10, Smoking Cessation Services. London, 2008.
5. Klemperer EM, Hughes JR, Naud S. Reduction in Cigarettes per Day Prospectively Predicts Making a Quit Attempt: A Fine-Grained Secondary Analysis of a Natural History Study. *Nicotine Tob Res* 2019;21(5):648-54. doi: 10.1093/ntr/nty056 [published Online First: 2018/03/27]
6. Lindson N, Klemperer E, Hong B, et al. Smoking reduction interventions for smoking cessation. *Cochrane Database Syst Rev* 2019;9:Cd013183. doi: 10.1002/14651858.CD013183.pub2 [published Online First: 2019/10/01]
7. Lindson-Hawley N, Hartmann-Boyce J, Fanshawe TR, et al. Interventions to reduce harm from continued tobacco use. *Cochrane Database Syst Rev* 2016;10:Cd005231. doi: 10.1002/14651858.CD005231.pub3 [published Online First: 2016/11/02]
8. NICE. Tobacco: harm-reduction approaches to smoking. NICE Public Health Guidance 45. London: NICE, 2013.
9. Klemperer E, Hughes J. Does the Magnitude of Reduction in Cigarettes Per Day Predict Smoking Cessation? A Qualitative Review. *Nicotine Tob Res* 2016;18(1):88-92. doi: doi: 10.1093/ntr/ntv058. Epub 2015 Mar 5.
10. West R, Mohr G, Proudfoot H, et al. Top-line findings on smoking in England from the Smoking Toolkit Study: UCL, 2019.
11. Gotts JE, Jordt SE, McConnell R, et al. What are the respiratory effects of e-cigarettes? *BMJ,* 2019;366:l5275. doi: 10.1136/bmj.l5275 [published Online First: 2019/10/02]
12. Taylor A, Thompson TP. Exercise in the prevention, treatment and management of substance use addictions. In: M W, H B, eds. Exercise and Mental Health: Neurobiological Mechanisms of the Exercise Effect on Depression, Anxiety, and Well-being. New York: Taylor and Francis 2018.
13. Tobacco Use and Dependence Guideline Panel. Treating Tobacco Use and Dependence: 2008 Update,. In: US Surgeon Gen Report, ed. Rockville (MD), 2008.
14. Ussher MH, Faulkner GEJ, Angus K, et al. Exercise interventions for smoking cessation. *Cochrane Database Syst Rev* 2019;2019(10) doi: 10.1002/14651858.CD002295.pub6 [published Online First: 2019/11/07]
15. Taylor AH, Thompson TP, Greaves CJ, et al. A pilot randomised trial to assess the methods and procedures for evaluating the clinical effectiveness and cost-effectiveness of Exercise Assisted Reduction then Stop (EARS) among disadvantaged smokers. *Health Technol Assess* 2014;18(4):1-324. doi: 10.3310/hta18040 [published Online First: 2014/01/18]
16. Thompson TP, Greaves CJ, Ayres R, et al. An Exploratory Analysis of the Smoking and Physical Activity Outcomes From a Pilot Randomized Controlled Trial of an Exercise Assisted Reduction to Stop Smoking Intervention in Disadvantaged Groups. *Nicotine Tob Res* 2016;18(3):289-97. doi: 10.1093/ntr/ntv099 [published Online First: 2015/05/15]
17. Williams G, Niemiec C, Patrick H, et al. The importance of supporting autonomy and perceived competence in facilitating long-term tobacco abstinence. *Ann Behav Med* 2009;37(3):315-24.

For peer review only - http://bmjopen.bmj.com/site/about/guidelines.xhtml

18. Haasova M, Warren FC, Ussher M, et al. The acute effects of physical activity on cigarette cravings: systematic review and meta-analysis with individual participant data. *Addiction* 2013;108(1):26-37. doi: 10.1111/j.1360-0443.2012.04034.x [published Online First: 2012/08/07]
19. Haasova M, Warren FC, Ussher M, et al. The acute effects of physical activity on cigarette cravings: exploration of potential moderators, mediators and physical activity attributes using individual participant data (IPD) meta-analyses. *Psychopharmacology (Berl)* 2014;231(7):1267-75. doi: 10.1007/s00213-014-3450-4 [published Online First: 2014/02/14]
20. Pisinger C, Jorgensen T. Waist circumference and weight following smoking cessation in a general population: the Inter99 study. *Prev Med* 2007;44(4):290-5. doi: 10.1016/j.ypmed.2006.11.015 [published Online First: 2007/01/16]
21. Filozof C, Fernandez Pinilla MC, Fernandez-Cruz A. Smoking cessation and weight gain. *Obes Rev* 2004;5(2):95-103. doi: 10.1111/j.1467-789X.2004.00131.x [published Online First: 2004/04/17]
22. Meyers AW, Klesges RC, Winders SE, et al. Are weight concerns predictive of smoking cessation? A prospective analysis. *J Consult Clin Psychol* 1997;65(3):448-52. [published Online First: 1997/06/01]
23. Aubin H, Farley A, Lycett D, et al. Weight gain in smokers after quitting cigarettes: meta-analysis. *Bmj* 2012;345(e4439) doi: doi: https://doi.org/10.1136/bmj.e4439
24. Parsons AC, Shraim M, Inglis J, et al. Interventions for preventing weight gain after smoking cessation. *Cochrane Database Syst Rev* 2009(1):Cd006219. doi: 10.1002/14651858.CD006219.pub2 [published Online First: 2009/01/23]
25. Oh H, Taylor AH. Self-regulating smoking and snacking through physical activity. *Health Psychol* 2014;33(4):349-59. doi: 10.1037/a0032423 [published Online First: 2013/05/15]
26. Chan AW, Tetzlaff JM, Altman DG, et al. SPIRIT 2013 statement: defining standard protocol items for clinical trials. *Ann Intern Med* 2013;158(3):200-7. doi: 10.7326/0003-4819-158-3-201302050-00583 [published Online First: 2013/01/09]
27. Hoffmann TC, Glasziou PP, Boutron I, et al. Better reporting of interventions: template for intervention description and replication (TIDieR) checklist and guide. *BMJ* 2014;348:g1687. doi: 10.1136/bmj.g1687 [published Online First: 2014/03/13]
28. Hancocks H, King J, Gude A, et al. The effect of the method of invitation on recruitment of participants from GP practices to a trial of a smoking reduction intervention: a study within a trial. International Clinical Trials Methodology Conference. Brighton, UK, 2019:P-143 https://az659834.vo.msecnd.net/eventsairwesteuprod/production-inconference-public/fe8d0a1d39e7403da5745e4b9fab416e.
29. Heatherton TF, Kozlowski LT, Frecker RC, et al. Measuring the heaviness of smoking: using self-reported time to the first cigarette of the day and number of cigarettes smoked per day. *Br J Addict* 1989;84(7):791-9. [published Online First: 1989/07/01]
30. Deci EL, Ryan RM. Intrinsic motivation and self-determination in human behaviour. New York: Plenum. 1985.
31. Deci E, Ryan R. The what and why of human goal pursuits: Human needs and the self-determination of behavior. . *Psychological Inquiry* 2000;11:227–68
32. Miller W, Rollnick S. Meeting in the middle: motivational interviewing and self-determination theory. *Int J Behav Nutr Phys Act* 2012;9(25) doi: 1479-5868-9-25 [pii] 10.1186/1479-5868-9-25
33. Miller W. Motivational Interviewing with problem drinkers. *Behavioural Psychotherapy,* 1983;11:147–72.
34. Markland D, Ryan R, Tobin V, et al. Motivational Interviewing and Self Determination Theory. *Journal Social and Clinical Psychology* 2005;24:811–31.
35. Thompson TP, Lambert J, Greaves C, et al. Intervention delivery fidelity assessment of a counselling based intervention for multiple behaviours. *Health Psychology* 2018;37(7):627-37.
36. Miller W, Rollnick S. Motivational interviewing: helping people change. New York: Guilford press 2012.
37. Deci EL, and Ryan RM. Handbook of self-determination research. Rochester, New York: University of Rochester Press 2002.

For peer review only - http://bmjopen.bmj.com/site/about/guidelines.xhtml

38. Cahill K, Stevens S, Perera R, et al. Pharmacological interventions for smoking cessation: an overview and network meta-analysis. *Cochrane Database Syst Rev* 2013(5):Cd009329. doi: 10.1002/14651858.CD009329.pub2 [published Online First: 2013/06/04]
39. West R, Hajek P, Stead L, et al. Outcome criteria in smoking cessation trials: proposal for a common standard. *Addiction* 2005;100:299-303.
40. Aveyard P, Wang D, Connock M, et al. Assessing the outcomes of prolonged cessation-induction and aid-to-cessation trials: floating prolonged abstinence. *Nicotine Tob Res* 2009;11(5) doi: doi: 10.1093/ntr/ntp035 [published Online First: 2009/04/07]
41. Jarvis MJ, Fidler J, Mindell J, et al. Assessing smoking status in children, adolescents and adults: cotinine cut-points revisited. *Addiction* 2008;103(9):1553-61. doi: 10.1111/j.1360-0443.2008.02297.x [published Online First: 2008/09/12]
42. Laugesen M, Epton M, Frampton C, et al. Hand-rolled cigarette smoking patterns compared with factory-made cigarette smoking in New Zealand men. *BMC Public Health* 2009;9(194)
43. Blair SN, Haskell WL, Ho P, et al. Assessment of habitual physical activity by a seven-day recall in a community survey and controlled experiments. *Am J Epidemiol* 1985;122(5):794-804. [published Online First: 1985/11/01]
44. Powell C, Carson BP, Dowd KP, et al. Simultaneous validation of five activity monitors for use in adult populations. *Scand J Med Sci Sports* 2017;27(12):1881-92. doi: 10.1111/sms.12813 [published Online First: 2016/12/03]
45. Herdman M, Gudex C, Lloyd A, et al. Development and preliminary testing of the new five-level version of EQ-5D (EQ-5D-5L). *Qual Life Res* 2011;20(10):1727-36. doi: 10.1007/s11136-011-9903-x [published Online First: 2011/04/12]
46. Ware J, Jr., Kosinski M, Keller SD. A 12-Item Short-Form Health Survey: construction of scales and preliminary tests of reliability and validity. *Med Care* 1996;34(3):220-33. [published Online First: 1996/03/01]
47. Callaghan L, Thompson TP, Creanor S, et al. Public Health Research. Individual health trainers to support health and well-being for people under community supervision in the criminal justice system: the STRENGTHEN pilot RCT. Southampton (UK): NIHR Journals Library 2019.
48. Ingram W, Webb D, Taylor RS, et al. Multicentred randomised controlled trial of an augmented exercise referral scheme using web-based behavioural support in individuals with metabolic, musculoskeletal and mental health conditions: protocol for the e-coachER trial. *BMJ Open* 2018;8(9):e022382. doi: 10.1136/bmjopen-2018-022382 [published Online First: 2018/09/24]
49. Thompson TP, Callaghan L, Hazeldine E, et al. A Health Trainer led motivational intervention plus usual care for people under community supervision compared to usual care alone: a study protocol for a parallel group pilot randomised controlled trial (STRENGTHEN). *Trials* 2018;4(8(6))::e023123.
50. Taggar JS, Lewis S, Docherty G, et al. Do cravings predict smoking cessation in smokers calling a national quit line: secondary analyses from a randomised trial for the utility of 'urges to smoke' measures. *Subst Abuse Treat Prev Policy* 2015;10:15. doi: 10.1186/s13011-015-0011-8 [published Online First: 2015/04/18]
51. van Hout B, Janssen MF, Feng YS, et al. Interim scoring for the EQ-5D-5L: mapping the EQ-5D-5L to EQ-5D-3L value sets. *Value Health* 2012;15(5):708-15. doi: 10.1016/j.jval.2012.02.008 [published Online First: 2012/08/08]
52. Brazier J, Ratcliffe J, Salomon J, et al. Measuring and Valuing Health Benefits for Economic Evaluation. Oxford: Oxford University Press 2007.
53. Kaltenthaler E, Tappenden P, Paisley S, et al. NICE DSU Technical Support Document 13: Identifying and reviewing evidence to inform the conceptualisation and population of cost-effectiveness models 2011 [Available from: nicedsu.org.uk.
54. Caro J, Briggs A, Siebert U, et al. Modeling Good Research Practices—Overview: A report of the ISPOR-SMDM Modeling Good Research Practices Task Force-1. *Value in Health* 2012;15:796-803.
55. Philips Z, Ginnelly L, Sculpher M, et al. Review of guidelines for good practice in decision-analytic modelling in health technology assessment. *Health Technology Assessment* 2004;8(36)

For peer review only - http://bmjopen.bmj.com/site/about/guidelines.xhtml

56. Schulz KF, Altman DG, Moher D, et al. CONSORT 2010 statement: updated guidelines for reporting parallel group randomised trials. *BMJ* 2010;340:c332. doi: 10.1136/bmj.c332 [published Online First: 2010/03/25]
57. Assmann SF, Pocock SJ, Enos LE, et al. Subgroup analysis and other (mis)uses of baseline data in clinical trials. *Lancet* 2000;355(9209):1064-9. doi: 10.1016/s0140-6736(00)02039-0 [published Online First: 2000/04/01]
58. Schulz KF, Altman DG, Moher D. CONSORT 2010 statement: updated guidelines for reporting parallel group randomized trials. *Ann Intern Med* 2010;152(11):726-32. doi: 10.7326/0003-4819-152-11-201006010-00232 [published Online First: 2010/03/26]

For peer review only

For peer review only - http://bmjopen.bmj.com/site/about/guidelines.xhtml

Figure 1 Participant flow chart

Figure 2 Indicative map of the TARS intervention components

For peer review only

For peer review only - http://bmjopen.bmj.com/site/about/guidelines.xhtml

Figure 1 Participant flow chart

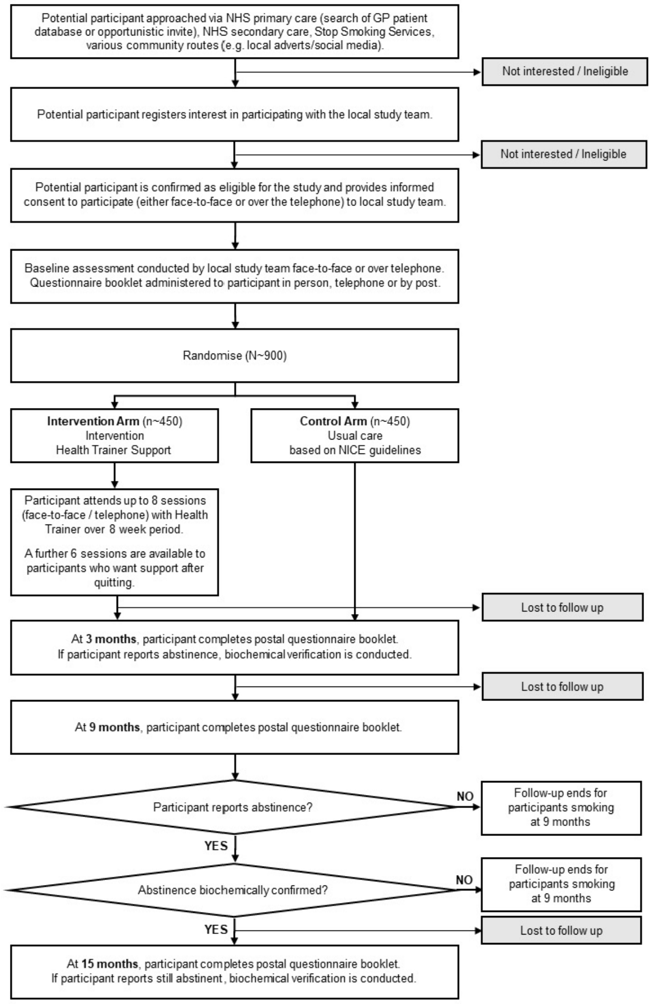

Caption : Figure 1 Participant flow chart

210x297mm (114 x 114 DPI)

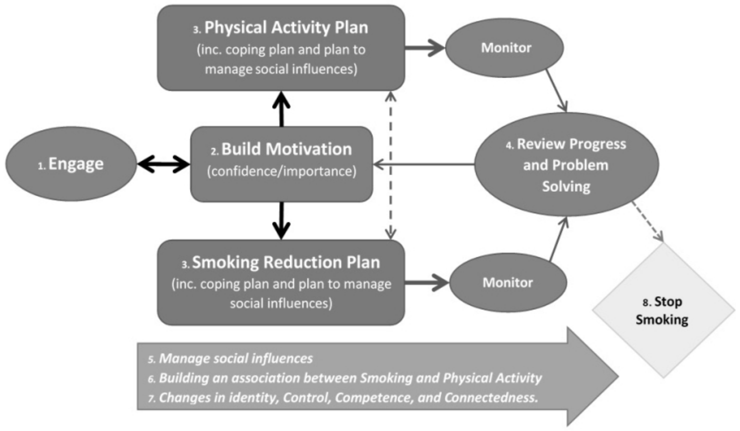

Figure 2 Indicative map of the TARS intervention components

215x279mm (150 x 150 DPI)

STANDARD PROTOCOL ITEMS: RECOMMENDATIONS FOR INTERVENTIONAL TRIALS

SPIRIT 2013 Checklist: Recommended items to address in a clinical trial protocol and related documents*

Section/item	Item No	Description	Addressed on page number
**Administrative information**			
Title	1	Descriptive title identifying the study design, population, interventions, and, if applicable, trial acronym	1______________
Trial registration	2a	Trial identifier and registry name. If not yet registered, name of intended registry	1______________
	2b	All items from the World Health Organization Trial Registration Data Set	-
Protocol version	3	Date and version identifier	1_ V9.1 dated 11 June 2020
Funding	4	Sources and types of financial, material, and other support	6______________
Roles and responsibilities	5a	Names, affiliations, and roles of protocol contributors	6-7____________
	5b	Name and contact information for the trial sponsor	1, 6
	5c	Role of study sponsor and funders, if any, in study design; collection, management, analysis, and interpretation of data; writing of the report; and the decision to submit the report for publication, including whether they will have ultimate authority over any of these activities	37, 41, 52
	5d	Composition, roles, and responsibilities of the coordinating centre, steering committee, endpoint adjudication committee, data management team, and other individuals or groups overseeing the trial, if applicable (see Item 21a for data monitoring committee)	51 (oversight committees)

For peer review only

For peer review only - http://bmjopen.bmj.com/site/about/guidelines.xhtml

**Introduction**			
Background and rationale	6a	Description of research question and justification for undertaking the trial, including summary of relevant studies (published and unpublished) examining benefits and harms for each intervention	11-15
	6b	Explanation for choice of comparators	28
Objectives	7	Specific objectives or hypotheses	15-16
Trial design	8	Description of trial design including type of trial (eg, parallel group, crossover, factorial, single group), allocation ratio, and framework (eg, superiority, equivalence, noninferiority, exploratory)	16
**Methods: Participants, interventions, and outcomes**			
Study setting	9	Description of study settings (eg, community clinic, academic hospital) and list of countries where data will be collected. Reference to where list of study sites can be obtained	17, 20
Eligibility criteria	10	Inclusion and exclusion criteria for participants. If applicable, eligibility criteria for study centres and individuals who will perform the interventions (eg, surgeons, psychotherapists)	22
Interventions	11a	Interventions for each group with sufficient detail to allow replication, including how and when they will be administered	27-28
	11b	Criteria for discontinuing or modifying allocated interventions for a given trial participant (eg, drug dose change in response to harms, participant request, or improving/worsening disease)	27
	11c	Strategies to improve adherence to intervention protocols, and any procedures for monitoring adherence (eg, drug tablet return, laboratory tests)	
	11d	Relevant concomitant care and interventions that are permitted or prohibited during the trial	27-28
Outcomes	12	Primary, secondary, and other outcomes, including the specific measurement variable (eg, systolic blood pressure), analysis metric (eg, change from baseline, final value, time to event), method of aggregation (eg, median, proportion), and time point for each outcome. Explanation of the clinical relevance of chosen efficacy and harm outcomes is strongly recommended	9, 18-20, 32
Participant timeline	13	Time schedule of enrolment, interventions (including any run-ins and washouts), assessments, and visits for participants. A schematic diagram is highly recommended (see Figure)	21, 32

For peer review only - http://bmjopen.bmj.com/site/about/guidelines.xhtml

Sample size	14	Estimated number of participants needed to achieve study objectives and how it was determined, including clinical and statistical assumptions supporting any sample size calculations	41
Recruitment	15	Strategies for achieving adequate participant enrolment to reach target sample size	22-23
**Methods: Assignment of interventions (for controlled trials)**			
Allocation:			
Sequence generation	16a	Method of generating the allocation sequence (eg, computer-generated random numbers), and list of any factors for stratification. To reduce predictability of a random sequence, details of any planned restriction (eg, blocking) should be provided in a separate document that is unavailable to those who enrol participants or assign interventions	25
Allocation concealment mechanism	16b	Mechanism of implementing the allocation sequence (eg, central telephone; sequentially numbered, opaque, sealed envelopes), describing any steps to conceal the sequence until interventions are assigned	25
Implementation	16c	Who will generate the allocation sequence, who will enrol participants, and who will assign participants to interventions	25
Blinding (masking)	17a	Who will be blinded after assignment to interventions (eg, trial participants, care providers, outcome assessors, data analysts), and how	26
	17b	If blinded, circumstances under which unblinding is permissible, and procedure for revealing a participant's allocated intervention during the trial	N/A
**Methods: Data collection, management, and analysis**			
Data collection methods	18a	Plans for assessment and collection of outcome, baseline, and other trial data, including any related processes to promote data quality (eg, duplicate measurements, training of assessors) and a description of study instruments (eg, questionnaires, laboratory tests) along with their reliability and validity, if known. Reference to where data collection forms can be found, if not in the protocol	28-30, 32, 38-39
	18b	Plans to promote participant retention and complete follow-up, including list of any outcome data to be collected for participants who discontinue or deviate from intervention protocols	28-30

For peer review only - http://bmjopen.bmj.com/site/about/guidelines.xhtml

Data management	19	Plans for data entry, coding, security, and storage, including any related processes to promote data quality (eg, double data entry; range checks for data values). Reference to where details of data management procedures can be found, if not in the protocol	38-39
Statistical methods	20a	Statistical methods for analysing primary and secondary outcomes. Reference to where other details of the statistical analysis plan can be found, if not in the protocol	41-43
	20b	Methods for any additional analyses (eg, subgroup and adjusted analyses)	42-43
	20c	Definition of analysis population relating to protocol non-adherence (eg, as randomised analysis), and any statistical methods to handle missing data (eg, multiple imputation)	42-43
**Methods: Monitoring**			
Data monitoring	21a	Composition of data monitoring committee (DMC); summary of its role and reporting structure; statement of whether it is independent from the sponsor and competing interests; and reference to where further details about its charter can be found, if not in the protocol. Alternatively, an explanation of why a DMC is not needed	51
	21b	Description of any interim analyses and stopping guidelines, including who will have access to these interim results and make the final decision to terminate the trial	33
Harms	22	Plans for collecting, assessing, reporting, and managing solicited and spontaneously reported adverse events and other unintended effects of trial interventions or trial conduct	36-37
Auditing	23	Frequency and procedures for auditing trial conduct, if any, and whether the process will be independent from investigators and the sponsor	38
**Ethics and dissemination**			
Research ethics approval	24	Plans for seeking research ethics committee/institutional review board (REC/IRB) approval	52
Protocol amendments	25	Plans for communicating important protocol modifications (eg, changes to eligibility criteria, outcomes, analyses) to relevant parties (eg, investigators, REC/IRBs, trial participants, trial registries, journals, regulators)	52

Consent or assent	26a	Who will obtain informed consent or assent from potential trial participants or authorised surrogates, and how (see Item 32)	23-24
	26b	Additional consent provisions for collection and use of participant data and biological specimens in ancillary studies, if applicable	N/A
Confidentiality	27	How personal information about potential and enrolled participants will be collected, shared, and maintained in order to protect confidentiality before, during, and after the trial	40
Declaration of interests	28	Financial and other competing interests for principal investigators for the overall trial and each study site	-
Access to data	29	Statement of who will have access to the final trial dataset, and disclosure of contractual agreements that limit such access for investigators	40-41
Ancillary and post-trial care	30	Provisions, if any, for ancillary and post-trial care, and for compensation to those who suffer harm from trial participation	53
Dissemination policy	31a	Plans for investigators and sponsor to communicate trial results to participants, healthcare professionals, the public, and other relevant groups (eg, via publication, reporting in results databases, or other data sharing arrangements), including any publication restrictions	53
	31b	Authorship eligibility guidelines and any intended use of professional writers	53
	31c	Plans, if any, for granting public access to the full protocol, participant-level dataset, and statistical code	-
**Appendices**			
Informed consent materials	32	Model consent form and other related documentation given to participants and authorised surrogates	-
Biological specimens	33	Plans for collection, laboratory evaluation, and storage of biological specimens for genetic or molecular analysis in the current trial and for future use in ancillary studies, if applicable	N/A

*It is strongly recommended that this checklist be read in conjunction with the SPIRIT 2013 Explanation & Elaboration for important clarification on the items. Amendments to the protocol should be tracked and dated. The SPIRIT checklist is copyrighted by the SPIRIT Group under the Creative Commons "Attribution-NonCommercial-NoDerivs 3.0 Unported" license.

BMJ Open

A randomised controlled trial of tailored support to increase physical activity and reduce smoking in smokers not immediately ready to quit: Protocol for the Trial of physical Activity assisted Reduction of Smoking (TARS) study.

Journal:	*BMJ Open*
Manuscript ID	bmjopen-2020-043331.R1
Article Type:	Protocol
Date Submitted by the Author:	28-Sep-2020

Complete List of Authors:	Taylor, Adrian; University of Plymouth, Faculty of Health: Medicine, Dentistry & Human Sciences<br>Thompson, T; University of Plymouth, Faculty of Health: Medicine, Dentistry & Human Sciences<br>Ussher, Michael; University of London St George's, Division of Population Health Sciences and Education; University of Stirling, Institute for Social Marketing<br>Aveyard, Paul; University of Oxford Division of Public Health and Primary Health Care, Nuffield Department of Primary Care Health Sciences<br>Murray, Rachael; University of Nottingham Faculty of Medicine and Health Sciences, School of Medicine<br>Harris, Tess; University of London St George's, Division of Population Health Sciences and Education<br>Creanor, Siobhan; University of Plymouth, Faculty of Health: Medicine, Dentistry & Human Sciences<br>Green, Colin; University of Exeter, College of Medicine and Health<br>Streeter, Adam; University of Plymouth, Faculty of Health: Medicine, Dentistry & Human Sciences<br>Chynoweth, Jade; University of Plymouth, Faculty of Health: Medicine, Dentistry & Human Sciences<br>Ingram, Wendy; University of Plymouth, Faculty of Health: Medicine, Dentistry & Human Sciences<br>Greaves, Colin; University of Birmingham School of Sport Exercise and Rehabilitation Sciences, School of Sport, Exercise and Rehabilitation Science<br>Hancocks, Helen; University of Plymouth, Faculty of Health: Medicine, Dentistry & Human Sciences<br>Snowsill , Tristan ; University of Exeter, College of Medicine and Health<br>Callaghan, Lynne; University of Plymouth, Faculty of Health: Medicine, Dentistry & Human Sciences<br>Price, Lisa; University of Exeter, Sport & Health Sciences<br>Horrell, Jane; University of Plymouth, Faculty of Health: Medicine, Dentistry & Human Sciences<br>King, Jennie; University of Plymouth, Faculty of Health: Medicine, Dentistry & Human Sciences<br>Gude, Alex; University of Plymouth, Faculty of Health: Medicine, Dentistry & Human Sciences<br>George, Mary; University of London St George's, Division of Population

	Health Sciences and Education<br>Wahlich, Charlotte; University of London St George's, Division of Population Health Sciences and Education<br>Hamilton, Louisa; University of Oxford Division of Public Health and Primary Health Care, Nuffield Department of Primary Care Health Sciences<br>Cheema, Kelisha; University of Nottingham, School of Medicine<br>Campbell, Sarah; University of Plymouth, Faculty of Health: Medicine, Dentistry & Human Sciences<br>Preece, Dan; Plymouth City Council, Public Dispensary
**Primary Subject Heading**:	Smoking and tobacco
Secondary Subject Heading:	Public health, Sports and exercise medicine
Keywords:	PRIMARY CARE, PUBLIC HEALTH, Clinical trials < THERAPEUTICS

I, the Submitting Author has the right to grant and does grant on behalf of all authors of the Work (as defined in the below author licence), an exclusive licence and/or a non-exclusive licence for contributions from authors who are: i) UK Crown employees; ii) where BMJ has agreed a CC-BY licence shall apply, and/or iii) in accordance with the terms applicable for US Federal Government officers or employees acting as part of their official duties; on a worldwide, perpetual, irrevocable, royalty-free basis to BMJ Publishing Group Ltd ("BMJ") its licensees and where the relevant Journal is co-owned by BMJ to the co-owners of the Journal, to publish the Work in this journal and any other BMJ products and to exploit all rights, as set out in our licence.

The Submitting Author accepts and understands that any supply made under these terms is made by BMJ to the Submitting Author unless you are acting as an employee on behalf of your employer or a postgraduate student of an affiliated institution which is paying any applicable article publishing charge ("APC") for Open Access articles. Where the Submitting Author wishes to make the Work available on an Open Access basis (and intends to pay the relevant APC), the terms of reuse of such Open Access shall be governed by a Creative Commons licence – details of these licences and which Creative Commons licence will apply to this Work are set out in our licence referred to above.

Other than as permitted in any relevant BMJ Author's Self Archiving Policies, I confirm this Work has not been accepted for publication elsewhere, is not being considered for publication elsewhere and does not duplicate material already published. I confirm all authors consent to publication of this Work and authorise the granting of this licence.

For peer review only

A randomised controlled trial of tailored support to increase physical activity and reduce smoking in smokers not immediately ready to quit: Protocol for the Trial of physical Activity assisted Reduction of Smoking (TARS) study.

Taylor, A.H.[1], Thompson, T. P.[1], Ussher, M.[2,3], Aveyard, P.[4], Murray, R. L.[5], Harris, T.[2], Creanor, S.[1,6], Green, C.[6], Streeter, A. [1], Chynoweth, J.[1], Ingram, W. [1], Greaves, C. J.[7], Hancocks, H. [1] (deceased), Snowsill, T.[6] , Callaghan, L.[1], Price, L. [8], Horrell, J.[1], King, J.[1], Gude, A.[1], George, M.[2], Wahlich, C. [2], Hamilton, L.[4], Cheema, K.[5], Campbell, S.[1], Preece, D.[9]

[1] Faculty of Health, University of Plymouth, Plymouth Science Park, Plymouth, PL6 8BX, UK
adrian.taylor@plymouth.ac.uk
tom.thompson@plymouth.ac.uk
siobhan.creanor@plymouth.ac.uk
adam.streeter@plymouth.ac.uk
jade.chynoweth@plymouth.ac.uk
wendy.ingram@plymouth.ac.uk
lynne.callaghan@plymouth.ac.uk
jane.horrell@plymouth.ac.uk
jennie.king@plymouth.ac.uk
alex.gude@plymouth.ac.uk
sarah.campbell@plymouth.ac.uk

[2] Population Health Research Institute, St. George's University of London, London, SW17 0RE, UK
mussher@sgul.ac.uk
tharris@sgul.ac.uk
mageorge@sgul.ac.uk
cwahlich@sgul.ac.uk

[3] Institute for Social Marketing and Health, University of Stirling, Stirling, FK9 4LA, UK

[4] Nuffield Department of Primary Care Health Sciences, University of Oxford, Oxford, OX2 6GG, UK
paul.aveyard@phc.ox.ac.uk
Louisa.Hamilton@warwick.ac.uk

[5] School of Medicine, University of Nottingham, Nottingham, NG7 2UH, UK
Rachael.Murray@nottingham.ac.uk
kelisha.cheema@hotmail.com

[6] University of Exeter Medical School, University of Exeter, Exeter, EX1 2LU, UK
C.Green@exeter.ac.uk
T.M.Snowsill@exeter.ac.uk

[7] School of Sport, Exercise and Rehabilitation Sciences, University of Birmingham, Birmingham, B15 2TT UK
C.J.Greaves@bham.ac.uk

[8] Sport & Health Sciences, University of Exeter, Exeter, EX1 2LU, UK
L.R.S.Price@exeter.ac.uk

[9] Plymouth City Council, Public Dispensary, Catherine Street, Plymouth, PL1 2AA
dan.preece@plymouth.gov.uk

Corresponding Author:
Professor Adrian Taylor, Faculty of Health, University of Plymouth, Plymouth Science Park, Plymouth, Devon, PL6 8BX.
Email: adrian.taylor@plymouth.ac.uk Tel: +44 7952400835

For peer review only - http://bmjopen.bmj.com/site/about/guidelines.xhtml

ABSTRACT

Introduction: Smoking reduction can lead to increased success in quitting. This study aims to determine if a client-focused motivational support package for smoking reduction (and quitting) and increasing (or otherwise using) physical activity (PA) can help smokers who do not wish to quit immediately to reduce the amount they smoke, and ultimately quit. This paper reports the study design and methods.

Methods & analysis: A pragmatic, multi-centred, parallel, two group, randomised controlled superiority clinical trial, with embedded process evaluation and economics evaluation. Participants who wished to reduce smoking with no immediate plans to quit were randomised 1:1 to receive either (i) tailored individual health trainer face-to-face and/or telephone support to reduce smoking and increase PA as an aid to smoking reduction (intervention) or (ii) brief written/electronic advice to reduce or quit smoking (control). Participants in both arms of the trial were also signposted to usual local support for smoking reduction and quitting. The primary outcome measure is 6 month carbon monoxide confirmed floating prolonged abstinence following participant self-reported quitting on a mailed questionnaire at 3 and 9 month post-baseline. Participants confirmed as abstinent at 9 months will be followed up at 15 months.

Ethics and dissemination: Approved by SW Bristol NHS Research Committee (17/SW/0223). Dissemination will include publication of findings for the stated outcomes, parallel process evaluation and economic evaluation in peer reviewed journals. Results will be disseminated to trial participants and health care providers.

Registration: ISRCTN47776579

For peer review only

Strengths and limitations of this study

- This is the first study to determine whether offering support to increase physical activity alongside smoking reduction is effective and cost-effective in increasing smoking abstinence among smokers not immediately ready to quit but who wish to reduce. The study involves over 900 participants recruited across four sites.
- The study's primary outcome is biochemically verified 6-month prolonged floating abstinence between 3 and 9 months post-baseline, with a secondary endpoint to consider differences at 15 months.
- The intervention involved considerable public involvement in both the pilot and current trial and was person-centred, theory based, manualised and delivered by eight health trainers who were trained to deliver behavioural support, and subsequently remotely supervised by phone or Skype.
- A mixed methods process evaluation will explore the fidelity of intervention design, delivery, receipt and enactment and explore if and how key components in our logic model operated.
- As an effectiveness trial, the intervention does not involve a supervised exercise programme and some participants entering the study may have been more interested in smoking reduction rather than increasing physical activity. This may limit the study's contribution to the exercise and smoking cessation evidence.

For peer review only

For peer review only - http://bmjopen.bmj.com/site/about/guidelines.xhtml

INTRODUCTION

Smoking remains the main cause of preventable morbidity and premature death in high-income nations. [1] The annual cost of smoking in England is estimated to be £11 billion to society, of which £2.5 billion is to the National Health Service (NHS) in England. [2] Tobacco control policies and individually targeted interventions have helped to reduce the UK population smoking prevalence rate to 14.7%, [3] but prevalence varies considerably by socio-economic and mental health status, contributing to growing health inequalities.

The UK's National Institute for Health and Care Excellence (NICE) Public Health No. 10 guidelines [4] for smoking cessation focus on identifying a quit date and abrupt cessation, with a recognition of the importance of motivational support to supplement pharmacological support. For those not intending to quit immediately, smoking reduction may lead to more quit attempts and subsequent successful abstinence, though there are limitations in the evidence [5-8] with a wide range of approaches to reduction (e.g. pharmacological, behavioural support, and self-initiated approaches). Motivational support appears to have the potential to support reduction in smoking, and the greater the reduction the greater the likelihood of successful quitting. [9]

The English Smoking Toolkit Study (2011 to 2014) suggests there is interest in smoking reduction, rather than immediately quitting, among 50% of smokers, and approximately 30% of UK smokers report using e-cigarettes to do so. [10] Other evidence based approaches to self-regulate smoking are needed as there remains uncertainty over the safety of e-cigarettes. [11] There is considerable interest in encouraging smokers to acutely and chronically use physical activity to manage smoking [12 13] though a recent systematic review revealed only 1 of 24 randomised controlled trials provided evidence that an exercise programme can aid abstinence for at least 6 months [14] among those attempting to quit. That said, most studies were of low quality and focused on proof of concept, rather than being pragmatic and offering an acceptable and feasible intervention for smokers more generally.

A unique randomised pilot study provided promising support for short-term effects of a behavioural support intervention offered by a health trainer for increasing physical activity and smoking reduction on cigarettes smoked and abstinence. [15 16] Previous studies [17] have reported that a self-determination based intervention to support autonomy and perceived competence for smokers can facilitate long-term smoking abstinence, and this approach was embedded in the pilot intervention. Intervention participants had an average 4.2 sessions by phone or face to face with the health trainer, with a range of 0-8 sessions. Compared with the control group, they were significantly more likely to achieve at least a 50% reduction in number of cigarettes smoked (39% vs 20%), to attempt to quit (22% vs 6%), and be abstinent up to 8 weeks after quit day (14% vs 4%), and at 16 weeks (10% vs 4%). A higher proportion of the intervention group also reported using physical activity for controlling smoking: 55% vs 22% and 37% vs 16%, at 8 and 16 weeks, respectively. [15] The intervention costs were approximately £192 per participant and exploratory cost-effectiveness modelling indicates that the intervention may be cost-effective. [15]

Physical activity is likely to influence smoking behaviour through both implicit and explicit processes. [12] A smoker could focus on becoming physically active which in turn has emotional benefits which may implicitly reduce cognitive and emotional triggers for smoking. Exercise may also be explicitly used to acutely manage cigarette cravings and withdrawal symptoms, [18 19] and chronically manage weight gain associated with smoking reduction or help in a shift towards a healthier identity.

In prospective population surveys and trials, weight gain and fear of weight gain is associated with reluctance to quit smoking and remain abstinent, especially among women and initially heavier

For peer review only - http://bmjopen.bmj.com/site/about/guidelines.xhtml

smokers, [20-22] with a meta-analysis study reporting an average of 4.67kg (95% confidence interval: 3.96 to 5.38) gained after 12 months of abstinence. [23] There is evidence that physical activity is effective for preventing long-term weight gain after smoking cessation, [24] not only by increased energy expenditure and metabolic rate, but also through self-regulation of energy intake, particularly emotional snacking. [25] Finally, as a result of increasing physical activity, a smoker may begin to establish a different identity (e.g. investing in personal fitness and generally becoming a "healthy person"), which in turn may trigger a desire to reduce harm from smoking through reduction and ultimately quitting. [15]

Following a successful pilot study [15] there is a need to establish the effectiveness and cost-effectiveness of behavioural support for increasing physical activity and reducing smoking on longer term abstinence among smokers not immediately ready to quit.

AIMS & OBJECTIVES

The overarching aim of the trial is to determine if an individually-tailored behavioural intervention for smokers wishing to reduce but not immediately quit provides an effective and cost-effective approach to supporting increases in physical activity and smoking reduction, resulting in increased rates of quit attempts and subsequent 6-month prolonged smoking abstinence.

The specific aims of the trial are to determine whether the intervention, compared with support as usual (SAU), will:

- Increase the proportion of participants who achieve 6 month prolonged biochemically-verified floating abstinence at 9 months post-baseline.
- Increase the proportion of participants who self-report a reduction in number of cigarettes smoked (between baseline and both 3 months and 9 months) of at least 50%.
- Increase the proportion of participants who achieve biochemically-verified prolonged abstinence at 15 months post-baseline (i.e., 12 months post-intervention).
- Increase self-reported physical activity at 3 and 9 months post-baseline, and accelerometer assessed physical activity at 3 months post-baseline.
- Improve quality of life, self-reported weight and cigarette cravings at 3 and 9 months post-baseline.

Further aims are:

- To conduct a health economic evaluation to estimate the costs of delivering the intervention and differences in health and social care costs between intervention and SAU at 9 months post-baseline. This will also estimate the cost-effectiveness of the intervention compared with SAU at (i) 9 months and (ii) over a longer term / lifetime horizon.
- To conduct an embedded mixed methods process evaluation to explore the mechanisms of action of the intervention and acceptability of study processes.

The current paper outlines the protocol for the TARS trial.

METHODS & ANALYSIS

For peer review only - http://bmjopen.bmj.com/site/about/guidelines.xhtml

This protocol is reported in accordance with the SPIRIT guidance [26] for protocols of clinical trials and the TIDieR guidelines [27] for intervention description.

Study design & setting

The TARS trial is a pragmatic, multi-centred, parallel, two group, randomised controlled superiority clinical trial to compare (i) tailored support to reduce smoking and increase physical activity (PA) as an aid to smoking reduction (intervention) with (ii) brief advice to reduce or quit smoking (control). The study includes a mixed methods embedded process evaluation and health economics evaluation. Recruitment to the trial was over 16 months (January 2018 – May 2019) in the geographical areas in and around four cities in the UK: Plymouth, Nottingham, London, and Oxford.

Study population

Potential participants were approached from primary and secondary care establishments and also from the community. Participants were adult smokers (≥ 18 years) who smoked ≥ 10 cigarettes per day (for at least 1 year), wished to reduce smoking but not quit immediately. We accepted smokers who were also using other nicotine containing or other cigarette management products. Smokers were ineligible if they were unable to engage in at least 15 minutes of moderate intensity PA, had any illness or injury that might be exacerbated by exercise, were unable to engage in the study and/or the intervention due to language or for other reasons (e.g. provide an unacceptable level of risk to the health trainer or researcher). All ineligible smokers were referred for smoking cessation advice in line with local usual practice.

Study procedures

Participant identification, approach and consent: A broad range of participant identification and approach methods were employed in an attempt to reach many different demographic groups and make the study as inclusive as possible. The most common approach method was through a search of a primary care practice patient database, with invitations sent to patients who were listed as smokers offering them the chance to get in touch with the trial team. In the early phase of recruitment, a study within a trial (SWAT) was conducted at GP practices at one research site, to compare the efficiency and cost-effectiveness of three different invitation methods (full information pack, single page invite, text message). [28] Other approaches were via secondary health care and stop smoking services (for those who had failed to quit), and by social media, pharmacies, dental practices, community organisations and local businesses using posters and leaflets.

As shown in Figure 1 (participant flow chart), smokers expressing interest in the trial were screened for eligibility by researchers at each site. If suitable and still interested in joining the study, participants provided evidence of consent either at a face-to-face meeting or via telephone.

Baseline assessment: Participants then completed a baseline questionnaire with a member of the local study team either face-to-face, by mail, or over the telephone. The schedule of measures and data collection at baseline and follow-up are shown in Table 1.

For peer review only - http://bmjopen.bmj.com/site/about/guidelines.xhtml

Table 1. Schedule of baseline and follow-up measures

	Screening & Baseline	Month 3	Month 9	Month 15 #
Demographics (e.g., age, gender, education attained, employment status)	X			
Self-reported cigarettes per day (or equivalent)	X	X	X	X
Reduction of ≥ 50 % in number of cigarettes smoked since baseline *		X	X	
Biochemically confirmed abstinence (self-reported quitters only)		X	X	X
Self-reported floating prolonged abstinence (since quitting smoking, with quit date, if relevant) over at least 6 months *			X	X
Accelerometer assessed minutes of moderate & vigorous physical activity in a sub-sample		X		
Self-reported 7-day recall of physical activity	X	X	X	
Heaviness of Smoking Index	X			
Use of smoking management products	X	X	X	X
Urge & strength of urge to smoke	X	X		
Engagement with the health trainer intervention (8 weeks, plus optional 6 weeks additional support if a quit attempt is made)		X		
Health & social care utilisation	X	X	X	
Health related quality of life (EQ-5D-5L & SF12)	X	X	X	
Self-reported weight & height (to calculate BMI)	X	X	X	
Self-reported process measures:<br>Importance and confidence in smoking reduction and cessation<br>Importance and confidence in being physically active<br>Action planning to change smoking<br>Action planning to change physical activity<br>Self-monitoring of smoking<br>Self-monitoring of physical activity<br>Availability of support to reduce smoking<br>Availability of support to increase physical activity<br>Use of physical activity for smoking regulation	X	X		
Serious adverse events (self-reported)		X	X	
Qualitative process evaluation (in parallel throughout) (sample)	X	X	X	X

Only participants with biochemically verified abstinence at 9 months are followed-up at 15 months post-baseline

* Derived measure

For peer review only

Randomisation: Participants were individually randomised to either the intervention or control group (1:1 ratio) following completion of baseline assessments, to ensure concealment was preserved. Randomisation was achieved by means of a 24-hour web-based system created by the UKCRC-registered Peninsula Clinical Trials Unit (CTU) in conjunction with a statistician independent from the trial team, and used random permuted blocks, with stratification for recruitment site and a dichotomised low/high score derived from the heaviness of smoking index (HSI).[29] The HSI score is calculated from summing responses to two questions:

i. when the first cigarette is smoked after waking, scored as >60 minutes (score 0); 31-60 minutes (1); 6-30 minutes (2); and within 5 minutes (3)
ii. how many cigarettes are smoked in a typical day, scored as ≤10 cigarettes (0); 11-20 cigarettes (1); 21-30 cigarettes (2); > 30 cigarettes (3)

Total HSI scores of 0-4 were categorised as low, and 5-6 were considered high for the purposes of stratification.

Following randomisation, all participants were sent a letter from the co-ordinating CTU confirming which trial arm they had been assigned to, and a guidance sheet on usual support locally for smoking reduction and cessation. The participant's GP was also sent a letter notifying them that one of their patients is participating in the study.

Blinding: It was not possible to blind participants to their allocated group. Every effort was made to ensure that the trial team remained blind to the allocation of each participant when collecting follow-up data (including researchers collecting carbon monoxide (CO) measurements), but this was not always possible. Health trainers delivering the intervention were obviously aware of the participant's allocation to trial arm, but were discouraged from communicating with site researchers about this. Questionnaire booklets and accelerometers were mailed out from and returned to the CTU without knowledge of trial arm allocation.

Follow-up: At 3 and 9 months post-baseline all participants are posted a questionnaire booklet and a freepost envelope to return the completed booklet to the CTU. A £20 shopping voucher is mailed to participants upon CTU receipt of the completed booklets at both 3 and 9 months. To increase response rates, motivational postcards are mailed to participants before the follow-up questionnaires are sent out. Up to two reminder letters are issued (and a further three telephone calls as required) to remind participants to return the questionnaire booklets, and the option of the participant telephoning a member of the research team to aid completion of the booklets is offered. Participants are given the option to just complete the key questions about smoking behaviour if the questionnaire booklet is not returned to the CTU within 2 weeks and to submit these responses by e-mail, phone or text if preferred to maximise follow-up data on key outcomes. If participants do not complete the key questions (regardless of method) within a 4 week window they are categorised as not completing follow-up at that time point.

All participants who report having made a quit attempt in the questionnaire booklet and not smoked since that date at 3 and 9 months are contacted for biochemical verification of abstinence.

At 3 months post-baseline, approximately 20% of participants were sent wrist-worn waterproof accelerometer (GeneActive Original accelerometer®, Activinsights, Kimbolton, UK, http://www.geneactiv.org/) with instructions to wear constantly for one whole week (day and night), and a freepost return envelope to be sent to the CTU. To maximise data completeness, participants scheduled to receive an accelerometer were sent a standardised letter from the CTU, two weeks before

For peer review only - http://bmjopen.bmj.com/site/about/guidelines.xhtml

receiving the accelerometer, advising them that they would shortly be receiving the device, and asking them to inform the CTU if they were unable to wear it. A letter was sent to participants who did not object to wearing the device, 3 days into the 10 day recording period prompting participants to start wearing the device if they had not already done so. Up to two reminder letters and a follow-up phone call were made to participants if they did not return the accelerometer.

Trial allocation groups

Intervention: The TARS intervention was based on the intervention developed for the EARS pilot trial, with further refinement based on feedback from the EARS process evaluation. [15] Throughout the development of the intervention for the EARS and TARS studies, we engaged with smokers from a wide range of socio-economic backgrounds to ensure an acceptable person-centred approach was embedded.

Throughout the pilot trial and before commencing the definitive trial, we conducted individual and focus group discussions with smoking-cessation practitioners, researchers, public health consultants, community workers (including volunteers) and people who currently or previously smoked. We reviewed literature on using exercise as an aid to quitting, and consulted with academic experts on behaviour change for physical activity, smoking reduction and smoking cessation. These activities informed the intervention principles and theoretical basis, structure and delivery.

The intervention aimed to be empowering and put the client at the centre of the decision making process. All aspects were designed to promote self-determined behaviour, focussed on elements of self-determination theory which emphasises people's sense of autonomy, competence, and relatedness. [30-32] This was in part achieved by adopting motivational interviewing principles [33] as the guiding delivery style for the practitioners which have been proposed to enhance and promote self-determined behaviour. [34]

An intervention delivery model, or "roadmap", was developed between the pilot and definitive trial (Figure 2) to aid in the conceptualising of the intervention process and support the manualisation of the intervention and training of the practitioners. This was supported by the development of a set of "core competencies" (Table 2) developed from the pilot trial which outlined key processes, components, and behaviour change techniques that the health trainers were expected to deliver and form the basis of fidelity assessment. [35] A comprehensive training manual was developed, outlining all the skills, behaviour change techniques and strategies to support behaviour change intended for use in the TARS study. This was used as the basis for a 3 day training course, which was delivered by TT, AT, CG, and LC. Health trainers then engaged with a wide range of "practice participants" to complete their training. The health trainers attended regular (bi-weekly for the first 3 months and monthly thereafter) two-hour formative feedback supervision teleconferences throughout the study period to help to embed skills and to benefit from each other's shared experiences. Individual supervision for the health trainers was available as needed, provided by the intervention lead (TT).

For peer review only - http://bmjopen.bmj.com/site/about/guidelines.xhtml

Table 2 Intervention components, aims, content and indicative change in processes

Intervention components	Aim	Content	Indicative change in processes
Active participant involvement (1)	Develop rapport, build trust, and shared respect.	Effective communication skills. Build autonomous support.	Participant feedback on health trainer-led support.
Build motivation to reduce smoking (2) and increase physical activity (3)	Identify ambivalence towards reduction & quitting. Build self-awareness & confidence to cut down and increase physical activity.	Help smoker to identify importance & challenges of reduction & cessation, and implicit & explicit roles of physical activity (motivational interviewing techniques).	Smoker has desire and confidence to cut down and perhaps quit over the early sessions, and increase physical activity. Smoker engages in more self-monitor of smoking and physical activity behaviour.
Self-monitor smoking and physical activity and set goals to reduce smoking (4) and increase physical activity (5)	Develop strategies to reduce smoking and increase physical activity.	Set SMART goals to reduce smoking and increase physical activity. Signpost to physical activity opportunities & remove barriers to do physical activity.	Goals identified and action plans developed. Smoker engages in more goal setting to reduce smoking and increase physical activity behaviour.
Review/problem solving for smoking (6) & physical activity (7)	Build confidence, perceptions of control, & self-regulation skills.	Smoker reflects on smoking reduction and physical activity, identifies barriers and possible solutions, increases and sets new targets; perhaps to quit.	Goals revised to reflect confidence to increase physical activity, reduce smoking, and possibly quit.
Integrating idea of changing smoking and physical activity (8)	To help smoker to identify any links between smoking and physical activity	Explore with smoker how physical activity may influence smoking (and vice versa) (person centred exchange of information (Ask-Tell-Discuss)).	Smoker increases use of physical activity as an aid to smoking reduction.
Reinforce health identity shift (9)	To help identify shift from smoker to healthier identity.	Smoker reflects on label as heavy – moderate – light or non-smoker status, and more active person.	Decrease in importance of smoking and increase in importance of doing physical activity identified.
Manage social influences on smoking (10) and physical activity (11)	To involve others in process of reducing smoking and increasing physical activity. Manage negative or undermining social influences.	Smoker identifies key others who can support reduced smoking (or cessation) and increasing physical activity, and engages with them in preferred ways. Uses negotiation and discussion to manage negative social influences.	Support from others identified as important and used for smoking reduction or cessation, and increasing physical activity.

For peer review only - http://bmjopen.bmj.com/site/about/guidelines.xhtml

Participants allocated to the intervention arm were offered individually tailored behavioural support from a health trainer. The health trainer delivered the processes outlined in Figure 2 and Table 2, with the option of up to 8 weekly sessions, and a further 6 sessions if the participant wanted support after quitting, and aimed to empower participants to decide what support was offered, and where, when and for how long. Signposting to local smoking cessation support services was also offered to those wishing to quit. If a smoker wished to reduce smoking using e-cigarettes or licenced nicotine containing products (LNCP) they were also offered any local available support for this.

Health trainers were appointed on the basis of having good communication skills, including empathy, and at least some training and experience in supporting health behaviour change. All health trainers had at least a first degree in a related field although this was not a pre-requisite for the role. The health trainer was trained to support change in both smoking and physical activity and help individuals to make the connections between the two. As described in Table 2, the core intervention processes that the health trainer was trained to deliver were: (1) building rapport and supporting autonomous behaviour change; (2) building motivation; (3) supporting self-monitoring and goal setting; (4) problem solving; (5) integrating smoking and PA behaviour; (6) supporting a health identity shift; (7) supporting the management of social influence on behaviours.

The support offered was broadly structured (based on delivering the processes outlined in Table 2 and Figure 2) but health trainers were trained to be flexible in their approach, to tailor it for individual needs and preferences. They were trained to do this using a person-centred approach and principles from motivational interviewing such as showing empathy and reflective listening. [36] They were also trained to assess (and be aware of) participants' needs in relation to the core psychological needs posited by Self-Determination Theory, [37] which we referred to in the training as "the three Cs": Control (having choice /autonomy in decision-making around behaviour change); Competence (developing self-efficacy /building confidence in the ability to change) and Connectedness (social acceptance of the new behaviour /support from important others for making changes).

A fuller intervention description, including the trainer manual and intervention materials will be published as supplementary materials linked to the final NIHR project report.

Support as usual (SAU): Participants allocated to both arms of the trial received written guidance for smoking reduction and cessation, including web links to what is offered at local level, or paper versions of this information. Typically, there are no formal programmes for use of medication to support reduction (rather than abrupt stopping) and people usually buy their own replacement therapy or e-cigarette product.

Determination of sample size

Since the planned primary analysis is a comparison of the proportions of the binary primary outcome, the sample size calculation was based on a two-sided Fisher exact test. An abstinence rate of 5% for the control group and detectable effect of 6% (i.e. an increase from 5% to 11% due to the intervention) are conservative estimates consistent with those from the EARS pilot study [15] and those reported from a systematic review of pharmacological interventions. [38] The corresponding odds ratio for this effect size is 2.35. Participants with missing outcome data will be assumed to be still smoking following the Russell Standard, [39] and the numbers of participants in each allocated group are assumed to be in the ratio of 1:1. Under these conditions, according to Stata v14.2, the minimum number of participants required to detect an abstinence rate of 11% compared with that of 5% in the control group, with a significance level of no more than 5% and power of at least 90%, is 900, above which a power in excess of 90% is maintained.

For peer review only - http://bmjopen.bmj.com/site/about/guidelines.xhtml

Outcome measures

Table 1 lists the outcomes measures and when they are being assessed.

Primary outcome measure: Biochemically verified 6 month floating prolonged abstinence between 3 and 9 months. [40] Abstinence will be confirmed by expired CO <10ppm measured with a CareFusion MicroCO meter (Williams Medical Supplies, Rhymney, UK, www.carefusion.co.uk) at a face-to-face visit.

Participants who self-reported abstinence at 3 months and who were confirmed as abstinent through biochemical verification via expired CO at a face-to-face assessment and then self-report abstinence (and not having smoked even a puff since the 3 month assessment) at 9 months, again confirmed by expired CO, will be defined as having prolonged abstinence over at least 6 months.

Other smoking-related measures: Only participants who have biochemically verified abstinence at 9 months are being contacted at 15 months post-baseline to assess floating prolonged abstinence over a period of 12 months (3 to 15 months). Participants who were not abstinent at 3 months but have biochemically confirmed abstinence at 9 months will be contacted at 15 months to confirm floating prolonged abstinence of 6 months between 9 and 15 months. As a contingency measure for verification of abstinence during the coronavirus (COVID-19) outbreak, abstinence will be confirmed by saliva cotinine level <12ng/mL[41] using a mailed self-collection kit and assay provided by ABS Laboratories (York, UK, www.acmgloballab.com). This contingency measure will apply to follow-up for secondary outcomes for a minority of participants. While the intervention is expected to primarily influence quitting in the first 3 months of the study, it is possible that a sustained quit attempt occurs after the 3 month assessment as a result of the health trainer building behaviour change skills which are used subsequently to reduce and then quit smoking.

Participants are asked to self-report the number of cigarettes smoked and type of nicotine product (i.e. pipes, cigars and roll your own). We estimated 0.45 grams of tobacco was the equivalent of one cigarette based on a previous rigorous study [42] and reported in the EARS pilot study. [15] From this we will estimate the number of cigarettes smoked at follow up, and also calculate if participants reduce smoking by at least 50% between (i) baseline and 3 months and (ii) baseline and 9 months.

PA measures: The 7-day recall measure of PA [43] is used to assess self-reported weekly minutes of moderate and vigorous PA. An objective measure of total weekly minutes of moderate and vigorous physical activity (MVPA) was collected with a wrist-worn waterproof GENEActiv accelerometer [44] around the 3 month follow-up time-point. Participants were asked to wear the accelerometer on the wrist of the non-dominant hand constantly for one week and then return to the CTU. This accelerometer shows the wearer no information about their PA levels and does not have obvious motivational value.

Physical measures: Participants are asked to self-report their height and weight from which body mass index will be calculated.

Health related quality of life measures: The EQ-5D-5L (EuroQol Group, 1990) comprises the following five dimensions: mobility, self-care, usual activities, pain/discomfort and anxiety/depression. Each dimension has five levels: no problems, slight problems, moderate problems, severe problems, and extreme problems. [45] The SF-12 is a 12-item, patient-reported survey of patient health, consisting of twelve questions. [46]

Resource use / health care service use: Use of primary and community-based health and social services, and hospital-based in-patient and out-patient services are captured using a resource use questionnaire developed in two pilot trials involving health trainer led interventions. [16 47] It sought to capture the number of contacts that occurred (if any) with a range of health and social care professionals and where

For peer review only - http://bmjopen.bmj.com/site/about/guidelines.xhtml

those contacts took place since completing the previous survey, using both fixed and open format responses. Reasons for hospital admissions were also requested.

Self-reported process measures: Single survey items, using a 5-point Likert scale (Strongly disagree to Strongly agree) assessed the psychological and behavioural processes that the intervention was designed to influence as shown in Table 2. These were used in other trials. [15 48 49] A single survey item, using a 6-point scale (Not at all to All the time), was used to assess frequency of urge to smoke in the past week. [50] A single survey item (6-point Likert scale, no urges to extremely strong) assessed strength of urges to smoking in the past week [50] as used previously. [15]

Economic evaluation

The economic evaluation will (i) estimate the long-term cost-effectiveness of the TARS intervention plus SAU versus SAU alone, over a life time horizon using a model-based economic evaluation, and (ii) estimate the cost-effectiveness of the TARS intervention plus SAU versus SAU alone over the primary 9-month trial follow up, in an economic evaluation conducted alongside the trial. The longer-term model-based economic analysis is considered the primary economic analysis, consistent with the approach commonly applied in the context of cost-effectiveness analysis in smoking cessation settings.

The primary perspective of the economic analyses will be that of the NHS and Personal Social Services (i.e. Third Party Payer), with a broader perspective explored in sensitivity analyses. The primary economic endpoint will be the quality-adjusted life-year (QALY, using EQ-5D data), with results presented as incremental cost effectiveness ratios (ICERs) representing estimated costs per QALY gained.

The economic analysis will be undertaken against a pre-defined health economics analysis plan (HEAP), which is available on request. In summary, the trial-based cost-effectiveness analysis will use participant level data collected within-trial to estimate (i) the resource use and costs associated with the delivery of the TARS intervention, (ii) broader resource use and costs associated with health and care service use by group, (iii) QALYs by group, and (iv) the incremental cost per unit of outcome (e.g. cost per incremental QALY, cost per quitter) over the 9-month follow-up. In this analysis, EQ-5D-5L data will be used to estimate QALYS, deriving health state values at each time point, using the published tariff values for England (presently recommendations are for values to be derived using methods reported by Van Hout et al [51]), and using the area under the curve approach.[52] Analyses will be based on an ITT principle, using a complete case analysis, and will assess uncertainty, and include detailed sensitivity analyses.

The model-based economic evaluation will adopt a longer term perspective (lifetime) beyond the trial follow-up, to present a policy relevant cost effectiveness analyses, that predicts future costs and QALYs after the trial endpoint based on the reported effectiveness of the TARS intervention. A decision analytic model will be used, with the model based on the model developed and described in the prior pilot study (EARS), [15] which we will update and adapt using a review of the recent literature on modelling in this area and based on input from a Stakeholder Group. The model-based evaluation will be based on good practice guidelines for decision analytic modelling in the HTA context. [53-55]

Embedded mixed methods process evaluation

A mixed methods process evaluation will focus on trial processes and methods and will attempt to understand the effective components and processes of the intervention. During the internal pilot phase, the evaluation will focus on barriers and facilitators for recruitment methods, initial intervention engagement, and early intervention implementation. For the subsequent main trial, the evaluation will

For peer review only - http://bmjopen.bmj.com/site/about/guidelines.xhtml

focus on acceptability of study processes (via a qualitative sub study), intervention engagement levels, predictors of intervention engagement, intervention delivery fidelity, and evaluating the implementation of the intervention process model (participant understanding of the intervention model (receipt fidelity), mediating effects of process measures on PA and smoking outcomes (enactment fidelity), mediating effects of PA on smoking outcomes, approaches and acceptability of smoking reduction, multiple behaviour change, progression to cessation, and other perceived effectual intervention components).

Data for the process evaluation will be collected via: the trial database, audio recordings of intervention sessions, and audio recorded and transcribed interviews with trial participants, research assistants, health trainers, and GPs/practice managers.

Trial data handling

Data are collected and maintained in accordance with the current legal and regulatory requirements. A data management protocol has been produced by the CTU to ensure secure data collection and storage in accordance with the Data Protection Act 1998, and later conforming to the General Data Protection Regulation 2016 and Data Protection Act 2018.

Electronic study records will be held over the lifetime of the study in secure storage solutions aligned with the host institution's information security classification policy. At the time of writing, electronic study data are stored in a SQL server database on a restricted access, secure server maintained by the University of Plymouth. Data are entered into the database via a bespoke web-based data entry system encrypted using SSL version 3 (QuoVadis Global, http://www.quovadisglobal.com). Access to identifiable information is restricted and permission-based.

A parallel, linked, bespoke data system has been used to manage intervention engagement post-randomisation. The system captures all health trainer attempted and actual contact with intervention participants in real time to produce summary data (e.g., number of sessions, duration and mode of sessions, notes on session content) and aid supervision sessions, and intervention management (e.g., should a health trainer be unavailable).

Identifiable information will be omitted from the transcriptions of the process evaluation interviews.

Statistical analysis plan

A detailed statistical analysis plan was drafted during the trial delivery phase and will be approved by an independent statistician and wider Trial Steering Committee, prior to database lock. The analyses will be reported in full and in accordance with the Consolidated Standards of Reporting Trials (CONSORT) guidelines. [56] The main planned analyses are summarised below.

Baseline characteristics and summary statistics

Descriptive statistics by allocated group will be presented for the baseline, and primary and secondary outcomes, which includes the smoking outcomes and questionnaire data as well as the smoking and

For peer review only - http://bmjopen.bmj.com/site/about/guidelines.xhtml

physical mediators, with the exception of the primary outcome and secondary abstinence measures (assessed at 3, 9 and 15 months only) and accelerometer outcomes (assessed only at 3 months).

For continuous outcomes, summary information will be presented in the form of means alongside standard deviations (SD). Count and skewed continuous data will be presented in terms of median, and inter-quartile range. For categorical outcomes, summary information will be presented in the form of frequencies and percentages.

Inferential statistical comparison at baseline of randomised groups is not good practice [57] and it is expected that participants in both groups will, on average, be similar. Following initial primary analysis, if substantial imbalance at baseline is identified in any key variables, such as gender and age, the importance of any imbalance will be noted and additional adjusted analyses may be performed.

Primary analysis

The null hypothesis is that there is no difference in CO verified 6-month prolonged floating-abstinence rates between the intervention and control groups at 9 months post-baseline. In line with the Russell Standard schedule, [39] the primary comparative analysis will be conducted on an intention-to-treat basis, in which participants with missing responses will be considered to still be smokers. Interpretation of the primary effectiveness analysis will be based on the odds ratio from the logistic regression model adjusted for (fixed effect) stratification variables: site as a factor and HSI as an ordinal covariate. Both the adjusted (primary analysis) and unadjusted odds ratios and corresponding 95% confidence intervals will be presented. Primary effectiveness shall also be presented as a relative risk along with the absolute between-group differences in abstinence rates, as recommended in the CONSORT guidelines for parallel group randomised trials. [58]

Planned sensitivity analysis of the primary outcome:

(a) Rather than assuming participants with missing responses at 3 or 9 months were still smoking, the primary outcome will be imputed under a number of varying assumptions and the primary analysis re-run for each of the scenarios;
(b) A complier average causal effect (CACE) analysis will be undertaken, if greater than 20% of participants allocated to the intervention group are categorised as not having completed at least two interventions sessions with a health trainer, with individual participants in the intervention group categorised as compliers if they completed at least two intervention sessions. Participants in the control group and non-compliers in the intervention group will be compared to compliers in the intervention group.

Secondary analyses

To explore whether the primary outcome was influenced by the intervention dose actually received (i.e. number of health trainer sessions attended) the primary outcome shall be modelled on the number of health trainer sessions attended in the intervention group only, adjusting for the stratification variables. Although the trial is not powered to detect the influence of moderating factors on the primary outcome, secondary analyses will be undertaken to explore whether the intervention effect is modified by key demographic and/or behavioural factors at baseline. These are pre-specified as the postcode-based index of multiple deprivation (IMD); the factor indicating smoking cessation medication or a vaping product at baseline; MVPA level, confidence to quit; and the stratification variable, HSI. The multi-variable logistic regression model outlined above will be extended to include the interaction term of

For peer review only - http://bmjopen.bmj.com/site/about/guidelines.xhtml

allocated group and each of the listed potential modifying variables. Evidence of an interaction shall be interpreted through the 95% confidence intervals of the coefficient for the interaction term.

During the development of this study, the potential health trainer effect was considered at length. Given the lack of evidence on individual health trainer effects, the study design and sample size calculations do not allow for such partial clustering within health trainers (within recruitment sites). However, an exploratory analysis of the intervention effect will be undertaken using a multi-level, mixed-modelling approach, to allow for the partially nested data: participants allocated to the intervention group will be partially clustered within the health trainer, in turn nested within sites.

Analysis of secondary outcomes**:** Between-group comparisons will be undertaken, including for the following key secondary outcomes:

- Biochemically verified point prevalence abstinence at 3, 9 and 15* months post-baseline
- Self-reported point prevalence abstinence at 3, 9 and 15* months post-baseline
- Prolonged biochemically verified abstinence over 6 months between 9 and 15 months post-baseline
- Prolonged biochemically verified abstinence for at least 12 months between 3 and 15 months post-baseline (derived from biochemically confirmed abstinence at all three follow-up time points)
- At least a 50% reduction in reported smoking levels between i) baseline and 3 months and ii) baseline and 9 months
- Number of cigarettes used on an average day over the past week (incl. equivalent cigars, tobacco) at 3, 9 and 15* months post-baseline
- Total number of LNCP used on an average day over the past week at 3, 9 and 15* months post-baseline
- Total number of self-reported minutes engaged in MVPA over the past week at 3 and 9 months post-baseline
- BMI at 3 and 9 months post-baseline

NB: * only for those with biochemically confirmed abstinence at 9 months.

The following data is derived from accelerometers mailed to a sub-group of participants in both arms of the trial along with the 3 month questionnaire, which are returned after the accelerometers have been worn for seven days:

- The average time spent in moderate to vigorous activity over the past week
- The average daily time spent sleeping over the past week

No adjustment for multiple analyses will be made; such adjustment methods are too conservative when outcomes are positively correlated, as they would be in this trial. Analyses will use multi-variable linear regression (continuous outcomes) or logistic regression (binary outcomes) to compare each of these secondary outcomes between allocated groups, with adjustment for site as a factor, and HSI, as an ordinal variable, as well as baseline values of the outcome as appropriate. As accelerometer data were only available at 3 months, only summary statistics for weekly minutes of MVPA, shall be presented by allocated group without adjustment for baseline variables.

The between-group comparisons of continuous outcomes will be reported as mean differences together with 95% confidence intervals, unless the outcomes are substantially skewed. Both adjusted and unadjusted differences will be presented. The between-group comparisons of binary outcomes will be

For peer review only - http://bmjopen.bmj.com/site/about/guidelines.xhtml

reported as the adjusted and unadjusted odds ratios with conversion to relative risks and corresponding confidence intervals, along with the absolute between-group differences in abstinence rates.

Analyses will be undertaken to investigate whether any effect of the intervention in terms of reduction in smoking at 3 months and 9 months is modified by key sociodemographic and/or behavioural factors at baseline. These pre-specified factors at baseline are using smoking management medication or vaping, IMD, the stratification variables, MVPA level, and confidence to quit. The multi-variable models will be extended to include the interaction term of allocated group and each of the potential modifying variables.

Model checking: The logistic regression model for the primary analysis is pre-specified. However, observations identified as potential outliers through their influence on the model, may be excluded as part of a sensitivity analysis. The distributional assumptions of multi-variable linear regression models will be visually assessed through plots of residuals. If there are concerns about distributional assumptions being met, bootstrapped confidence intervals for the adjusted between-group differences will be produced.

Mediational analysis

If there is evidence that the intervention is effective, an exploratory mediational analysis will be undertaken to determine if any effect of allocated group on the primary outcome was mediated by changes in smoking and/or physical activity between baseline and 3 month follow-up, adjusted for the stratification variables (site and HSI). Further analyses will explore if changes in smoking and physical activity are mediated by changes in outcomes from the questionnaire on attitudes to smoking between baseline and 3 months (importance and confidence to reduce smoking/increase physical activity; self-monitoring and goal setting; urges to smoke).

For peer review only

For peer review only - http://bmjopen.bmj.com/site/about/guidelines.xhtml

Discussion

The manuscript describes the methods involved in the trial design and intervention delivered.

Patient & public involvement

The TARS research team has worked with people who smoke, not as research participants, individually and in groups from across all communities, to guide research questions, study design and conduct, intervention development and dissemination over the past 15 years. As an example of their input, they had varying views on the merits of vaping to reduce smoking and how various forms of PA may help. A university employee and non-employee PPI group (of people who currently or have previously smoked) regularly met to input into intervention and trial methods development. There was further PPI input into project management group meetings and trial steering committee meetings throughout the trial. The study team has also engaged with key stakeholders involved in commissioning and delivering community interventions to assess where the proposed intervention would best fit and the study team will continue to do this prior to and during intervention development.

Trial monitoring & oversight

The trial Project Management Group includes a multidisciplinary team of clinicians and researchers with expertise in all aspects of trial design, intervention development and delivery, conduct, analysis and quality assurance. The Trial Steering Committee (TSC) involves independent expertise to help guide the trial on behalf of the funders and provide oversee trial progress. The TSC will also sign-off the Statistical and Health Economic Analyses Plans. The Data Monitoring Committee (DMC) provides independent expertise and support particularly regarding evidence or reason why the trial should be amended or terminated based on recruitment rates, compliance, safety or efficacy.

Ethics & dissemination

The study has been approved by the South West – Central Bristol Research Ethics Committee (REC reference: 17/SW/0223) and the Health Research Authority. A number of approvals have been granted for minor and substantial amendments; the amendment history and full details of the amendments are available upon request.

The research team will work with stakeholders and PPI representatives at each site, and nationally, to help to interpret the results and the implications for policy and practice. The PMG will establish a publication plan and authorship rules. Reporting will follow current CONSORT guidelines for randomised trials. The study results will be submitted for publication in relevant international, high impact, peer reviewed journals. Names of key collaborators and groups who have contributed to the trial will be clearly stated in all publications. The study findings will be presented at regional, national and international meetings as appropriate.

Safety considerations

The recording and reporting of non-serious adverse events (AEs) in this low-risk study is not required. Serious Adverse Events (SAEs) will be documented from the time of participant consent until a maximum of 8 weeks after the follow-up assessment at 9 months. A protocol for identifying, reporting and managing SAEs has been established by the CTU, in conjunction with the Project Management Group, DMC and TSC, and conforms to the requirements of the trial Sponsor and NHS REC.

Access to data

For peer review only - http://bmjopen.bmj.com/site/about/guidelines.xhtml

The CTU Data Manager is the custodian of the final trial data set, with the exception of the audio files and transcripts arising from qualitative interviews with participants which are held by the process evaluation team.

Access will be granted to the Sponsor and host institution for the purposes of study-related monitoring, audits and inspections.

Members of the study team will have restricted access to the anonymised dataset for the purposes of conducting the trial, and to undertake the statistical and economic data analysis and the process evaluation.

Data requests should be submitted to the corresponding author for consideration. Following publication of the primary results of the trial, access to available anonymised data may be granted depending on review of the data request and appropriate agreements being in place.

Current study status

The TARS study completed participant recruitment in May 2019. Data collection for the 9 and 15 month follow-up assessments are expected to be completed in April and October 2020, respectively, and results are expected to be published in mid 2021.

For peer review only

List of abbreviations

BMI	body mass index
CACE	complier average causal effect
CO	carbon monoxide
CTU	Peninsula Clinical Trials Unit
DMC	Data Monitoring Committee
EARS	A pilot randomised trial to assess the methods and procedures for evaluating the clinical effectiveness and cost-effectiveness of Exercise Assisted Reduction then Stop (EARS) among disadvantaged smokers
EQ-5D-5L	EuroQo -5 dimension – 5 level
HSI	Heaviness of Smoking Index
ICER	incremental cost effectiveness ratio
IMD	Index of Multiple Deprivation
LNCP	licenced nicotine containing products
MVPA	moderate and vigorous physical activity
NICE	National Institute for Health and Care Excellence
PPI	Patient and Public Involvement
QALY	Quality Adjusted Life Years
SAE	Serious Adverse Event
SAU	support as usual
SF12	12-Item Short Form Health Survey version 2
SQL	Structured Query Language
SSL	Secure Sockets Layer
TARS	Trial of physical Activity assisted Reduction of Smoking
TSC	Trial Steering Committee
UKCRC	UK Clinical Research Collaboration

For peer review only - http://bmjopen.bmj.com/site/about/guidelines.xhtml

Contributors

AHT conceived the idea for the study with TT, MU, PA, CG, S.Cr and CJG.
AHT, TT, MU, PA, CG, SCr, CJG and HH contributed to the final study design and development of the protocol.
AHT, TT and CJG developed the intervention, with LC who supported PPI input.
DP provided a public health perspective on usual community Stop Smoking Service support.
AHT, PA, MU, TH and RLM were the Principal Investigators at participating sites.
JK, AG, MG, CW, LH and KC (Research Assistants) led recruitment, baseline data collection, and face to face carbon monoxide verified abstinence assessments at participating sites.
LP was consulted on accelerometer data capture and processed the accelerometer data.
SCr, AS and JC provided the statistical analysis plan.
CG developed the health economics analysis plan with TS.
TT developed the process evaluation plan with CJG, LC and JH.
HH, WI and SCa were the PenCTU trial managers.

Disclosure of potential competing interests

AG, AS, AHT, CJG, CG, CW, DP, HH, JC, JK, LC, LH, MG, MU, PA, RLM, SCr, SCa, TH, TS, TT and WI report a grant from NIHR (NIHR HTA award ref 15/111/01) during the conduct of the study.

PA is an NIHR Senior Investigator and is part funded by NIHR Oxford Biomedical Research Centre and Applied Research Centre.

SCr reports grants from NIHR HTA during the conduct of the study, and various other grants from NIHR and UK charities outside the submitted work. She is also Interim Co-Director (and previously Director) of the UKCRC-Registered Peninsula Clinical Trials Unit, which is in receipt of NIHR Clinical Trials Unit Support Funding (current award ends 31 August 2021).

LP reports consultancy fees from NIHR during the conduct of the study; grants from Living Streets Charity, personal fees from NIHR, personal fees from NIHR PHR, personal fees from NIHR PHR rapid response, grants from Wellcome Trust seed corn (internal funding) outside the submitted work; and discloses that the physical activity group in Sport and Health Sciences at the University of Exeter has a collaboration with Activinsights (the manufacturer of the physical activity monitor), to provide study design advice and data analysis, but that analysis of the physical activity data in the present study was not undertaken as part of that service.

JH has nothing to disclose.

All authors critically revised successive drafts of the manuscript and approved the final version.

Neither the study sponsor nor funder has a direct role in study design; collection, management, analysis, and interpretation of data; writing of the report; manuscript writing or dissemination of results. The funder will be informed of impending research output for this study.

Acknowledgements

For peer review only - http://bmjopen.bmj.com/site/about/guidelines.xhtml

This research has been conducted independently by researchers at the Universities of Plymouth, Oxford, Nottingham, and St George's London, with the support of Clinical Research Networks at each site and PenARC.
We appreciate the support of the study Sponsor, University Hospitals Plymouth NHS Trust.
The study was led by researchers and managed by the Peninsula Clinical Trials Unit (PenCTU) at the University of Plymouth. We are indebted to Dr Helen Hancocks for her contribution to the TARS study as PenCTU Clinical Trial Manager; sadly Helen passed away suddenly on 24th January 2019.
We thank Doug Webb (PenCTU Assistant Trial Manager) for helping to develop the protocol, and acknowledge Jonny Wilks (PenCTU Data Manager) for TARS data management.
Our thanks go to all organisations involved in recruitment, and especially those working in primary and secondary care and in local Stop Smoking Services. We thank James Davies and Will Moyle for preparing the search code for identifying potential participants within primary care.
We acknowledge the contribution of the health trainers in delivering the intervention: Alix Covenant, Stephanie Erivo, Gemma Fox, Alex Gude, Sarah Kennedy, Amanda Perry, Lucy Porter and Charlotte Wahlich.
We gratefully acknowledge the members of our Trial Steering Committee (TSC) and Data Monitoring Committee (DMC) for their valuable support and guidance. TSC members: Prof. Marcus Munafo (Chair), Dr Stephanie MacNeill, Prof. Amanda Amos and Dr Michael Callaghan (PPI representative). Sadly, Michael died having made a valuable contribution to the study. DMC members: Dr Rebecca Playle (Chair), Dr Charlie Foster and Dr Jamie Brown.
Our sincerest thanks go to all the participants involved in the trial.

Funding

This study is funded by the National Institute for Health Research (NIHR) HTA programme (project reference 15/111/01). The views expressed are those of the authors and not necessarily those of the NIHR or the Department of Health and Social Care.

Provenance and peer review

The NIHR (HTA no 15/111) identified the research question 'What is the clinical and cost-effectiveness of additional physical activity in tapered smoking reduction programmes?' as a priority in 2015. The current study was favourably reviewed by independent experts as an outline bid to the NIHR (HTA) in 2015, and as a full bid in 2016.

Data sharing statement This is an Open Access article distributed in accordance with the Creative Commons Attribution Non Commercial (CC BY-NC 3.0) licence, which permits others to distribute, remix, adapt, build on this work non-commercially, and licence their derivative works on different terms, provided the original work is properly cited and the use is non-commercial. http://creativecommons.org/licenses/by-nc/3.0/.

REFERENCES

1. GBD 2015 Risk Factors Collaborators. Global, regional, and national comparative risk assessment of 79 behavioural, environmental and occupational, and metabolic risks or clusters of risks, 1990-2015: a systematic analysis for the Global Burden of Disease Study 2015. *Lancet* 2016;388:1659-724.

For peer review only - http://bmjopen.bmj.com/site/about/guidelines.xhtml

2. Department of Health. Towards a Smokefree Generation: A Tobacco Control Plan for England London: Department of Health; 2017 [updated 13 January 2020. Available from: https://www.gov.uk/government/publications/towards-a-smoke-free-generation-tobacco-control-plan-for-england2020.
3. Office for National Statistics. Adult smoking habits in the UK 2018 [Available from: https://www.ons.gov.uk/peoplepopulationandcommunity/ healthandsocialcare/healthandlifeexpectancies/bulletins/ adultsmokinghabitsingreatbritain/2018.
4. NICE. NICE Public Health Guidance 10, Smoking Cessation Services. London, 2008.
5. Klemperer EM, Hughes JR, Naud S. Reduction in Cigarettes per Day Prospectively Predicts Making a Quit Attempt: A Fine-Grained Secondary Analysis of a Natural History Study. *Nicotine Tob Res* 2019;21(5):648-54. doi: 10.1093/ntr/nty056 [published Online First: 2018/03/27]
6. Lindson N, Klemperer E, Hong B, et al. Smoking reduction interventions for smoking cessation. *Cochrane Database Syst Rev* 2019;9:Cd013183. doi: 10.1002/14651858.CD013183.pub2 [published Online First: 2019/10/01]
7. Lindson-Hawley N, Hartmann-Boyce J, Fanshawe TR, et al. Interventions to reduce harm from continued tobacco use. *Cochrane Database Syst Rev* 2016;10:Cd005231. doi: 10.1002/14651858.CD005231.pub3 [published Online First: 2016/11/02]
8. NICE. Tobacco: harm-reduction approaches to smoking. NICE Public Health Guidance 45. London: NICE, 2013.
9. Klemperer E, Hughes J. Does the Magnitude of Reduction in Cigarettes Per Day Predict Smoking Cessation? A Qualitative Review. *Nicotine Tob Res* 2016;18(1):88-92. doi: doi: 10.1093/ntr/ntv058. Epub 2015 Mar 5.
10. West R, Mohr G, Proudfoot H, et al. Top-line findings on smoking in England from the Smoking Toolkit Study: UCL, 2019.
11. Gotts JE, Jordt SE, McConnell R, et al. What are the respiratory effects of e-cigarettes? *BMJ,* 2019;366:l5275. doi: 10.1136/bmj.l5275 [published Online First: 2019/10/02]
12. Taylor A, Thompson TP. Exercise in the prevention, treatment and management of substance use addictions. In: M W, H B, eds. Exercise and Mental Health: Neurobiological Mechanisms of the Exercise Effect on Depression, Anxiety, and Well-being. New York: Taylor and Francis 2018.
13. Tobacco Use and Dependence Guideline Panel. Treating Tobacco Use and Dependence: 2008 Update,. In: US Surgeon Gen Report, ed. Rockville (MD), 2008.
14. Ussher MH, Faulkner GEJ, Angus K, et al. Exercise interventions for smoking cessation. *Cochrane Database Syst Rev* 2019;2019(10) doi: 10.1002/14651858.CD002295.pub6 [published Online First: 2019/11/07]
15. Taylor AH, Thompson TP, Greaves CJ, et al. A pilot randomised trial to assess the methods and procedures for evaluating the clinical effectiveness and cost-effectiveness of Exercise Assisted Reduction then Stop (EARS) among disadvantaged smokers. *Health Technol Assess* 2014;18(4):1-324. doi: 10.3310/hta18040 [published Online First: 2014/01/18]
16. Thompson TP, Greaves CJ, Ayres R, et al. An Exploratory Analysis of the Smoking and Physical Activity Outcomes From a Pilot Randomized Controlled Trial of an Exercise Assisted Reduction to Stop Smoking Intervention in Disadvantaged Groups. *Nicotine Tob Res* 2016;18(3):289-97. doi: 10.1093/ntr/ntv099 [published Online First: 2015/05/15]
17. Williams G, Niemiec C, Patrick H, et al. The importance of supporting autonomy and perceived competence in facilitating long-term tobacco abstinence. *Ann Behav Med* 2009;37(3):315-24.
18. Haasova M, Warren FC, Ussher M, et al. The acute effects of physical activity on cigarette cravings: systematic review and meta-analysis with individual participant data. *Addiction* 2013;108(1):26-37. doi: 10.1111/j.1360-0443.2012.04034.x [published Online First: 2012/08/07]
19. Haasova M, Warren FC, Ussher M, et al. The acute effects of physical activity on cigarette cravings: exploration of potential moderators, mediators and physical activity attributes using individual participant data (IPD) meta-analyses. *Psychopharmacology (Berl)* 2014;231(7):1267-75. doi: 10.1007/s00213-014-3450-4 [published Online First: 2014/02/14]

For peer review only - http://bmjopen.bmj.com/site/about/guidelines.xhtml

20. Pisinger C, Jorgensen T. Waist circumference and weight following smoking cessation in a general population: the Inter99 study. *Prev Med* 2007;44(4):290-5. doi: 10.1016/j.ypmed.2006.11.015 [published Online First: 2007/01/16]
21. Filozof C, Fernandez Pinilla MC, Fernandez-Cruz A. Smoking cessation and weight gain. *Obes Rev* 2004;5(2):95-103. doi: 10.1111/j.1467-789X.2004.00131.x [published Online First: 2004/04/17]
22. Meyers AW, Klesges RC, Winders SE, et al. Are weight concerns predictive of smoking cessation? A prospective analysis. *J Consult Clin Psychol* 1997;65(3):448-52. [published Online First: 1997/06/01]
23. Aubin H, Farley A, Lycett D, et al. Weight gain in smokers after quitting cigarettes: meta-analysis. *Bmj* 2012;345(e4439) doi: doi: https://doi.org/10.1136/bmj.e4439
24. Parsons AC, Shraim M, Inglis J, et al. Interventions for preventing weight gain after smoking cessation. *Cochrane Database Syst Rev* 2009(1):Cd006219. doi: 10.1002/14651858.CD006219.pub2 [published Online First: 2009/01/23]
25. Oh H, Taylor AH. Self-regulating smoking and snacking through physical activity. *Health Psychol* 2014;33(4):349-59. doi: 10.1037/a0032423 [published Online First: 2013/05/15]
26. Chan AW, Tetzlaff JM, Altman DG, et al. SPIRIT 2013 statement: defining standard protocol items for clinical trials. *Ann Intern Med* 2013;158(3):200-7. doi: 10.7326/0003-4819-158-3-201302050-00583 [published Online First: 2013/01/09]
27. Hoffmann TC, Glasziou PP, Boutron I, et al. Better reporting of interventions: template for intervention description and replication (TIDieR) checklist and guide. *Bmj* 2014;348:g1687. doi: 10.1136/bmj.g1687 [published Online First: 2014/03/13]
28. Hancocks H, King J, Gude A, et al. The effect of the method of invitation on recruitment of participants from GP practices to a trial of a smoking reduction intervention: a study within a trial. International Clinical Trials Methodology Conference. Brighton, UK, 2019:P-143 https://az659834.vo.msecnd.net/eventsairwesteuprod/production-inconference-public/fe8d0a1d39e7403da5745e4b9fab416e.
29. Heatherton TF, Kozlowski LT, Frecker RC, et al. Measuring the heaviness of smoking: using self-reported time to the first cigarette of the day and number of cigarettes smoked per day. *Br J Addict* 1989;84(7):791-9. [published Online First: 1989/07/01]
30. Deci EL, Ryan RM. Intrinsic motivation and self-determination in human behaviour. New York: Plenum. 1985.
31. Deci E, Ryan R. The what and why of human goal pursuits: Human needs and the self-determination of behavior. . *Psychological Inquiry* 2000;11:227–68
32. Miller W, Rollnick S. Meeting in the middle: motivational interviewing and self-determination theory. *Int J Behav Nutr Phys Act* 2012;9(25) doi: 1479-5868-9-25 [pii] 10.1186/1479-5868-9-25
33. Miller W. Motivational Interviewing with problem drinkers. *Behavioural Psychotherapy,* 1983;11:147–72.
34. Markland D, Ryan R, Tobin V, et al. Motivational Interviewing and Self Determination Theory. *Journal Social and Clinical Psychology* 2005;24:811–31.
35. Thompson TP, Lambert J, Greaves C, et al. Intervention delivery fidelity assessment of a counselling based intervention for multiple behaviours. *Health Psychology* 2018;37(7):627-37.
36. Miller W, Rollnick S. Motivational interviewing: helping people change. New York: Guilford press 2012.
37. Deci EL, and Ryan RM. Handbook of self-determination research. Rochester, New York: University of Rochester Press 2002.
38. Cahill K, Stevens S, Perera R, et al. Pharmacological interventions for smoking cessation: an overview and network meta-analysis. *Cochrane Database Syst Rev* 2013(5):Cd009329. doi: 10.1002/14651858.CD009329.pub2 [published Online First: 2013/06/04]
39. West R, Hajek P, Stead L, et al. Outcome criteria in smoking cessation trials: proposal for a common standard. *Addiction* 2005;100:299-303.
40. Aveyard P, Wang D, Connock M, et al. Assessing the outcomes of prolonged cessation-induction and aid-to-cessation trials: floating prolonged abstinence. *Nicotine Tob Res* 2009;11(5) doi: doi: 10.1093/ntr/ntp035 [published Online First: 2009/04/07]

For peer review only - http://bmjopen.bmj.com/site/about/guidelines.xhtml

41. Jarvis MJ, Fidler J, Mindell J, et al. Assessing smoking status in children, adolescents and adults: cotinine cut-points revisited. *Addiction* 2008;103(9):1553-61. doi: 10.1111/j.1360-0443.2008.02297.x [published Online First: 2008/09/12]
42. Laugesen M, Epton M, Frampton C, et al. Hand-rolled cigarette smoking patterns compared with factory-made cigarette smoking in New Zealand men. *BMC Public Health* 2009;9(194)
43. Blair SN, Haskell WL, Ho P, et al. Assessment of habitual physical activity by a seven-day recall in a community survey and controlled experiments. *Am J Epidemiol* 1985;122(5):794-804. [published Online First: 1985/11/01]
44. Powell C, Carson BP, Dowd KP, et al. Simultaneous validation of five activity monitors for use in adult populations. *Scand J Med Sci Sports* 2017;27(12):1881-92. doi: 10.1111/sms.12813 [published Online First: 2016/12/03]
45. Herdman M, Gudex C, Lloyd A, et al. Development and preliminary testing of the new five-level version of EQ-5D (EQ-5D-5L). *Qual Life Res* 2011;20(10):1727-36. doi: 10.1007/s11136-011-9903-x [published Online First: 2011/04/12]
46. Ware J, Jr., Kosinski M, Keller SD. A 12-Item Short-Form Health Survey: construction of scales and preliminary tests of reliability and validity. *Med Care* 1996;34(3):220-33. [published Online First: 1996/03/01]
47. Callaghan L, Thompson TP, Creanor S, et al. Public Health Research. Individual health trainers to support health and well-being for people under community supervision in the criminal justice system: the STRENGTHEN pilot RCT. Southampton (UK): NIHR Journals Library 2019.
48. Ingram W, Webb D, Taylor RS, et al. Multicentred randomised controlled trial of an augmented exercise referral scheme using web-based behavioural support in individuals with metabolic, musculoskeletal and mental health conditions: protocol for the e-coachER trial. *BMJ Open* 2018;8(9):e022382. doi: 10.1136/bmjopen-2018-022382 [published Online First: 2018/09/24]
49. Thompson TP, Callaghan L, Hazeldine E, et al. A Health Trainer led motivational intervention plus usual care for people under community supervision compared to usual care alone: a study protocol for a parallel group pilot randomised controlled trial (STRENGTHEN). *Trials* 2018;4(8(6))::e023123.
50. Taggar JS, Lewis S, Docherty G, et al. Do cravings predict smoking cessation in smokers calling a national quit line: secondary analyses from a randomised trial for the utility of 'urges to smoke' measures. *Subst Abuse Treat Prev Policy* 2015;10:15. doi: 10.1186/s13011-015-0011-8 [published Online First: 2015/04/18]
51. van Hout B, Janssen MF, Feng YS, et al. Interim scoring for the EQ-5D-5L: mapping the EQ-5D-5L to EQ-5D-3L value sets. *Value Health* 2012;15(5):708-15. doi: 10.1016/j.jval.2012.02.008 [published Online First: 2012/08/08]
52. Brazier J, Ratcliffe J, Salomon J, et al. Measuring and Valuing Health Benefits for Economic Evaluation. Oxford: Oxford University Press 2007.
53. Kaltenthaler E, Tappenden P, Paisley S, et al. NICE DSU Technical Support Document 13: Identifying and reviewing evidence to inform the conceptualisation and population of cost-effectiveness models 2011 [Available from: nicedsu.org.uk.
54. Caro J, Briggs A, Siebert U, et al. Modeling Good Research Practices—Overview: A report of the ISPOR-SMDM Modeling Good Research Practices Task Force-1. *Value in Health* 2012;15:796-803.
55. Philips Z, Ginnelly L, Sculpher M, et al. Review of guidelines for good practice in decision-analytic modelling in health technology assessment. *Health Technology Assessment* 2004;8(36)
56. Schulz KF, Altman DG, Moher D, et al. CONSORT 2010 statement: updated guidelines for reporting parallel group randomised trials. *Bmj* 2010;340:c332. doi: 10.1136/bmj.c332 [published Online First: 2010/03/25]
57. Assmann SF, Pocock SJ, Enos LE, et al. Subgroup analysis and other (mis)uses of baseline data in clinical trials. *Lancet* 2000;355(9209):1064-9. doi: 10.1016/s0140-6736(00)02039-0 [published Online First: 2000/04/01]
58. Schulz KF, Altman DG, Moher D. CONSORT 2010 statement: updated guidelines for reporting parallel group randomized trials. *Ann Intern Med* 2010;152(11):726-32. doi: 10.7326/0003-4819-152-11-201006010-00232 [published Online First: 2010/03/26]

For peer review only - http://bmjopen.bmj.com/site/about/guidelines.xhtml

For peer review only

For peer review only - http://bmjopen.bmj.com/site/about/guidelines.xhtml

Figure 1 Participant flow chart

Figure 2 Indicative map of the TARS intervention components

For peer review only

Figure 1 Participant flow chart

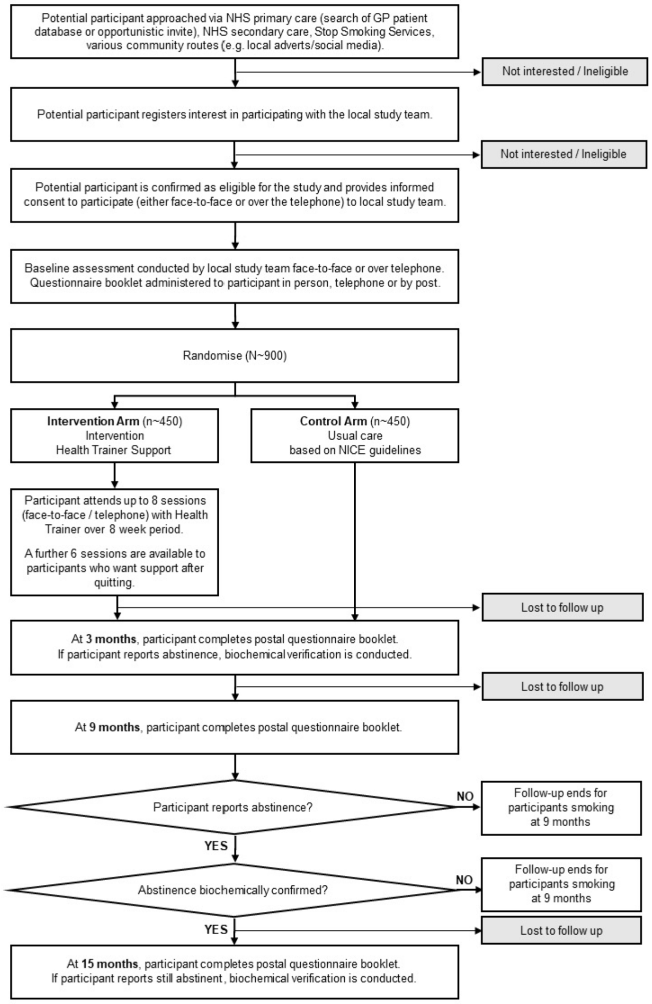

Caption : Figure 1 Participant flow chart

210x297mm (114 x 114 DPI)

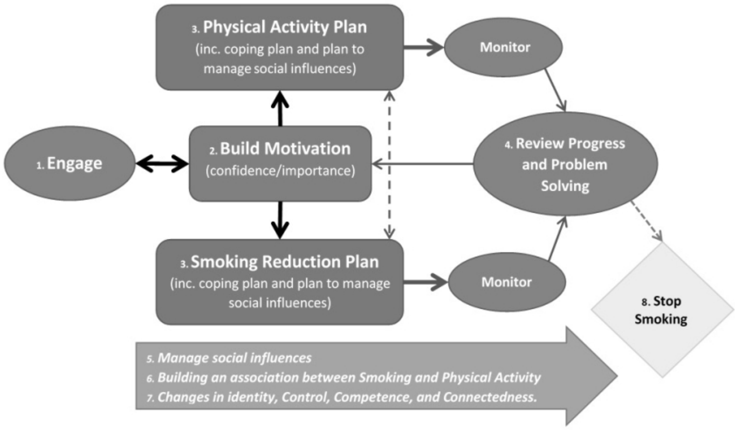

Figure 2 Indicative map of the TARS intervention components

215x279mm (150 x 150 DPI)

SPIRIT
STANDARD PROTOCOL ITEMS: RECOMMENDATIONS FOR INTERVENTIONAL TRIALS

SPIRIT 2013 Checklist: Recommended items to address in a clinical trial protocol and related documents*

Section/item	Item No	Description	Addressed on page number
**Administrative information**			
Title	1	Descriptive title identifying the study design, population, interventions, and, if applicable, trial acronym	1______________
Trial registration	2a	Trial identifier and registry name. If not yet registered, name of intended registry	1______________
	2b	All items from the World Health Organization Trial Registration Data Set	-
Protocol version	3	Date and version identifier	1_ V9.1 dated 11 June 2020
Funding	4	Sources and types of financial, material, and other support	6______________
Roles and responsibilities	5a	Names, affiliations, and roles of protocol contributors	6-7____________
	5b	Name and contact information for the trial sponsor	1, 6
	5c	Role of study sponsor and funders, if any, in study design; collection, management, analysis, and interpretation of data; writing of the report; and the decision to submit the report for publication, including whether they will have ultimate authority over any of these activities	37, 41, 52
	5d	Composition, roles, and responsibilities of the coordinating centre, steering committee, endpoint adjudication committee, data management team, and other individuals or groups overseeing the trial, if applicable (see Item 21a for data monitoring committee)	51 (oversight committees)

For peer review only

**Introduction**			
Background and rationale	6a	Description of research question and justification for undertaking the trial, including summary of relevant studies (published and unpublished) examining benefits and harms for each intervention	11-15
	6b	Explanation for choice of comparators	28
Objectives	7	Specific objectives or hypotheses	15-16
Trial design	8	Description of trial design including type of trial (eg, parallel group, crossover, factorial, single group), allocation ratio, and framework (eg, superiority, equivalence, noninferiority, exploratory)	16
**Methods: Participants, interventions, and outcomes**			
Study setting	9	Description of study settings (eg, community clinic, academic hospital) and list of countries where data will be collected. Reference to where list of study sites can be obtained	17, 20
Eligibility criteria	10	Inclusion and exclusion criteria for participants. If applicable, eligibility criteria for study centres and individuals who will perform the interventions (eg, surgeons, psychotherapists)	22
Interventions	11a	Interventions for each group with sufficient detail to allow replication, including how and when they will be administered	27-28
	11b	Criteria for discontinuing or modifying allocated interventions for a given trial participant (eg, drug dose change in response to harms, participant request, or improving/worsening disease)	27
	11c	Strategies to improve adherence to intervention protocols, and any procedures for monitoring adherence (eg, drug tablet return, laboratory tests)	
	11d	Relevant concomitant care and interventions that are permitted or prohibited during the trial	27-28
Outcomes	12	Primary, secondary, and other outcomes, including the specific measurement variable (eg, systolic blood pressure), analysis metric (eg, change from baseline, final value, time to event), method of aggregation (eg, median, proportion), and time point for each outcome. Explanation of the clinical relevance of chosen efficacy and harm outcomes is strongly recommended	9, 18-20, 32
Participant timeline	13	Time schedule of enrolment, interventions (including any run-ins and washouts), assessments, and visits for participants. A schematic diagram is highly recommended (see Figure)	21, 32

For peer review only - http://bmjopen.bmj.com/site/about/guidelines.xhtml

Sample size	14	Estimated number of participants needed to achieve study objectives and how it was determined, including clinical and statistical assumptions supporting any sample size calculations	41
Recruitment	15	Strategies for achieving adequate participant enrolment to reach target sample size	22-23
**Methods: Assignment of interventions (for controlled trials)**			
Allocation:			
Sequence generation	16a	Method of generating the allocation sequence (eg, computer-generated random numbers), and list of any factors for stratification. To reduce predictability of a random sequence, details of any planned restriction (eg, blocking) should be provided in a separate document that is unavailable to those who enrol participants or assign interventions	25
Allocation concealment mechanism	16b	Mechanism of implementing the allocation sequence (eg, central telephone; sequentially numbered, opaque, sealed envelopes), describing any steps to conceal the sequence until interventions are assigned	25
Implementation	16c	Who will generate the allocation sequence, who will enrol participants, and who will assign participants to interventions	25
Blinding (masking)	17a	Who will be blinded after assignment to interventions (eg, trial participants, care providers, outcome assessors, data analysts), and how	26
	17b	If blinded, circumstances under which unblinding is permissible, and procedure for revealing a participant's allocated intervention during the trial	N/A
**Methods: Data collection, management, and analysis**			
Data collection methods	18a	Plans for assessment and collection of outcome, baseline, and other trial data, including any related processes to promote data quality (eg, duplicate measurements, training of assessors) and a description of study instruments (eg, questionnaires, laboratory tests) along with their reliability and validity, if known. Reference to where data collection forms can be found, if not in the protocol	28-30, 32, 38-39
	18b	Plans to promote participant retention and complete follow-up, including list of any outcome data to be collected for participants who discontinue or deviate from intervention protocols	28-30

For peer review only - http://bmjopen.bmj.com/site/about/guidelines.xhtml

Data management	19	Plans for data entry, coding, security, and storage, including any related processes to promote data quality (eg, double data entry; range checks for data values). Reference to where details of data management procedures can be found, if not in the protocol	38-39
Statistical methods	20a	Statistical methods for analysing primary and secondary outcomes. Reference to where other details of the statistical analysis plan can be found, if not in the protocol	41-43
	20b	Methods for any additional analyses (eg, subgroup and adjusted analyses)	42-43
	20c	Definition of analysis population relating to protocol non-adherence (eg, as randomised analysis), and any statistical methods to handle missing data (eg, multiple imputation)	42-43
**Methods: Monitoring**			
Data monitoring	21a	Composition of data monitoring committee (DMC); summary of its role and reporting structure; statement of whether it is independent from the sponsor and competing interests; and reference to where further details about its charter can be found, if not in the protocol. Alternatively, an explanation of why a DMC is not needed	51
	21b	Description of any interim analyses and stopping guidelines, including who will have access to these interim results and make the final decision to terminate the trial	33
Harms	22	Plans for collecting, assessing, reporting, and managing solicited and spontaneously reported adverse events and other unintended effects of trial interventions or trial conduct	36-37
Auditing	23	Frequency and procedures for auditing trial conduct, if any, and whether the process will be independent from investigators and the sponsor	38
**Ethics and dissemination**			
Research ethics approval	24	Plans for seeking research ethics committee/institutional review board (REC/IRB) approval	52
Protocol amendments	25	Plans for communicating important protocol modifications (eg, changes to eligibility criteria, outcomes, analyses) to relevant parties (eg, investigators, REC/IRBs, trial participants, trial registries, journals, regulators)	52

For peer review only - http://bmjopen.bmj.com/site/about/guidelines.xhtml

Consent or assent	26a	Who will obtain informed consent or assent from potential trial participants or authorised surrogates, and how (see Item 32)	23-24
	26b	Additional consent provisions for collection and use of participant data and biological specimens in ancillary studies, if applicable	N/A
Confidentiality	27	How personal information about potential and enrolled participants will be collected, shared, and maintained in order to protect confidentiality before, during, and after the trial	40
Declaration of interests	28	Financial and other competing interests for principal investigators for the overall trial and each study site	-
Access to data	29	Statement of who will have access to the final trial dataset, and disclosure of contractual agreements that limit such access for investigators	40-41
Ancillary and post-trial care	30	Provisions, if any, for ancillary and post-trial care, and for compensation to those who suffer harm from trial participation	53
Dissemination policy	31a	Plans for investigators and sponsor to communicate trial results to participants, healthcare professionals, the public, and other relevant groups (eg, via publication, reporting in results databases, or other data sharing arrangements), including any publication restrictions	53
	31b	Authorship eligibility guidelines and any intended use of professional writers	53
	31c	Plans, if any, for granting public access to the full protocol, participant-level dataset, and statistical code	-
**Appendices**			
Informed consent materials	32	Model consent form and other related documentation given to participants and authorised surrogates	-
Biological specimens	33	Plans for collection, laboratory evaluation, and storage of biological specimens for genetic or molecular analysis in the current trial and for future use in ancillary studies, if applicable	N/A

*It is strongly recommended that this checklist be read in conjunction with the SPIRIT 2013 Explanation & Elaboration for important clarification on the items. Amendments to the protocol should be tracked and dated. The SPIRIT checklist is copyrighted by the SPIRIT Group under the Creative Commons "Attribution-NonCommercial-NoDerivs 3.0 Unported" license.

For peer review only
