# Supplementary material for: Randomised controlled trial of tailored support to increase physical activity and reduce smoking in smokers not immediately ready to quit: protocol for the Trial of physical Activity-assisted Reduction of Smoking (TARS) Study
Source: BMJ Open. 2020 Dec 1;10(12):e043331. doi: 10.1136/bmjopen-2020-043331 (PMC7709511; doi:10.1136/bmjopen-2020-043331)
Supplement: Reviewer comments [file bmjopen-2020-043331.reviewer_comments.pdf]

PEER REVIEW HISTORY

BMJ Open publishes all reviews undertaken for accepted manuscripts. Reviewers are asked to complete a checklist review form (**http://bmjopen.bmj.com/site/about/resources/checklist.pdf**) and are provided with free text boxes to elaborate on their assessment. These free text comments are reproduced below.

ARTICLE DETAILS

**TITLE (PROVISIONAL)**	A randomised controlled trial of tailored support to increase physical activity and reduce smoking in smokers not immediately ready to quit: Protocol for the Trial of physical Activity assisted Reduction of Smoking (TARS) study.
**AUTHORS**	Taylor, Adrian; Thompson, T; Ussher, Michael; Aveyard, Paul; Murray, Rachael L; Harris, Tess; Creanor, Siobhan; Green, Colin; Streeter, Adam Justin; Chynoweth, Jade; Ingram, Wendy; Greaves, Colin; Hancocks, Helen; Snowsill, Tristan; Callaghan, Lynne; Price, Lisa; Horrell, Jane; King, Jennie; Gude, Alex; George, Mary; Wahlich, Charlotte; Hamilton, Louisa; Cheema, Kelisha; Campbell, Sarah; Preece, Dan

This article was not externally reviewed at BMJ Open. Protocols that have been independently assessed prior to submission to BMJ Open are usually fast-tracked to publication on the grounds that further substantial changes will not be possible. This independent assessment will usually be external, independent review for both a substantial grant award from a non-commercial or government funder and ethics approval.
